# Geospatial variation in measles vaccine coverage through routine and campaign strategies in Nigeria: Analysis of recent household surveys

**DOI:** 10.1016/j.vaccine.2020.02.070

**Published:** 2020-03-23

**Authors:** C. Edson Utazi, John Wagai, Oliver Pannell, Felicity T. Cutts, Dale A. Rhoda, Matthew J. Ferrari, Boubacar Dieng, Joseph Oteri, M. Carolina Danovaro-Holliday, Adeyemi Adeniran, Andrew J. Tatem

**Affiliations:** a WorldPop, School of Geography and Environmental Science, University of Southampton, Southampton SO17 1BJ, UK; b Southampton Statistical Sciences Research Institute, University of Southampton, Southampton SO17 1BJ, UK; c World Health Organization Consultant, Abuja, Nigeria; d Department of Infectious Disease Epidemiology, London School of Hygiene and Tropical Medicine, London WC1E 7HT, UK; e Biostat Global Consulting, Worthington, OH, USA; f Center for Infectious Disease Dynamics, The Pennsylvania State University, State College, PA, 16802, USA; g GAVI Alliance, Abuja, Nigeria; h National Primary Health Care Development Agency, Abuja, Nigeria; i World Health Organization, Geneva, Switzerland; j National Bureau of Statistics, Abuja, Nigeria

**Keywords:** Measles vaccine, Supplementary immunization activities, Routine immunization, Geospatial analysis, Post-campaign coverage survey

## Abstract

Measles vaccination campaigns are conducted regularly in many low- and middle-income countries to boost measles control efforts and accelerate progress towards elimination. National and sometimes first-level administrative division campaign coverage may be estimated through post-campaign coverage surveys (PCCS). However, these large-area estimates mask significant geographic inequities in coverage at more granular levels. Here, we undertake a geospatial analysis of the Nigeria 2017–18 PCCS data to produce coverage estimates at 1 × 1 km resolution and the district level using binomial spatial regression models built on a suite of geospatial covariates and implemented in a Bayesian framework via the INLA-SPDE approach. We investigate the individual and combined performance of the campaign and routine immunization (RI) by mapping various indicators of coverage for children aged 9–59 months. Additionally, we compare estimated coverage before the campaign at 1 × 1 km and the district level with predicted coverage maps produced using other surveys conducted in 2013 and 2016–17. Coverage during the campaign was generally higher and more homogeneous than RI coverage but geospatial differences in the campaign’s reach of previously unvaccinated children are shown. Persistent areas of low coverage highlight the need for improved RI performance. The results can help to guide the conduct of future campaigns, improve vaccination monitoring and measles elimination efforts. Moreover, the approaches used here can be readily extended to other countries.

## Highlights

• Three surveys since 2013 show persistently low MCV coverage in much of northern Nigeria.
• The 2017–18 measles SIA reached higher and more homogeneous coverage than seen previously.
• Geospatial differences exist in the SIA’s reach of previously unvaccinated children.
• Nearly all the country is far from the goal of 95% coverage with ≥ 2 doses of MCV.
• Routine services need strengthening nationwide, especially in the north.

## 1 Introduction

Periodic supplementary immunization activities (SIAs), also known as vaccination campaigns, are a common strategy for increasing vaccination coverage, augmenting herd immunity and interrupting disease transmission in many low- and middle-income countries (LMICs) [1], [2]. Measles SIAs are designed to provide individuals who may have been missed by the routine immunization (RI) system with a second opportunity for vaccination [3], [4], with the target age range often informed by local measles epidemiology, timing and performance of previous campaigns, and RI performance. SIAs are usually conducted over a short period of time (typically up to one month) and use temporary additional vaccination posts to improve access to services and target all eligible children for vaccination, irrespective of their previous vaccination history [5]. Due to variable SIA performance, the cost of SIAs and debates about their potential to disrupt routine services [6], [7], increased focus is now being placed on demonstrating that SIAs do reach children missed by RI by measuring the proportion of children previously unvaccinated against measles (so-called MCV zero-dose children) who received a dose of measles-containing vaccine (MCV) during the SIA.

Estimates of SIA coverage can be produced using the administrative method – dividing the number of doses administered by the estimated target population, or through a high-quality post-campaign coverage survey (PCCS) [8], which could be designed to collect geocoded information and also provide information on previous vaccination(s) against measles through RI or earlier SIAs [5]. Administrative estimates of RI and SIA coverage suffer from a number of drawbacks, including inaccurate numerators and denominators; hence survey estimates may be more reliable. However, surveys are often designed to provide estimates at the national and first administrative division (province, county or state) levels mainly due to the high cost of more intensive sampling to produce estimates at more granular levels [8], [9], [10], [11]. Large-area estimates limit the potential to identify low coverage areas that would help improve targeting of efforts in subsequent vaccination activities - an important post-SIA activity that can benefit from high geographical precision.

Geospatial modelling approaches utilizing geolocated household survey data and relevant covariate information have become powerful tools for producing spatially detailed estimates and maps of vaccination coverage. Maps enable the identification of “coldspots” of low coverage, and data can be aggregated to different decision-making areas [12], [13], [14], [15], [16], [17]. Maps also serve as a flexible evidence base for integrating coverage estimates with other data sets [18] to produce additional programmatically-relevant metrics.

Measles is endemic in Nigeria, which had among the highest reported incidence of the disease globally during 2013–2018 [19]. Routine immunization against measles started in Nigeria in 1975 as part of the WHO Expanded Programme on Immunization (WHO EPI), but until 2017, WHO-UNICEF estimates of national immunization coverage were below 50% for almost all years [20] and a second MCV routine dose has not yet been introduced nationally. Following the roll-out of SIAs as part of global efforts to accelerate measles control and elimination [21], in 2005–2006, the country conducted a catch-up vaccination campaign targeting children aged 9 months to 14 years with administrative coverage of 95% of targeted population in northern and 83% in southern states [22]. Follow-up nationwide SIAs have since been held approximately every two years with the most recent national campaign occurring during 2017–18, targeting all children aged 9–59 months. These SIAs are also part of the efforts to achieve the measles elimination goal established by WHO African region in 2011, of which Nigeria was a signatory [23]. However, the continued occurrence of measles outbreaks [24] highlights the need for continuous assessment of the relative and combined performance of RI and SIAs to achieve effective measles control.

Here, we show the utility of geospatial techniques for producing subnational estimates of coverage post-SIA using the 2017–18 Nigeria PCCS data. We map five indicators that enable assessment of the performance of RI and SIAs across the country (see Table 1). We identify and map low coverage areas during the 2017–18 SIA, as well as the settlements within these areas. Furthermore, we compare estimates of coverage before and during the SIA from the PCCS with previous analyses using the 2013 Demographic and Health Survey (DHS) [12], [25] and the 2016–17 Multiple Indicator Cluster Surveys - National Immunization Coverage Survey (MICS-NICS) [26], [27] to examine trends in coverage at fine spatial scales and to assess how well questions in these surveys may have identified MCV zero-dose children.

**Table 1:** Vaccination coverage indicators analysed.

Indicator*	Definition	Remarks
1. Coverage before the SIA	Proportion of children aged 9–59 months at the time of the SIA who had a history of receipt of MCV before the SIA (according to vaccination card or parental recall)	Measures the combined effect of RI and previous SIAs on MCV coverage in the current SIA target population
2. SIA coverage among MCV zero-dose children	Proportion of children aged 9–59 months at the time of the SIA with NO history of receipt of MCV before the SIA who received a dose of MCV during the SIA (by card, finger-mark or parental recall)	Measures the effectiveness of the SIA in terms of reaching children most in need of MCV
3. SIA coverage among children vaccinated previously	Proportion of children aged 9–59 months at the time of the SIA with a history of receipt of MCV before the SIA who received a dose of MCV during the SIA (by card, finger-mark or parental recall)	Measures the effectiveness of the SIA in terms of reaching children who have already received at least one dose of MCV
4. Overall SIA coverage	Proportion of children aged 9–59 months at the time of the SIA who received a dose of MCV during the SIA (by card, finger-mark or parental recall)	Measures the effectiveness of the SIA in reaching all children in the target population
5. Coverage before and during the SIA	Proportion of children aged 9–59 months who had a history of MCV vaccination before the SIA AND who received a dose during the SIA	Measures coverage with at least 2 lifetime doses**of MCV by the end of the SIA Comparison of this indicator with indicator 2 shows how well the SIA reaches the hard-to-reach compared to children already vaccinated
6. Coverage before and/or during the SIA	Proportion of children aged 9–59 months who had a history of MCV vaccination before the SIA AND/OR who received a dose during the SIA	Measures coverage with at least one lifetime dose of MCV** by the end of the SIA

* Indicators 1–3 are the directly modelled indicators, while indicators 1, 2, 4–6 are the indicators of interest some of which were derived from the modelled indicators (see modelling section).

** Italicized words are synonyms for the indicators.

## 2 Methods

### 2.1 2017–18 post-campaign coverage survey data and Nigeria settlement data

The 2017–18 measles follow-up campaign was conducted during October - December 2017 in the northern states and February - March 2018 in the southern states. The reported administrative coverage estimate for the SIA was 107% at the national level, and between 91% and 131% at the state level. PCCS surveys were conducted 2–3 months after the SIA in each state. A two-stage sampling procedure was used which involved the selection of enumeration areas (EAs) (i.e. the survey clusters) from a national sampling frame and conducting household listing in each selected EA. Second stage selection of households to be interviewed was conducted by the National Bureau of Statistics (NBS) using simple random sampling without replacement from the list of households with eligible children aged 9–59 months. Seven (7) households with eligible children were randomly selected from each of the 30 EAs in every state and the Federal Capital Territory - Abuja (total 1110 EAs), and all children in the target age group in these households were eligible to be enrolled in the survey.

For each child, the age (in months) at the time of the survey and the centroid of the cluster from which the child’s household was selected were recorded alongside other relevant information. Data on MCV coverage before and during the campaign were obtained from home-based records (HBRs) of routine vaccination (and the separate vaccination card given out during the SIA) when available or through maternal/caregiver recall for children without cards. Based on these data, we analyzed six indicators assessing the individual and combined performance of RI and SIAs – see Table 1 for definitions. We note that only five of these indicators are of most interest in our work, but our modelling framework (see modelling section) required the inclusion of the additional indicator: *SIA coverage among children vaccinated previously*.

**Fig. 1:**
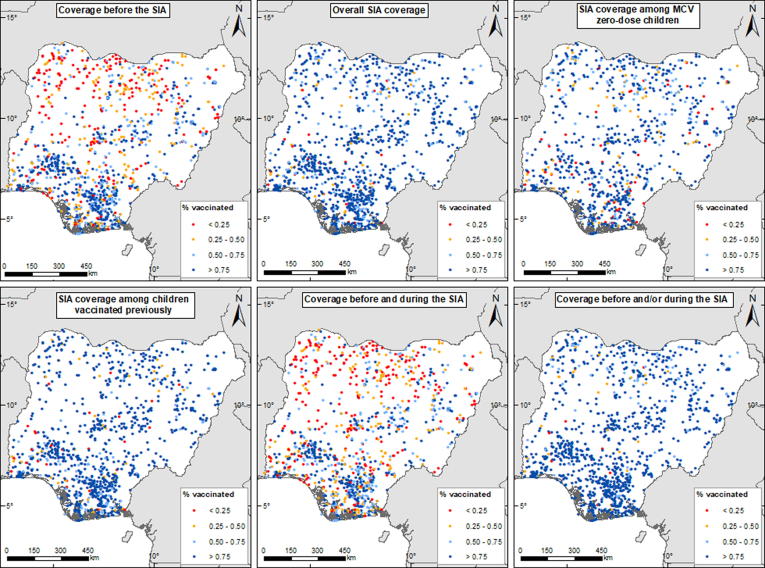
2017–18 post-campaign coverage survey cluster-level vaccination coverage data for children aged 9–59 months. The dots in the maps show the locations of the survey clusters.

The cluster-level vaccination coverage data for children aged 9–59 months are mapped in Fig. 1 for all six indicators. For *overall SIA coverage*, for example, a total of 9806 children were sampled, out of which 8671 were reportedly vaccinated in the SIA (excluding those with incomplete vaccination history or unknown vaccination status before or after the SIA or both). For all indicators, the numbers of children sampled at the cluster level ranged between 1 and 26, while the numbers vaccinated were between 0 and 25. We excluded clusters where one child was surveyed (between 1% and 19% of the total numbers of clusters - see supplementary Table 1) during model-fitting as this sample size often results in higher prediction uncertainty.

Further, to facilitate post-SIA targeting of MCV zero-dose populations, we obtained additional data on settlements within the country from the GRID3 program [20], [28], [29]. These settlements are polygons and points compiled from various vaccination programs within the country and other sources (see Fig. 3). Detailed information on the settlements is not included here for confidentiality reasons. Finally, to enable the production of coverage estimates for different administrative areas, we obtained relevant population data corresponding to the survey years from WorldPop (www.worldpop.org) [30].

### 2.2 Geospatial covariates and covariate selection

As in previous work [12], [14], [15], [16], we assembled a suite of geospatial socio-economic, environmental, and physical covariates for the PCCS analysis. These included datasets relating to remoteness, poverty, livestock, land cover and land surface temperature (supplementary Table 2 and supplementary Fig. 3). These covariates help improve the prediction of vaccination coverage and were considered for inclusion in the analysis because they either have been associated with the spatial distribution of vaccination coverage or serve as proxies for other factors known to influence coverage such as agricultural communities and some components of wealth. Using ESRI ArcGIS v10.6, a series of standardised gridded covariate layers were processed at 1 × 1 km for Nigeria from the original data sets. Following this, covariate data values were extracted from each of the standard layers for each PCCS cluster location.

Covariate selection was undertaken using similar approaches as outlined in Utazi et al [12] – see supplementary materials for details. For each of the three directly modelled indicators (see Table 1 and modelling section), this involved checking the relationships between the covariates and vaccination coverage and applying the log transformation to the covariates where necessary; fitting of single covariate models and ranking the covariates based on their predictive ability (i.e. using predictive R^2^ values); checking for multicollinearity and selecting between highly correlated covariates (correlation > 0.8 or variance inflation factor > 4.0) using their ranks; and using stepwise regression (backward elimination based on Akaike Information Criterion) to choose the best model/combination of covariates for modelling the indicator in a non-spatial framework using binomial regression models. From the set of covariates selected for each indicator, a final uniform set of covariates was formed to model all three indicators. This mainly included covariates that were significant in at least two of the three best models, except travel time to cities (urban accessibility) which was only significant in one of the models but was included in the analyses based on findings from previous work [12].

### 2.3 Modelling and prediction details

A geostatistical approach [31], [32] was adopted in this work to model vaccination coverage using the selected covariates and to make predictions in unsampled areas – typically 1 × 1 km grid cells covering Nigeria. For i=1,⋯,n and a given indicator, let $Y(s_{i})$ be the number of children vaccinated at cluster location $s_{i}$, out of a total of $N(s_{i})$ children sampled at the location. The first level of the geostatistical model can be expressed as: $Y\left(s_{i}\right)\sim Binomial\left(N\left(s_{i}\right),p\left(s_{i}\right)\right)$, where $p(s_{i})$ denotes vaccination coverage at location $s_{i}$. Further, the model assumes that

(1) $$logit\left(p(s_{i})\right)=x{\left(s_{i}\right)}^{T}\beta +\omega \left(s_{i}\right)+∊\left(s_{i}\right)$$

where $x(s_{i})$ is the vector of covariate data associated with $s_{i}$, β are the corresponding regression coefficients, $∊(s_{i})$ is an independent and identically distributed (iid) Gaussian random effect with variance, $\sigma_{∊}^{2}$, used to model non-spatial residual variation, and $\omega (s_{i})$ is a Gaussian spatial random effect used to capture residual spatial correlation in the model. That is, $\omega ={\left(\omega \left(s_{1}\right),\cdots ,\omega \left(s_{n}\right)\right)}^{T}\sim N\left(0,\Sigma_{\omega }\right)$. $\Sigma_{\omega }$ is assumed to follow the Matérn covariance function [33] given by $\Sigma_{\omega }\left(s_{i},s_{j}\right)=\frac{\sigma^{2}}{2^{\nu -1}\Gamma \left(\nu \right)}{\left(\kappa \|s_{i}-s_{j}\|\right)}^{\nu }K_{\nu }\left(\kappa \|s_{i}-s_{j}\|\right)$, where ‖.‖ denotes the Euclidean distance between cluster locations $s_{i}$ and $s_{j},\;\sigma^{2}>0$ is the marginal variance of the spatial process, κ is a scaling parameter related to the range $r(r=\frac{\sqrt{8\nu }}{\kappa })$ – the distance at which spatial correlation is close to 0.1, and $K_{\nu }$ is the modified Bessel function of the second kind and order ν>0. Further, for identifiability reasons, we set ν=1, see [34].

A Bayesian approach was adopted for fitting model (1), which was implemented using the integrated nested Laplace approximation – stochastic partial differential equation (INLA-SPDE) approach [34], [35] (see supplementary material for details).

To ensure the internal consistency of the indicators in Table 1 (i.e. that the modelled estimates retain the relationships among the indicators), we adopted the conditional probabilities approach (see, e.g., [15] and supplementary materials) and fitted model (1) for each of these indicators: (i) *coverage before the SIA* $\left(p_{B}\left(s\right)\right)$; (ii) *SIA coverage among MCV zero-dose children* $\left(p_{D|\bar{B}}\left(s\right)\right)$; and (iii) *SIA coverage among children vaccinated previously* $\left(p_{D|B}\left(s\right)\right)$. The fitted models were then used to predict vaccination coverage at 1 × 1 km. Using samples from the posterior predictive distributions of these directly modelled indicators, the 1 × 1 km estimates of the remaining three indicators of interest in Table 1 - *overall SIA coverage* $\left(p_{D}\left(s\right)\right)$, *coverage before and during the SIA* $\left(p_{D\cap B}\left(s\right)\right)$ and *coverage before and/or during the SIA* $\left(p_{D\cup B}\left(s\right)\right)$ - were calculated using the probability relations:

$$p_{D}\left(s\right)=p_{D|B}\left(s\right)\times p_{B}\left(s\right)+p_{D|\bar{B}}\left(s\right)\times p_{\bar{B}}\left(s\right);$$

$$p_{D\cap B}\left(s\right)=p_{D|B}\left(s\right)\times p_{B}\left(s\right),$$

(2) $$p_{D\cup B}\left(s\right)=p_{D|\bar{B}}\left(s\right)\times p_{\bar{B}}\left(s\right)+p_{B}\left(s\right);$$

for a given location s, with $p_{\bar{B}}\left(s\right)=1-p_{B}\left(s\right)$. For each of the five indicators of interest, the predictions were then aggregated to the local government areas (LGAs) and other relevant administrative levels through population-weighted Monte Carlo integration using the 1 × 1 km prediction grids, similar to *block averaging* [31].

The performance of the fitted models for out-of-sample prediction was assessed using a k-fold cross-validation scheme at the cluster level, with the cross-validation folds created as random and spatially stratified splits of the n cluster locations. Percentage bias (% Bias), root mean square error (RMSE) and the correlation between observed and predicted values were used to evaluate predictive performance (see supplementary materials for details). Additionally, for two of the indicators, the state and regional level modelled estimates were compared to direct estimates computed from the survey data (i.e. weighted proportions of the data). All analyses were carried out in R software [36] and using the R-INLA package [37].

We also undertook a re-analysis of previous work using 2013 DHS [12] and 2016–17 MICS-NICS (see supplementary materials) using the methodology described here (i.e. model (1) and the INLA-SPDE approach). As in the PCCS analyses, we included all surveyed children who were eligible to be vaccinated against measles and for whom information on MCV coverage was collected in both analyses. These were children aged 9–59 months for DHS and those aged 12–35 months for MICS-NICS. The 2013 DHS and the 2017–18 PCCS (*coverage before the SIA*) included children who may have participated in two national follow-up SIAs (see supplementary Fig. 1), while the 2016–17 MICS-NICS included children (12–35 months) who may have participated in the 2015–16 SIA – the survey was conducted within one year of the SIA. We note that the prediction covariates used in both analyses are the same as in previous analyses [12] and some of these are different from those selected for the PCCS analyses. Also, displaced survey cluster locations were used in both analyses but not in the PCCS analyses.

## 3 Results

### 3.1 Model fitting and validation

The covariates chosen for PCCS model fitting and prediction were: distance to the edge of cultivated areas (distance to ECA), settlement type, land surface temperature (temperature), travel time to a health facility (travel time to HF), enhanced vegetation index (EVI) and travel time to cities (urban accessibility). These are displayed in supplementary Fig. 3. Estimates of parameters of the fitted models are shown in Table 2 and Supplementary Tables 3 and 4 for the indicators modelled directly. These estimates show that all the covariates, except temperature and urban accessibility, were significant predictors of *coverage before the SIA*. For *SIA coverage among MCV zero-dose children*, there were only two significant predictors: distance to ECA and temperature. For *SIA coverage among children vaccinated previously*, urban accessibility was the only significant predictor. While distance to ECA and travel time to HF had consistent relationships (i.e. consistently positive and negative, respectively) with all three modelled indicators, the nature of the estimated relationships between settlement type, EVI, temperature and urban accessibility and the indicators varied. For settlement type, temperature and urban accessibility, the same relationships were also observed in single covariate non-spatial models, suggesting that this phenomenon may be due to differences in the spatial distributions of the indicators. For example, higher coverage estimated for urban settlements for *coverage before the SIA* and higher coverage in rural settlements for the other two modelled indicators measuring SIA coverage likely demonstrates the reach of the SIA in rural areas. Additionally, for EVI, the changing relationships may also be due to undetected collinearity - the estimated relationship between this covariate and *SIA coverage among MCV zero-dose children* in the spatial model was different from that of the single covariate model, although this relationship was not significant in the spatial model.

**Table 2:** Parameter estimates for *coverage before the SIA.*

Parameter	Mean	Std. Dev.	2.5%	50%	97.5%
Intercept	3.0461	4.6108	−5.6854	2.9316	12.4351
Distance to ECA	0.0736	0.0305	0.0138	0.0736	0.1334
Urban settlement	0.4108	0.1459	0.1251	0.4105	0.6980
EVI	4.6403	1.2408	2.1996	4.6419	7.0703
log(Travel time to HF)	−0.0682	0.0266	−0.1206	−0.0682	−0.0161
log(Temperature)	−0.4233	0.4802	−1.3999	−0.4113	0.4860
Urban accessibility	−0.0214	0.0332	−0.0868	−0.0214	0.0437
Spatial range ($\hat{r})$*	0.6369	0.0816	0.4916	0.6316	0.8116
Spatial variance (${\hat{\sigma }}^{2})$	2.1948	0.2990	1.6609	2.1762	2.8330
iid variance (${{\hat{\sigma }}_{∊}}^{2}$)	1.0364	0.1573	0.7618	1.0242	1.3782

* in decimal degrees.

Estimates of parameters of both the spatial and iid random effects were significant in all the fitted models, with the estimated spatial ranges falling between ≈46 km and ≈71 km, indicating the presence of residual spatial correlation in the models. Cluster-level model validation statistics are reported in supplementary Table 5. As expected, the fitted models performed better under the random cross-validation scheme compared to the spatially stratified scheme. For the random scheme, the RMSE and % Bias values of the fitted models range between 0.19 and 0.27, and between −0.04% and 1.52% respectively; whereas for the spatially stratified scheme, these were between 0.19 and 0.31 and between 1.04% and 1.96%. These values suggest that, in general, the predictions made using these models had small bias and reasonable amounts of error. In terms of predictive power, the results show that *coverage before the SIA* was better estimated, with correlations of 0.60 and 0.33 for the random and spatially-structured schemes, than the other two indicators modelled directly. For those two indicators, the estimated correlations were low (≤0.30), indicating that the covariates were less informative for both indicators and/or the effect of small cluster-level sample sizes (see discussion section). At the state and regional levels, a high degree of correspondence was seen between direct survey estimates and the modelled estimates (supplementary Fig. 5 and Tables 7 and 8). Although the effect of spatial smoothing may have been more apparent in the state level modelled estimates of *overall SIA coverage*, correlations of 0.93 and 0.87 were obtained at this level (the correlations were >0.99 at the regional level) between the modelled and survey estimates of *coverage before the SIA* and *overall SIA coverage*, respectively. This further supports the accuracy of the modelled estimates at these spatial scales.

### 3.2 Predicted vaccination coverage maps

**Fig. 2:**
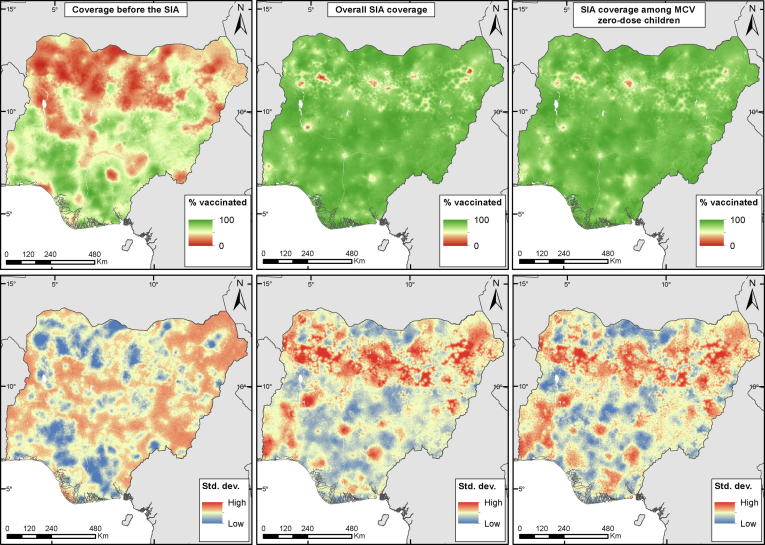
Predicted *coverage before the SIA* (top left), *overall SIA coverage* (top middle) and SIA *coverage among MCV zero-dose children* (top right) for children aged 9–59 months and the corresponding uncertainty estimates shown as standard deviations (bottom panels).

The 1 × 1 km predicted maps of three of the indicators of interest are shown in Fig. 2 for children aged 9–59 months, while those of the remaining two indicators of interest are shown in supplementary Fig. 4. Fig. 2 shows significant heterogeneities in the spatial distribution of *coverage before the SIA*, with most of the northern regions (north west, north east and north central) and parts of the south having poor coverage levels. The same pattern is also seen in *coverage before and during the SIA* (supplementary Fig. 4) which suggests greater likelihood of receipt of two doses in areas with higher coverage before the SIA. For the other three indicators - *overall SIA coverage*, *SIA coverage among MCV zero-dose children* and *coverage with at least one lifetime dose* – the predicted coverage rates were generally higher and more spatially homogeneous, although pockets of low coverage areas are evident in different parts of the country (see also district-level estimates in supplementary materials). In particular, aggregated estimates displayed in supplementary Fig. 8 reveal that areas where *SIA coverage among MCV zero-dose children* was lower relative to *overall SIA coverage* are located mostly in Kano, Oyo, Imo, Abia, Kaduna, Borno and Rivers states.

The patterns in the uncertainties (standard deviations) associated with the 1 × 1 km coverage estimates, as shown in the lower panels of Fig. 2 and supplementary Fig. 4, mostly reveal spots and areas of low uncertainties within and around areas with high density of survey clusters, and higher prediction error in un-sampled areas as expected. However, the occurrence of higher prediction uncertainty in un-sampled areas is more pronounced in *coverage before the SIA* and *coverage before and during the SIA*, both of which have higher overall uncertainty compared to the other indicators. This may be related to the distribution of coverage for both indicators as predicted values closer to 0 or 1 tend to have smaller standard deviations in binomial models. Further assessment of uncertainty at the cluster level for the directly modelled indicators revealed that it decreased as the number of eligible children interviewed within the clusters increased (see supplementary Fig. 11).

The attainment of at least 95% coverage with 2 doses of MCV in every district is considered necessary to achieve herd immunity against measles outbreaks [3]. The probabilities of achieving this coverage threshold are mapped in supplementary Fig. 6 for the three indicators assessing the combined effects of RI and SIAs: *coverage before the SIA*, *coverage with at least two lifetime doses* and *coverage with at least one lifetime dose*. For the first two indicators, these maps reveal a very low probability of reaching the threshold across almost all the country. However, there are areas and spots of higher probabilities particularly around urban centres in the south, and in some north central states. These areas can also be seen to have very high probabilities of attaining the threshold for *coverage with at least one lifetime dose* which has much larger areas of high probability. Despite this relative success in reaching children with at least one dose, many areas of suboptimal coverage remain across the country, even after the SIA, and these are concentrated in the north east and north western regions.

### 3.3 Mapping low coverage areas and settlements during the campaign

**Fig. 3:**
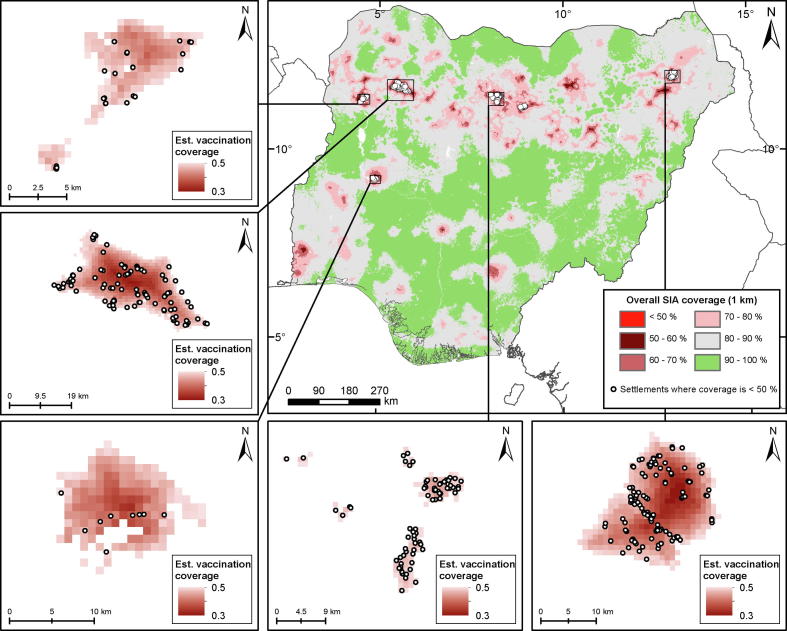
*Overall SIA coverage* with low coverage (≤50%) areas shown in red. The white dots indicate locations of settlements within low coverage areas. The insets (top left – bottom right) show low coverage areas and corresponding settlements in Kebbi, Kebbi/Zamfara, Niger, Kano/Kaduna and Borno states.

Identifying and delineating areas of lowest coverage is one of the key goals of analyses using PCCS data. Similar to previous work [12], [15], here we define these to be areas with ≤50% SIA coverage (other thresholds are possible). As shown in Fig. 3, these areas are concentrated mostly in the northern regions. In supplementary Fig. 7, PCCS cluster locations where ≤50% coverage was observed are overlaid on the 1 × 1 km predictions to examine how the predicted coverage levels mirror low coverage patterns seen in the data. The figure reveals that most of these low coverage clusters aligned better with areas where low coverage was predicted in the north compared to the south, indicating an over-prediction of coverage in the south for this indicator and the potential to miss MCV zero-dose populations in this region. Overall, the average difference between observed coverage at these cluster locations and predicted values extracted from the corresponding 1 × 1 km grid cells was 0.40 (s. d. = 0.19). This indicates a reasonable correspondence between the low coverage clusters and the predictions.

The locations of settlements falling within the low coverage areas, all of which are in the northern regions, are displayed in Fig. 3. The insets (top left-bottom right) show low coverage areas and corresponding settlements in Kebbi (24 settlements), Kebbi/Zamfara (98), Niger (9), Kano/Kaduna (73) and Borno (110) states. In all, 363 settlements were found, most of which are located in Borno (30.3%), Kano (29.2%) and Kebbi (18.2%) states.

### 3.4 Comparisons with 2013 DHS and 2016–17 MICS-NICS at the district level

**Fig. 4:**
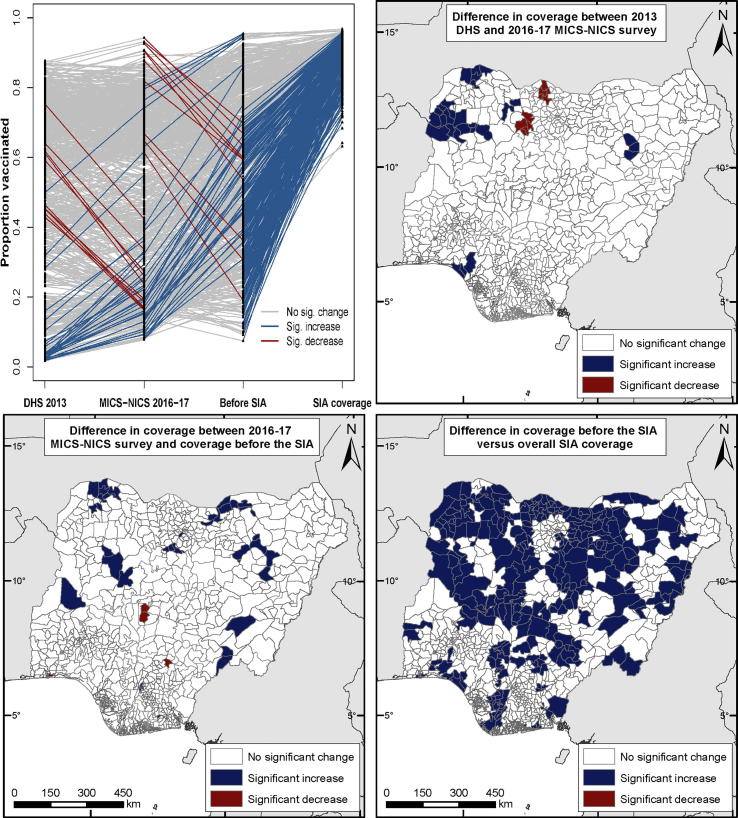
(Top left) Trends in MCV coverage at the LGA level between 2013 and 2017–18 predicted using 2013 DHS, 2016–17 MICS-NICS and 2017–18 PCCS (*coverage before the SIA* and *overall SIA coverage*) data. (Top right and bottom) Differences in MCV coverage at the LGA level between the surveys. Whether or not the differences/changes were significant was determined using the 95% credible intervals of the LGA estimates (see, e.g., supplementary Table 9).

Districts constitute the primary administrative division for many vaccination programs and often, coverage targets for RI and SIAs are also set and monitored at this level [23], [38], [39]. Here, we compare district (or local government area (LGA)) estimates of MCV coverage and associated uncertainties produced through using 2013 DHS (children aged 9–59 months) and 2016–17 MICS-NICS (children aged 12–35 months) with coverage estimates from the current analyses using 2017–18 PCCS (children aged 9–59 months) – see Fig. 4 and supplementary Figs. 9 and 10. Data on MCV receipt was mainly from caregiver recall (Supplementary Table 6) in each survey and while the exact phrasing of questions varied between surveys (see supplementary materials), none allowed distinction between receipt of MCV vaccination via routine or SIA strategies.

Fig. 4 (top left) shows fluctuating trends in estimated MCV coverage at the LGA level between 2013 and 2018. However, these changes were not significant (top right and bottom left) in 95% of the LGAs between 2013 and 2016–17, and 94% of the LGAs between 2016–17 and before the SIA. Furthermore, supplementary Fig. 9 reveals that much of the north, particularly the north east and north west, were consistent areas of low coverage between 2013 and before the 2017–18 SIA. Following the SIA, coverage increased in almost all (>97%) the LGAs. These increases were estimated to be significant in 37% of all the LGAs and are most marked in the northern regions (bottom right).

## 4 Discussion

To our knowledge, this work represents the first of its kind mapping the individual and combined performance of RI and SIAs using measles PCCS data at 1 × 1 km resolution. The combination of these vaccination strategies plays a critical role in measles control, and their effective use within countries is required to accelerate progress towards disease elimination goals. The geospatial analyses presented here therefore add to the growing evidence base of spatially detailed estimates of vaccination coverage to help improve program management in LMICs [12], [13], [14], [15], [16], [17].

Our analysis of *coverage before the SIA* revealed low coverage in most of the northern regions and parts of the south, as has also been suggested by other estimates of coverage [40], [41], [25], [26], [27]. This suboptimal RI system in these areas, in addition to high birth rates [40], results in a rapid accumulation of large numbers of susceptible individuals, particularly in the north. Lower MCV coverage could also reflect suboptimal performance of the 2015–16 SIA in many areas in the north although official reports do not show this [20]. On the other hand, our study has shown the effectiveness of the 2017–18 SIA in bridging gaps in coverage across the country through more widespread high coverage levels estimated for *overall SIA coverage* and *SIA coverage among MCV zero-dose children*, and as evidenced by the non-significant association of SIA coverage with travel time to a health facility despite being an important factor for routine coverage. However, we have also highlighted areas and LGAs with particularly low SIA coverage and lower MCV zero-dose SIA coverage.

Our evaluation of the combined effect of RI and SIAs revealed much of the north east and north west as well as some areas in the south as having very low probability of attaining the target of 95% coverage with at least one dose of MCV. Nearly all the country is far from the goal of 95% coverage with at least two doses of the vaccine through RI and SIA strategies. A second routine dose of MCV has been introduced in southern states at the end of 2019 and is planned in northern states in 2020. While routine services need strengthening nationwide to reach high coverage of two doses of MCV, the priority is for northern states to improve their routine services so that all children receive at least one routine dose as until then, SIAs will need to be done frequently to prevent outbreaks [42]. To facilitate the targeting of MCV zero-dose populations, we delineated poorly covered areas during the SIA - exploiting the geographical precision of the 1 × 1 km estimates - and identified the settlements within these areas, as well as the wards, LGAs and states where these could be found.

Consistent low coverage areas, most marked in the north, with few substantial improvements in coverage were observed at the LGA level between 2013 and before the 2017–18 SIA. We could not determine the extent to which information bias (and the different age groups) may have affected the estimated coverage in each survey. The questions posed in DHS and MICS-NICS should have captured SIA vaccinations but the low percentage giving a history of MCV receipt even in age cohorts that had been eligible for previous SIAs suggests that this was rarely included, reflecting either caregiver or interviewer information bias. Similarly, among children aged 35–39 months in the PCCS who had been eligible for at least one previous SIA, only 58% gave a history of past MCV receipt (data not tabulated), suggesting recall error and/or substantial inflation of past SIA coverages. This shows the difficulty in identifying and measuring SIA coverage among “MCV zero-dose” children in countries and areas where only a minority of children have vaccination records. The recurrence of measles outbreaks in many areas across the country in recent years despite these SIAs [24] emphasizes the need to improve RI for long-term measles control. In the interim, more frequent targeted SIAs as advocated in [42] could help maintain high coverage levels but revamping RI is still preferable as it utilises existing public health investments and also provides opportunity for administering other vaccines.

Our analyses are subject to some limitations. First, the cluster-level vaccination coverage data modelled in this work included information obtained from both vaccination cards and caregiver recall. In the PCCS analysis, vaccination records were not available for 70% and 41% of vaccinated children for *coverage before the SIA* and *overall SIA coverage*, both of which were measured directly during the survey. DHS and MICS-NICS also relied greatly on caregiver recall. This has the potential to introduce information bias in the analyses – see supplementary Table 6 and [43] for a review of the accuracy of sources of vaccination history. Secondly, some children included in the PCCS analyses, aged between 9 and 12 months, may have been too young to have received a dose prior to the SIA, though their contribution to coverage of the entire study population is unlikely to significantly affect the main results. Similarly, the different age group (12–35 months) studied in the MICS-NICS analysis may have affected comparison of the results with the other surveys although little variation in MCV coverage was reported by age cohort in these surveys. Thirdly, areas affected by conflict were excluded from each survey, in some instances after the first stage of cluster selection, hence the predictions were more uncertain in conflict-affected states (e.g. Borno state). Lastly, small cluster-level sample sizes, i.e. the numbers of children sampled within the clusters, reduce the predictive power of geostatistical models (especially when using a binomial likelihood) and could result in higher prediction uncertainty. Approximately 90% of the clusters included in the PCCS survey had sample sizes less than 15, with the average sample size being 9 children per cluster. In future surveys where geospatial analyses are planned, consideration should be given to increasing cluster sample sizes to boost prediction accuracy and enhance stratification of coverage by age. In practice, however, this may need to be balanced against design-based large-area survey analysis considerations, where large cluster sizes can be statistically inefficient. Alternatively, the use of a multinomial likelihood (see supplementary materials), as against the conditional probabilities approach using binomial likelihoods implemented here, may be a more effective way to utilize available cluster-level sample sizes during model-fitting. Further, the utility of geospatial analysis for identifying MCV zero-dose populations is largely dependent upon the ability of the fitted model(s) to predict low coverage areas (as highlighted previously) and how well the PCCS data captures such populations. There is the potential that specific populations that tend to be at high risk for un/under-vaccination are missed - mobile populations and those living in non-traditional or temporary accommodation or conflict areas (e.g. Borno State) - due to the failure of typical household listing processes in e.g. DHS, MICS and PCCS to capture these. Also, recording geospatial coordinates of temporary vaccination posts established for SIAs would make it possible to examine the effect of remoteness on campaign coverage and potentially help to show where more permanent vaccination posts or outreach sites should be established.

Although spatially detailed estimates are useful for understanding the spatial variation in coverage within countries, these are often triangulated with other data sets such as disease surveillance and population density estimates [18], [12], [13], [14] to produce additional metrics enabling the programmatic assessment of disease burden. Vaccination coverage is also explicitly linked with the occurrence of outbreaks [44] and the age profile of individuals affected during outbreaks. Characterizing and quantifying this linkage through a combined analysis of measles case-based surveillance data and the coverage estimates serves to shed more light on the impact of improvements in coverage on measles burden at flexible spatial scales. We plan to undertake these additional analyses and possibly comparisons of the estimates produced with routine coverage data elsewhere. Follow-up analyses will also focus on using individual level data to explore barriers to immunization in low coverage areas.

In conclusion, the methodology outlined here is readily scalable and opportunities exist for extension to other LMICs. PCCSs and other household surveys are undertaken in similar ways in these countries, but often only assess changes and coverage at broad spatial scales when there can be significant coldspots missed – thus underscoring the value of the proposed methods if elimination and eradication are to be achieved and limited resources used efficiently. Our analyses highlight that Nigeria should prioritize the improvement of its RI program (including increasing the availability and retention of vaccination records), especially but not only in the northern states, to be able to achieve its measles elimination goal but in the interim will continue to require SIAs with ongoing efforts to reach MCV zero-dose children.

## CRediT authorship contribution statement

**C. Edson Utazi:** Conceptualization, Methodology, Formal analysis, Writing - original draft, Writing - review & editing, Visualization. **John Wagai:** Investigation, Writing - review & editing. **Oliver Pannell:** Writing - review & editing, Visualization, Data curation. **Felicity T. Cutts:** Conceptualization, Methodology, Writing - original draft, Writing - review & editing. **Dale A. Rhoda:** Writing - review & editing, Investigation. **Matthew J. Ferrari:** Writing - review & editing. **Boubacar Dieng:** Writing - review & editing, Validation. **Joseph Oteri:** Writing - review & editing, Validation. **M. Carolina Danovaro-Holliday:** Conceptualization, Writing - review & editing. **Adeyemi Adeniran:** Writing - review & editing, Investigation. **Andrew J. Tatem:** Writing - original draft, Writing - review & editing.

## Declaration of Competing Interest

The authors declare that they have no known competing financial interests or personal relationships that could have appeared to influence the work reported in this paper. MCD-H works at the Wold Health Organisation. The comments on this article reflect those of the authors alone and do not necessarily reflect those of the World Health Organization.

## References

[1] Andrus Jon Kim, de Quadros Ciro A., Castillo Solórzano Carlos, Roses Periago Mirta, Henderson D.A. (2011). Measles and rubella eradication in the Americas. Vaccine.

[2] World Health Organization. Global measles and rubella strategic plan 2012–2020; 2012: Available from: http://apps.who.int/iris/bitstream/10665/44855/1/9789241503396_eng.pdf [accessed on 11 November 2019].

[3] World Health Organization. Measles vaccines: WHO position paper – April 2017. Weekly Epidemiol Rec 2017;92(205–28). 10.1016/j.vaccine.2017.07.066 28760612

[4] Portnoy A., Jit M., Helleringer S., Verguet S. (2018). Impact of measles supplementary immunization activities on reaching children missed by routine programs. Vaccine.

[5] World Health Organization. Planning and implementing high-quality supplementary immunization activities for injectable vaccines using an example of measles and rubella vaccines: Field guide; 2016 [accessed on 30 October 2019].

[6] Hanvoravongchai P., Mounier-Jack S., Oliveira Cruz V., Balabanova D., Biellik R., Kitaw Y. (2011). Impact of measles elimination activities on immunization services and health systems: findings from six countries. J Infect Dis.

[7] Verguet S., Jassat W., Bertram M.Y., Tollman S.M., Murray C.J.L., Jamison D.T. (2013). Impact of supplemental immunisation activity (SIA) campaigns on health systems: findings from South Africa. J Epidemiol Commun Health.

[8] World Health Organization. Vaccination Coverage Cluster Surveys: Reference Manual; 2018. Available from: https://www.who.int/immunization/documents/who_ivb_18.09/en/ [accessed on 15 October 2019].

[9] Subaiya S, Tabu C, N’ganga J, Awes AA, Sergon K, Cosmas L, et al. Use of the revised World Health Organization cluster survey methodology to classify measles-rubella vaccination campaign coverage in 47 counties in Kenya, 2016. PLOS ONE. 2018;13(7):e0199786. 10.1371/journal.pone.0199786 PMC6028100 29965975

[10] Cutts F.T., Claquin P., Danovaro-Holliday M.C., Rhoda D.A. (2016). Monitoring vaccination coverage: Defining the role of surveys. Vaccine.

[11] Danovaro-Holliday M.C., Dansereau E., Rhoda D.A., Brown D.W., Cutts F.T., Gacic-Dobo M. (2018). Collecting and using reliable vaccination coverage survey estimates: Summary and recommendations from the “Meeting to share lessons learnt from the roll-out of the updated WHO Vaccination Coverage Cluster Survey Reference Manual and to set an operational research agenda around vaccination coverage surveys”, Geneva, 18–21 April 2017. Vaccine.

[12] Utazi C.E., Thorley J., Alegana V.A., Ferrari M.J., Takahashi S., Metcalf C.J.E. (2018). High resolution age-structured mapping of childhood vaccination coverage in low and middle income countries. Vaccine.

[13] Takahashi Saki, Metcalf C. Jessica E., Ferrari Matthew J., Tatem Andrew J., Lessler Justin (2017). The geography of measles vaccination in the African Great Lakes region. Nat Commun.

[14] Utazi C.E., Thorley J., Alegana V.A., Ferrari M.J., Takahashi S., Metcalf C.J.E. (2019). Mapping vaccination coverage to explore the effects of delivery mechanisms and inform vaccination strategies. Nat Commun.

[15] Mosser J.F., Gagne-Maynard W., Rao P.C., Osgood-Zimmerman A., Fullman N., Graetz N. (2019). Mapping diphtheria-pertussis-tetanus vaccine coverage in Africa, 2000–2016: A spatial and temporal modelling study. Lancet.

[16] Mayala BK, Dontamsetti T, Fish TD, Croft TN. Interpolation of DHS survey data at subnational administrative Level 2. DHS Spatial Analysis Reports No 17. Rockville (Maryland, USA): ICF; 2019.

[17] Utazi C.E., Thorley J., Alegana V.A., Ferrari M.J., Nilsen K., Takahashi S. (2018). A spatial regression model for the disaggregation of areal unit based data to high-resolution grids with application to vaccination coverage mapping. Stat Meth Med Res.

[18] Tatem A.J. (2017). WorldPop, open data for spatial demography. Sci Data.

[19] World Health Organization. Measles and rubella surveillance data; 2019. Available from: https://www.who.int/immunization/monitoring_surveillance/burden/vpd/surveillance_type/active/measles_monthlydata/en/ [accessed on 12 November 2019].

[20] World Health Organization. WHO/UNICEF Estimates of National Immunization Coverage (WUENIC); 2019. Available from: https://www.who.int/immunization/monitoring_surveillance/data/en/ [accessed on 10 September 2019].

[21] Goodson J.L., Masresha B.G., Wannemuehler K., Uzicanin A., Cochi S. (2011). Changing epidemiology of measles in Africa. J Infect Dis.

[22] World Health Organization. Summary of measles-rubella supplementary immunization activities 2000–2019. Available from: https://www.who.int/immunization/monitoring_surveillance/data/en/ [accessed on 15 October 2019].

[23] WHO Regional Committee for Africa. Measles elimination by 2020: A strategy for the African Region; 2011. Available from: https://www.afro.who.int/about-us/governance/sessions/sixty-first-session-who-regional-committee-africa [accessed on 5 November 2019].

[24] Nigeria Centre for Disease Control. Weekly Epidemiological Report. Available from: https://ncdc.gov.ng/reports/weekly [accessed on 10 September 2019].

[25] National Population Commission - NPC/Nigeria, ICF International. Nigeria Demographic and Health Survey 2013. Abuja, Nigeria; 2014.

[26] National Bureau of Statistics (NBS) and United Nations Children’s Fund (UNICEF). 2017 Multiple Indicator Cluster Survey 2016–17, Survey Findings Report. Abuja (Nigeria): National Bureau of Statistics and United Nations Children’s Fund; 2017.

[27] National Primary Healthcare Development Agency and National Bureau of Statistics. Nigeria, National Immunisation Coverage Survey 2016/17, Final Report. Abuja (Nigeria): National Primary Healthcare Development Agency and National Bureau of Statistics; 2017.

[28] eHealth Africa, Oak Ridge National Laboratory, Proxy Logics. Geo-Referenced Infrastructure and Demographic Data for Development (GRID3) Nigeria Settlement Points; 2019.

[29] Weber Eric M., Seaman Vincent Y., Stewart Robert N., Bird Tomas J., Tatem Andrew J., McKee Jacob J., Bhaduri Budhendra L., Moehl Jessica J., Reith Andrew E. (2018). Census-independent population mapping in northern Nigeria. Remote Sens Environ.

[30] Tatem AJ. WorldPop, open data for spatial demography. Sci Data 2017;4(170004). 10.1038/sdata.2017.4 PMC5283060 28140397

[31] Banerjee S., Carlin B.P., Gelfand A.E. (2014). Hierarchical modeling and analysis for spatial data.

[32] Diggle P.J., Tawn J.A., Moyeed R.A. (1998). Model-based geostatistics. J Roy Stat Soc: Ser C (Appl Stat).

[33] Matérn B. Spatial variation. 2nd ed. Berlin: Springer-Verlag; 1960 [reprinted 1986].

[34] Lindgren F., Rue H., Lindström J. (2011). An explicit link between Gaussian fields and Gaussian Markov random fields: the stochastic partial differential equation approach. J Roy Stat Soc Series B (Stat Methodol).

[35] Rue H., Martino S., Chopin N. (2009). Approximate Bayesian inference for latent Gaussian models by using integrated nested Laplace approximations. J Roy Stat Soc: Series B (Stat Methodol).

[36] R Core Team. R: A Language and Environment for Statistical Computing. Vienna, Austria; 2017.

[37] Lindgren F., Rue H. (2015). Bayesian Spatial Modelling with R-INLA. J Stat Softw.

[38] World Health Organization. The RED strategy. Available from: http://www.who.int/immunization/programmes_systems/service_delivery/red/en/ [accessed on 15 October 2019].

[39] World Health Organization. Global Vaccine Action Plan 2011–2020; 2013. Available from: http://www.who.int/immunization/global_vaccine_action_plan/en/ [accessed on 20 June 2017].

[40] Masresha B, Braka F, Onwu NU, Oteri J, Erbeto T, Oladele S, et al. Progress towards measles elimination in Nigeria: 2012–2016. J Immunol Sci 2018;Suppl(135-9). PMC6446991 30957102

[41] Gunnala R., Ogbuanu I.U., Adegoke O.J., Scobie H.M., Uba B.V., Wannemuehler K.A. (2016). Routine vaccination coverage in Northern Nigeria: results from 40 district-level cluster surveys, 2014–2015. PLoS ONE.

[42] Zimmermann M., Frey K., Hagedorn B., Oteri A.J., Yahya A., Hamisu M. (2019). Optimization of frequency and targeting of measles supplemental immunization activities in Nigeria: A cost-effectiveness analysis. Vaccine.

[43] Dansereau Emily, Brown David, Stashko Lena, Danovaro-Holliday M. Carolina (2019). A systematic review of the agreement of recall, home-based records, facility records, BCG scar, and serology for ascertaining vaccination status in low and middle-income countries. Gates Open Res.

[44] Doshi R.H., Shidi C., Mulumba A., Eckhoff P., Nguyen C., Hoff N.A. (2015). The effect of immunization on measles incidence in the Democratic Republic of Congo: Results from a model of surveillance data. Vaccine.

