# Supplementary material for: Geospatial variation in measles vaccine coverage through routine and campaign strategies in Nigeria: Analysis of recent household surveys
Source: Vaccine. 2020 Mar 23;38(14):3062–71. doi: 10.1016/j.vaccine.2020.02.070 (PMC7079337; doi:10.1016/j.vaccine.2020.02.070)
Supplement: Supplementary data 1 [file mmc1.docx]

**Geospatial variation in measles vaccine coverage through routine and campaign strategies in Nigeria: analysis of recent household surveys**

C. Edson Utazi, John Wagai, Oliver Pannell, Felicity T. Cutts, Dale A. Rhoda, Matthew J. Ferrari, Boubacar Dieng, Joseph Oteri, M. Carolina Danovaro-Holliday, Adeyemi Adeniran, Andrew J. Tatem

**Supplementary material**

**Additional information on the 2017-18 measles post-campaign coverage survey and other surveys analysed**

The PCCS survey, like the DHS and MICS, was a two-stage cluster sample survey in which the National Population Commission master sampling frame from the 2006 Nigeria Population and Housing Census was used to select clusters (enumeration areas) using probability proportional to estimated size sampling. In each survey, inaccessible areas where there were on-going conflicts in the country were excluded, e.g. some local government areas in Borno and Adamawa states.

In each survey, household listing was conducted using standard procedures (see [www.dhsprogram.com](http://www.dhsprogram.com)) in each selected enumeration area (EA) from the first stage to map all the buildings and EA boundaries and identify households with eligible children aged < 5 years or 9-59 months for second stage selection using random sampling as described previously.

Standard questionnaires are shown in the relevant reports of the 2013 DHS (www.dhsprogram.com) and 2016-17 MICS surveys (https://mics.unicef.org/) surveys. The immunization section of the PCCS questionnaire included four main questions about measles vaccination:

- Did the child receive the measles vaccine during the recent campaign (Measles vaccination campaign in November/December 2017)? (possible answers Yes; No; Don’t know)
- Did the child receive a vaccination card after receiving the measles vaccine during the recent campaign? (possible answers Yes, card seen; Yes but card not seen; no; Don’t know)
- Was the finger of the child marked with a pen after receiving the measles vaccine during the campaign? (possible answers Yes, mark seen; Yes, but child not available to check; No; Don’t know)
- Before the campaign, had the child already received the measles vaccine? (possible answers: Yes, dates on card (follow-up questions then abstracted the date from the home based record (HBR) if available); Yes, recall/history; No; Don’t know)

Fingermark evidence of vaccination during the SIA was available for 13% of children who all had SIA vaccination cards hence we do not present those data separately. Regarding MCV vaccination before the 2017-18 SIA, 16.3% of all respondents had a HBR documenting prior receipt through routine services while 38.8% reported that they had received measles vaccine before the campaign from recall and for these children it is not known if they had been vaccinated through routine services or previous SIAs.

In Figure 1 below, we provide a timeline showing the implementation dates of all three surveys included in our study: the 2013 DHS, the 2016-17 MICS-NICS and the 2017-18 PCCS, their corresponding reference periods – these were the 5 years preceding DHS and PCCS given that our analyses included children aged <5 years for these surveys and the 35 months prior to the MICS-NICS survey, and national SIAs (follow-up SIAs only) that were conducted during these reference periods. These national SIAs targeted mostly children aged 9-59 months.

**
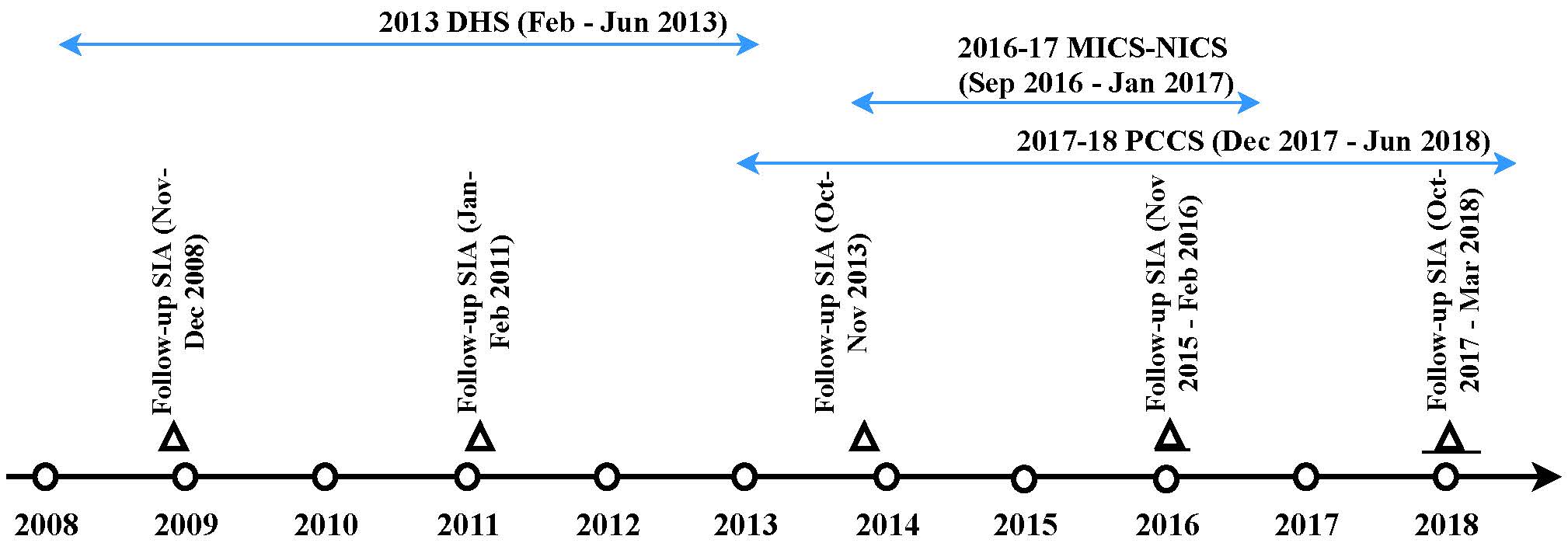
**

Figure 1: A timeline showing the surveys analyzed and SIAs occurring within their reference periods. Additional information on the SIAs are available from [https://www.who.int/immunization/monitoring_surveillance/data/en/](https://eur03.safelinks.protection.outlook.com/?url=https%3A%2F%2Fwww.who.int%2Fimmunization%2Fmonitoring_surveillance%2Fdata%2Fen%2F&data=01%7C01%7CC.E.Utazi%40soton.ac.uk%7C2d2ccf69328f4482a0da08d79d83fa20%7C4a5378f929f44d3ebe89669d03ada9d8%7C0&sdata=xrmM4Df9VlzOX8GiY24FKVK2MKwHLGdgR8CQJYUAaZg%3D&reserved=0). The implementation dates of the SIAs and the surveys are included in brackets.

The cohorts included in our analysis of the 2013 DHS had been eligible for one or two previous national SIAs while only part of the narrower age cohort included in the 2016-17 MICS-NICS survey had been eligible for the 2015-16 national SIA. The reference period for the 2017-18 PCCS encompassed three national SIAs, including the 2017-18 follow-up SIA following which the survey was conducted. These surveys are expected to have captured vaccinations that occurred during the respective SIAs, but the extent of information captured will depend on the reach of these SIAs, how well the sampling frames used in the surveys captured children that participated in the SIAs and the ability of mothers/caregivers to recall SIA vaccinations since these are not recorded on the HBRs used for routine vaccination and mothers were not asked to search for separate SIA cards for campaigns conducted before 2017. As we have noted previously, for DHS and MICS-NICS, information from HBRs will mostly be indicative of RI vaccination. However, for “recall” information, vaccination via past SIAs could not be distinguished from RI since the caregiver is simply asked whether the child had ever received measles vaccine. This is also the case for the indicator “coverage before the SIA” in the PCCS survey. Information on HBR/card availability during each survey is provided in supplementary Table 7. Although the indicator “coverage before the SIA” reflects vaccination through RI and SIAs, it was the closest indicator we had to assess RI performance in the PCCS as data on other vaccinations such as pentavalent vaccine given only through RI were not captured. The actual RI coverage would not exceed what was observed for this indicator.

**Additional information on modelling and prediction**

*Covariate selection for the PCCS analyses*

Covariate selection was undertaken in a non-spatial, frequentist framework using binomial generalized linear models (GLMs).This is a standard practice in spatial modelling, which is based on the notion that we want to account for as much variation (both spatial and non-spatial) in the data as possible using the available covariates before accounting for any unmeasured variation. A covariate selection procedure was implemented for each of the directly modelled indicators before a uniform set of covariates was formed for modelling all three indicators.

For each directly modelled indicator, the distributions of the covariates were examined, in relation to their relationships with vaccination coverage. Where evidence of skewness and non-linearity was seen, the log transformation was applied to improve the distributions of the covariates and encourage linearity. To guard against collinearity, correlations between the (continuous) covariates were calculated and we chose between highly correlated covariates ($\rho\geq0.80$) using out-of-sample R^2^ values of the corresponding single covariate binomial regression models. These out-of-sample R^2^ values were computed during a Monte Carlo cross-validation exercise repeated 10 times, with 20% of the data set aside each time for validation. Following this step, the variance inflation factors (VIF) [1] of the covariates were also calculated as an additional step to detect multicollinearity using a multiple binomial GLM. Again, the most important covariates among sets of correlated covariates with high VIF values (> 4.0) were selected using the R^2^ criterion. The stepwise regression approach, based on backward elimination and the AIC criterion, was used to determine the best combination of covariates for modelling each of the indicators. Finally, a uniform set of covariates for modelling all three indicators was created using covariates that were significant in at least two of the three best models from the stepwise regression step, except travel time to cities (urban accessibility) which was significant in only one of the best models but was included in the final set of covariates based on findings from previous work [2]. The use of a uniform set of covariates in the analyses enhances comparisons across the indicators.

*Additional information on the relationships between the PCCS indicators and the conditional probabilities modelling approach*

The combination of information on vaccination before and during the SIA from the PCCS survey results in four possible mutually exclusive outcomes for all surveyed children at any given location, $\boldsymbol{s}$, namely vaccination both before and during the SIA, vaccination before the SIA and not during the SIA, no vaccination before and vaccination during the SIA, and no vaccination both before and during the SIA. These outcomes are illustrated in the tree diagram below:

Vaccination during

the SIA

Outcome

Vaccination before

the SIA

$p_{D|B}\left( \boldsymbol{s} \right)$

$D\cap B$

$D$

$p_{B}\left( \boldsymbol{s} \right)$

$B$

$\bar{D}\cap B$

$\bar{D}$

$p_{\bar{D}|B}\left( \boldsymbol{s} \right)$

$p_{\bar{B}}\left( \boldsymbol{s} \right)$

$D\cap\bar{B}$

$D$

$p_{D|\bar{B}}\left( \boldsymbol{s} \right)$

$\bar{B}$

$p_{\bar{D}|\bar{B}}\left( \boldsymbol{s} \right)$

$\bar{D}\cap\bar{B}$

$\bar{D}$

Figure 2: A tree diagram showing possible combinations of vaccination outcomes before and during the SIA for a given survey location and some of the associated probabilities. The event $B$ denotes vaccination before the SIA while $\bar{B}$ denotes its complement. Similarly, $D$ denotes vaccination during the SIA and $\bar{D}$ its complement.

The probabilities corresponding to these outcomes sum to one (i.e. $p_{D\cap B}\left( \boldsymbol{s} \right)+p_{\bar{D}\cap B}\left( \boldsymbol{s} \right)+p_{D\cap\bar{B}}\left( \boldsymbol{s} \right)+p_{\bar{D}\cap\bar{B}}\left( \boldsymbol{s} \right)=p_{B}\left( \boldsymbol{s} \right)\times p_{D|B}\left( \boldsymbol{s} \right)+p_{B}\left( \boldsymbol{s} \right)\times p_{\bar{D}|B}\left( \boldsymbol{s} \right)+p_{\bar{B}}\left( \boldsymbol{s} \right)\times p_{D|\bar{B}}\left( \boldsymbol{s} \right)+p_{\bar{B}}\left( \boldsymbol{s} \right)\times p_{\bar{D}|\bar{B}}\left( \boldsymbol{s} \right)= 1$) and are related to the indicators of interest outlined in Table 1 (manuscript), as demonstrated in equation (2) using basic probability laws. In view of these interdependencies, it is therefore necessary that the indicators remain internally consistent (i.e. that these relationships are maintained) throughout the modelling process.

At the cluster level, this required that all child records with incomplete vaccination history (i.e. those with unknown vaccination status before or after the SIA or both) were excluded during the calculation of the directly modelled indicators as well as other indicators. Further, to ensure that the modelled estimates of the indicators of interest were internally consistent at every location – both the data and 1x1 km prediction locations, we adopted a conditional probability approach in which we modelled two conditional probabilities: *SIA coverage among zero-dose children* $\left( p_{D|\bar{B}}(\boldsymbol{s}) \right)$ and *SIA coverage among children vaccinated previously* $\left( p_{D|B}(\boldsymbol{s}) \right)$, and *coverage before the SIA (*$p_{B}\left( \boldsymbol{s} \right))$ directly, using the binomial geostatistical model given in equation 1. We then proceeded to calculate the 1x1 km estimates of the remaining indicators of interest from the corresponding estimates of these directly modelled indicators arithmetically as described in the modelling section.

We note that other modelling options that may guarantee the internal consistency of the indicators are possible, including a multinomial regression framework possibly involving the four mutually exclusive outcomes described earlier. We also note that fitting of independent geostatistical models to the indicators in Table 1 (manuscript) would have been a more straightforward modelling approach, but this approach does not guarantee the internal consistency of the modelled estimates, even when the modelled data satisfy the required constraints at the cluster level.

*Additional information on PCCS model fitting, prediction and validation*

To fit the model given in equation (1) in a Bayesian framework using the INLA-SPDE approach for the three directly modelled indicators, a non-informative $N({\boldsymbol{0},10}^{6}\boldsymbol{I})$ prior was placed on the regression coefficients, $\boldsymbol{\beta}$**.** A penalized complexity (PC) prior introduced in [3] was set on $\sigma_{\epsilon}$ such that $p\left( \sigma_{\epsilon}>0.5 \right)=0.01$. Similarly, following [4], a joint PC prior was placed on the covariance parameters of the spatial random effect, $\boldsymbol{\omega}$. These were: $p\left( r<r_{0} \right)=$0.01 and $p\left( \sigma>5 \right)=0.01$, with $r_{0}$ chosen to be 5% of the extent of Nigeria in the north-south direction, which expresses the intention of capturing a moderate level of spatial dependence. Also, the priors on $\sigma_{\epsilon}$ and $\sigma$ were chosen to favour the spatial random effects when apportioning residual variation in the model.

The SPDE approach involves a triangulation of the spatial domain in order to approximate $\boldsymbol{\omega.}$ A mesh was constructed for this approximation in each case using the survey cluster locations and the boundary points of Nigeria. The maximum triangle edge length was set to be 0.05 degrees in the inner mesh (which is much smaller than $r_{0}\approx0.48 \mathrm{degrees}$) and 0.6 degrees in the outer mesh. The choice of these edge lengths was guided by the need to maintain a balance between the accuracy of the approximation and computational costs.

From each of the fitted models, we generated 1000 samples from the posterior distributions of the parameters of the model, as well as from the posterior predictive distributions of vaccination coverage for each of the prediction locations, i.e. the 1x1 km grid cells. The latter were then used to calculate the estimates of the remaining indicators of interest at the grid level using the relationships shown in equation (2).

To assess the performance of the fitted models for out-of-sample prediction, we adopted the $k$-fold method of cross-validation, setting $k=15$. We created the cross-validation folds in two ways: (i) ‘random folds’ in which the $n$ cluster locations were randomly split into 15 cross-validation subsets, and (ii) ‘spatially stratified folds’ in which the cluster locations were separated into 15 subsets with neighbouring locations belonging to the same subset. The following model evaluation metrics were computed and averaged over the 15 subsets using the observed ($p(\boldsymbol{s})$) and predicted probabilities ($\hat{p}(\boldsymbol{s})$) for $m=n/15$ validation locations: percentage bias ($\% \mathrm{Bias}=100\times\sum_{i=1}^{m} (\hat{p}(\boldsymbol{s}_{i})-p(\boldsymbol{s}_{i}))/\sum_{i=1}^{m} p(\boldsymbol{s}_{i})$), root mean square error ($\mathrm{RMSE}=\sqrt{\sum_{i} \left( \hat{p}(\boldsymbol{s}_{i})-p(\boldsymbol{s}_{i}) \right)^{2}/m}$) and the Pearson’s correlation between the observed and predicted probabilities. The closer the values of $\% \mathrm{Bias}$ and RMSE are to zero, the better the predictions. In addition, correlation values close to one indicate better predictive ability.

*Additional information on the analysis of 2016-17 MICS-NICS and 2013 DHS data*

The 2016-17 MICS-NICS survey [5, 6] was conducted between September 2016 and January 2017 to collect data on a range of development indicators, including vaccination coverage. The processed data comprised 2,218 clusters (excluding those with incorrect geographical coordinates (163 clusters) and where one child was surveyed (321 clusters) – 484 clusters in all) from which 10,766 children aged 12-35 months were sampled. Out of this, 4,898 children were reportedly vaccinated against measles (as recorded on the child’s vaccination card or as reported by the mother). For the processed 2013 DHS data, there were 880 clusters where 23,514 children aged 9-59 months were surveyed, out of which 10,655 were vaccinated (by card or recall). The median cluster-level sample size for MICS-NICS was 5 (min. 2, max. 18), while that of the DHS was 24 (min. 3, max. 66). The analysis of MCV coverage using the MICS-NICS data utilized the same prediction covariates used in the analysis of the 2013 DHS, as described in Utazi et al [2]. A previous analysis of the MICS-NICS data also utilized the same modelling framework in [2]. However, to enhance comparability with the current PCCS analysis, we undertook a re-analysis of both the MICS-NICS and DHS data sets using the INLA-SPDE approach described in this work.

The predicted maps and corresponding uncertainty estimates for both surveys are shown in supplementary Figures 9 and 10. Based on the $k$- fold cross-validation with random folds, the out-of-sample RMSE, correlation and % Bias of the fitted models were 0.17, 0.82 and -1.53% for DHS, and 0.27, 0.68 and -0.9% for MICS-NICS, respectively.

**Supplementary Tables**

Table 1: Numbers of clusters where one child was surveyed for the directly modelled indicators

| Indicator | Total number of survey clusters* | Number of clusters where 1 child was surveyed |
| --- | --- | --- |
| Coverage before the survey | 1100 | 11 |
| SIA Coverage among MCV zero-dose children | 838 | 163 |
| SIA Coverage among children vaccinated previously | 1001 | 88 |

*****These do not include clusters with zero counts for the corresponding indicators. The 2017-18 PCCS survey had a total of 1110 clusters.

Table 2: Geospatial covariate layers considered for inclusion in the analysis (these were pre-selected from an initial list of 72 covariates).

| **S/N** | **Description** | **Unit** | **Year** | **Type** | **Source** |
| --- | --- | --- | --- | --- | --- |
| 1 | Average MODIS enhanced vegetation index from years 2000 to 2018 | - | 2000-2018 | Continuous | Didan, K. (2015). MOD13A3 MODIS/Terra vegetation Indices Monthly L3 Global 1km SIN Grid V006. NASA EOSDIS LP DAAC. |
| 2 | Cattle density | No. of cattle per sq km | 2010 | Continuous | Gilbert, M. *et al.*  (2018) Global Distribution Data for Cattle, Buffaloes, Horses, Sheep, Goats, Pigs, Chickens and Ducks in 2010. Nature Scientific data, 5:180227.[doi: 10.1038/sdata.2018.227](https://doi.org/10.1038/sdata.2018.227) |
| 3 | Chicken density | No. of chickens per sq km | 2010 | Continuous | Gilbert, M. *et al.*  (2018) Global Distribution Data for Cattle, Buffaloes, Horses, Sheep, Goats, Pigs, Chickens and Ducks in 2010. Nature Scientific data, 5:180227.[doi: 10.1038/sdata.2018.227](https://doi.org/10.1038/sdata.2018.227) |
| 4 | Goat density | No. of goats per sq km | 2010 | Continuous | Gilbert, M. *et al.*  (2018) Global Distribution Data for Cattle, Buffaloes, Horses, Sheep, Goats, Pigs, Chickens and Ducks in 2010. Nature Scientific data, 5:180227.[doi: 10.1038/sdata.2018.227](https://doi.org/10.1038/sdata.2018.227) |
| 5 | Distance to extrapolated built-settlement edges | Kilometres | 2017 | Continuous | WorldPop - School of Geography and Environmental Science, University of Southampton (2018). *Global High Resolution Population Denominators Project.* doi:[10.5258/SOTON/WP00645](https://www.worldpop.org/project/categories?id=14) |
| 6 | Distance to coastline | Kilometres | 2000-2020 | Continuous | WorldPop - School of Geography and Environmental Science, University of Southampton (2018). *Global High Resolution Population Denominators Project.* doi:[10.5258/SOTON/WP00645](https://www.worldpop.org/project/categories?id=14) |
| 7 | Distance to GHSL+ESA-CCI+GUF built-settlement area edges | Kilometres | 2014 | Continuous | WorldPop - School of Geography and Environmental Science, University of Southampton (2018). *Global High Resolution Population Denominators Project.* doi:[10.5258/SOTON/WP00645](https://www.worldpop.org/project/categories?id=14) |
| 8 | Distance to edges of cultivated areas | Kilometres | 2015 | Continuous | ESA (European Space Agency) CCI (Climate Change Initiative) Land Cover project 2017. "Land Cover CCI Product - Annual LC maps from 2000 to 2015 (v2.0.7).[" http://maps.elie.ucl.ac.be/CCI/viewer](http://maps.elie.ucl.ac.be/CCI/viewer) |
| 9 | Distance to sparse vegetation areas | Kilometres | 2015 | Continuous | ESA (European Space Agency) CCI (Climate Change Initiative) Land Cover project 2017. "Land Cover CCI Product - Annual LC maps from 2000 to 2015 (v2.0.7).[" http://maps.elie.ucl.ac.be/CCI/viewer](http://maps.elie.ucl.ac.be/CCI/viewer) |
| 10 | Distance to settlement/built-up areas | Kilometres | 2014 | Continuous | WorldPop - School of Geography and Environmental Science, University of Southampton (2018). *Global High Resolution Population Denominators Project.* doi:[10.5258/SOTON/WP00645](https://www.worldpop.org/project/categories?id=14) |
| 11 | Settlement/built-up areas (defined by GHSL and GUF) | - | 2014 | Binary | [Derived from] 1. Pesaresi, M. *et al.* (2015). *GHS built-up grid, derived from Landsat, multi-temporal (1975, 1990, 2000, 2014)* 2. Esch, T. *et al.* (2017). Breaking new ground in mapping human settlements from space – The Global Urban Footprint. *ISPRS Journal of Photogrammetry and Remote Sensing*, 134, 30–42. |
| 12 | Urban accessibility (M.A.P.) (travel time to cities where population >= 50,000 | Minutes | 2015 | Continuous | Weiss, D.J. *et al*. (2018). A global map of travel time to cities to access inequalities in accessibility in 2015. *Nature*. |
| 13 | VIIRS night-time lights | Nano-watts (sqcm*sr) | 2016 | Continuous | NOAA – Visible Infrared Imaging Radiometer Suite. <https://ngdc.noaa.gov/eog/viirs/index.html> |
| 14 | Distance to major OSM roads | Kilometres | 2016 | Continuous | [*Derived from*] Open Street Map (2019). © OpenStreetMap Contributors [www.openstreetmap.org](http://www.openstreetmap.org/) |
| 15 | Distance to OSM major waterways | Kilometres | 2016 | Continuous | [*Derived from*] Open Street Map (2019). © OpenStreetMap Contributors [www.openstreetmap.org](http://www.openstreetmap.org/) |
| 16 | Mean aridity index | - | 1950-2000 | Continuous | CGIAR-CSI Global-Aridity and Global-PET Database. Available at <https://cgiarcsi.community/data/global-aridity-and-pet-database/> |
| 17 | Average annual Potential evapotranspiration | Mm | 1950-2000 | Continuous | CGIAR-CSI Global-Aridity and Global-PET Database. Available at <https://cgiarcsi.community/data/global-aridity-and-pet-database/> |
| 18 | Digital elevation model | Metres | 2001 | Continuous | de Ferranti, J., 2017. 'Digital Elevation Data'. Viewfinder Panoramas [(www.viewfinderPanoramas.org/dem3.html](http://www.viewfinderpanoramas.org/dem3.html)); based on NASA's Shuttle Radar Topography Mission (SRTM) data ([http://www2.jpl.nasa.gov/srtm/)](http://www2.jpl.nasa.gov/srtm/) |
| 19 | WorldPop UN adjusted population density | People per sq km | 2000 | Continuous | WorldPop - School of Geography and Environmental Science, University of Southampton (2018). *Global High Resolution Population Denominators Project.* doi:[10.5258/SOTON/WP00645](https://www.worldpop.org/doi/10.5258/SOTON/WP00645) |
| 20 | MODIS mean land surface temperature | Kelvin (scaled 0.02) | 2001-2005 | Continuous | Wan, Z. *et al*. MOD11C3 MODIS/Terra Land Surface Temperature/Emissivity Monthly L3 Global 0.05Deg CMG Voo6 [Data set]. NASA EOSDIS Land Processes DAAC. |
| 21 | Urban areas (GUF) | - | 2000/2001 | Binary | Esch, T. *et al.* (2017). Breaking new ground in mapping human settlements from space – The Global Urban Footprint. *ISPRS Journal of Photogrammetry and Remote Sensing*, 134, 30–42. [doi:10.1016/j.isprsjprs.2017.10.012](https://doi.org/10.1016/j.isprsjprs.2017.10.012) |
| 22 | Economic index | - | N/A | Continuous | G-Econ Project, Yale University. (2009). <https://gecon.yale.edu/> |
| 23 | Travel time to health facilities | Minutes | 2018 | Continuous | [*Produced from locations of health facilities in Nigeria using the methodology in*] Weiss, D.J. *et al*. (2018). A global map of travel time to cities to access inequalities in accessibility in 2015. *Nature*. |
| 24 | Distance to OSM rivers | Kilometres | N/A | Continuous | [*Derived from*] Open Street Map (2019). © OpenStreetMap Contributors [www.openstreetmap.org](http://www.openstreetmap.org/) |
| 25 | Pig density | No. Of pigs per sq km | 2010 | Continuous | Gilbert, M. *et al.*  (2018) Global Distribution Data for Cattle, Buffaloes, Horses, Sheep, Goats, Pigs, Chickens and Ducks in 2010. Nature Scientific data, 5:180227.[doi: 10.1038/sdata.2018.227](https://doi.org/10.1038/sdata.2018.227) |
| 26 | Sheep density | No. Of sheep per sq km | 2010 | Continuous | Gilbert, M. *et al.*  (2018) Global Distribution Data for Cattle, Buffaloes, Horses, Sheep, Goats, Pigs, Chickens and Ducks in 2010. Nature Scientific data, 5:180227.[doi: 10.1038/sdata.2018.227](https://doi.org/10.1038/sdata.2018.227) |
| 27 | Poverty as defined by $ 2.00 | Proportion of people living in poverty | 2013 | Continuous | Tatem, A.J. *et al.* (2013). Pilot high resolution poverty maps. University of Southampton/Oxford University |
| 28** | Ethnicity for 5 groups across Nigeria as extract from the GeoEPR database (1000 - Hausa-Fulani & Muslim middle belt, 2000 - Igbo, 3450 - Ijaw, Ogoni & Tiv, 6000 - Yoruba, 7000 - Others/Unknown) | - | 2018 | Categorial | Wucherpfennig, J. *et al*. (2011). Politically relevant ethnic groups across space and time: Introducing the GeoEPR dataset. Conflict Management and Peace Science, Forthcoming. |
| 29** | Geopolitical zones of Nigeria (1 - North central, 2 - North East, 3- North West, 4 - South East, 5- South South, 6 - South West) | - | 2019 | Categorical | DHS (2018). Nigeria 2018 DHS subnational areas. DHS spatial data repository. <https://spatialdata.dhsprogram.com/boundaries/#view=table&countryId=NG> |

** Excluded from the covariates selection process. The inclusion of these covariates in the analyses obscured the underlying patterns in the data, in addition to producing unappealing patterns in the predicted maps which were difficult to explain and which did not align with the administrative structure of the country, as expected.

Table 3: Parameter estimates for *SIA* *coverage among MCV zero-dose children*

| Parameter | Mean | Std. Dev. | 2.5% | 50% | 97.5% |
| --- | --- | --- | --- | --- | --- |
| Intercept | -22.3385 | 11.1107 | -45.5613 | -21.8496 | -1.8740 |
| Distance to ECA | 0.1128 | 0.0394 | 0.0362 | 0.1126 | 0.1908 |
| Urban settlement | -0.3655 | 0.2409 | -0.8390 | -0.3655 | 0.1073 |
| EVI | -0.6212 | 1.8095 | -4.1830 | -0.6196 | 2.9278 |
| log(Travel time to HF) | -0.0523 | 0.0406 | -0.1325 | -0.0521 | 0.0270 |
| log(Temperature) | 2.6059 | 1.1564 | 0.4735 | 2.5555 | 5.0198 |
| Urban accessibility | 0.0213 | 0.0511 | -0.0792 | 0.0213 | 0.1215 |
| Spatial range ($\hat{r})$* | 0.4128 | 0.0837 | 0.2573 | 0.4113 | 0.5812 |
| Spatial variance ($\hat{\sigma}^{2})$ | 2.7356 | 0.4198 | 1.9628 | 2.7209 | 3.6034 |
| iid variance (${\hat{\sigma}_{\epsilon}}^{2}$) | 0.5205 | 0.2632 | 0.1581 | 0.4722 | 1.1654 |

*in decimal degrees

Table 4: Parameter estimates for *SIA coverage among children vaccinated previously*

| Parameter | Mean | Std. Dev. | 2.5% | 50% | 97.5% |
| --- | --- | --- | --- | --- | --- |
| Intercept | 12.2334 | 7.8497 | -1.3796 | 11.5693 | 29.4276 |
| Distance to ECA | 0.0027 | 0.0474 | -0.0913 | 0.0030 | 0.0950 |
| Urban settlement | -0.1675 | 0.2292 | -0.6174 | -0.1677 | 0.2830 |
| EVI | -1.2492 | 1.6044 | -4.3936 | -1.2528 | 1.9110 |
| log(Travel time to HF) | -0.0448 | 0.0451 | -0.1338 | -0.0446 | 0.0434 |
| log(Temperature) | -0.8838 | 0.8173 | -2.6722 | -0.8155 | 0.5326 |
| Urban accessibility | 0.1153 | 0.0522 | 0.0141 | 0.1148 | 0.2191 |
| Spatial range ($\hat{r})$* | 0.5000 | 0.1630 | 0.2488 | 0.4770 | 0.8831 |
| Spatial variance ($\hat{\sigma}^{2})$ | 1.6813 | 0.5282 | 0.8606 | 1.6090 | 2.9170 |
| iid variance (${\hat{\sigma}_{\epsilon}}^{2}$) | 1.4277 | 0.4966 | 0.7113 | 1.3392 | 2.6396 |

*in decimal degrees

Table 5: Cluster-level validation statistics - based on a $k$-fold cross-validation exercise with random and spatially structured folds.

| **Modelled indicator** | **Random** | | | **Spatially structured** | | |
| --- | --- | --- | --- | --- | --- | --- |
|  | **RMSE** | **Correlation** | **% Bias** | **RMSE** | **Correlation** | **% Bias** |
| Coverage before the SIA | 0.27 | 0.60 | -0.04 | 0.31 | 0.33 | 1.77 |
| SIA coverage among children vaccinated previously | 0.19 | 0.16 | 1.27 | 0.19 | 0.10 | 1.04 |
| SIA coverage among MCV zero-dose children | 0.25 | 0.30 | 1.52 | 0.26 | 0.15 | 1.96 |

Table 6: Percentage of vaccinated children whose MCV vaccination status were determined through a vaccination card according to the different surveys

| **Survey** | **Percentage** |
| --- | --- |
| DHS 2013 | 36.1% |
| MICS-NICS 2016-17 | 46.3% |
| PCCS 2017-18 (Coverage before the SIA) | 29.7% |
| PCCS 2017-18 (Overall SIA coverage) | 59.2% |

**Supplementary Figures**


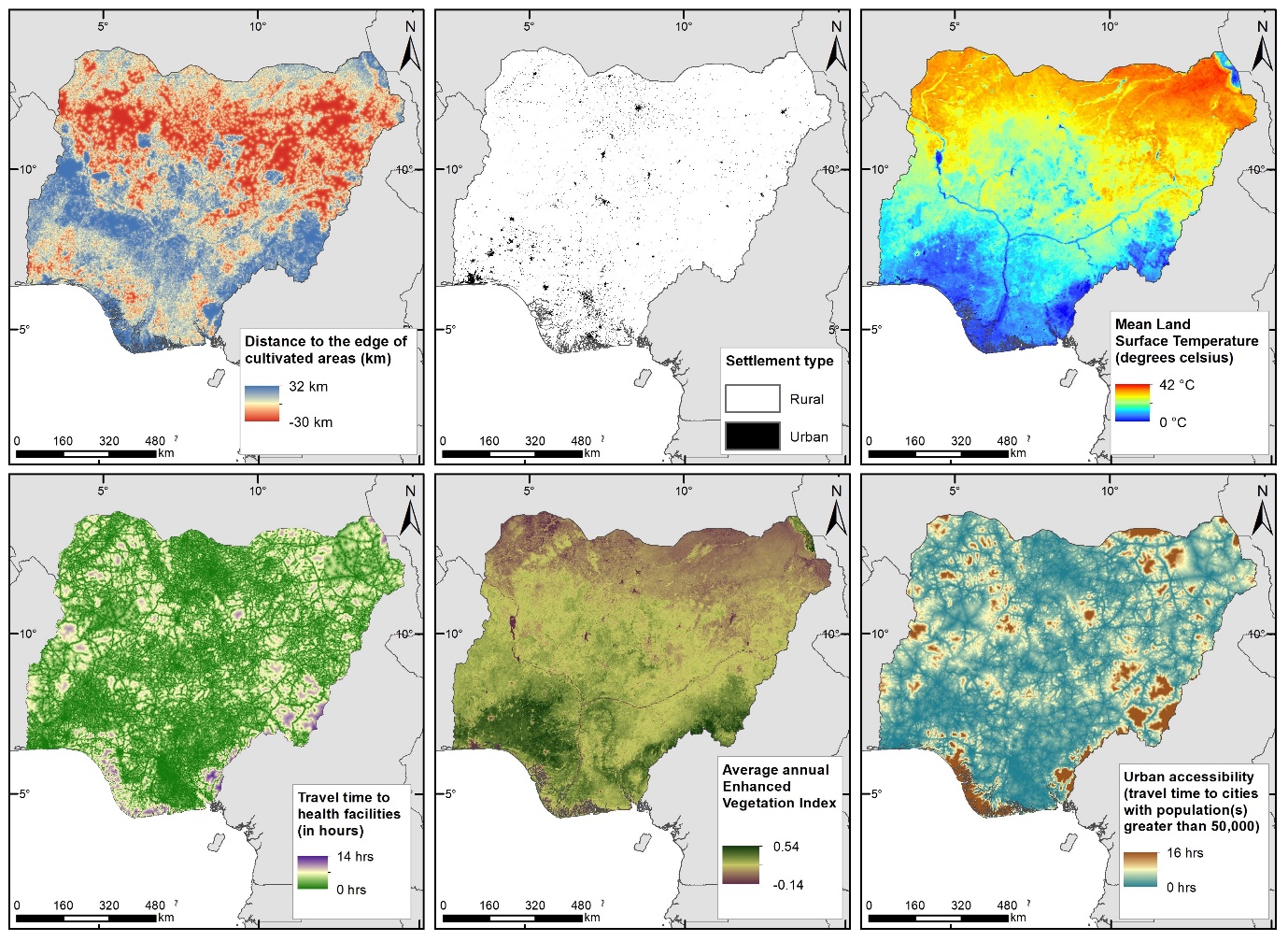


Figure 3: Selected geospatial covariates used for prediction. Additional information on these covariates is provided in Supplementary Table 2.


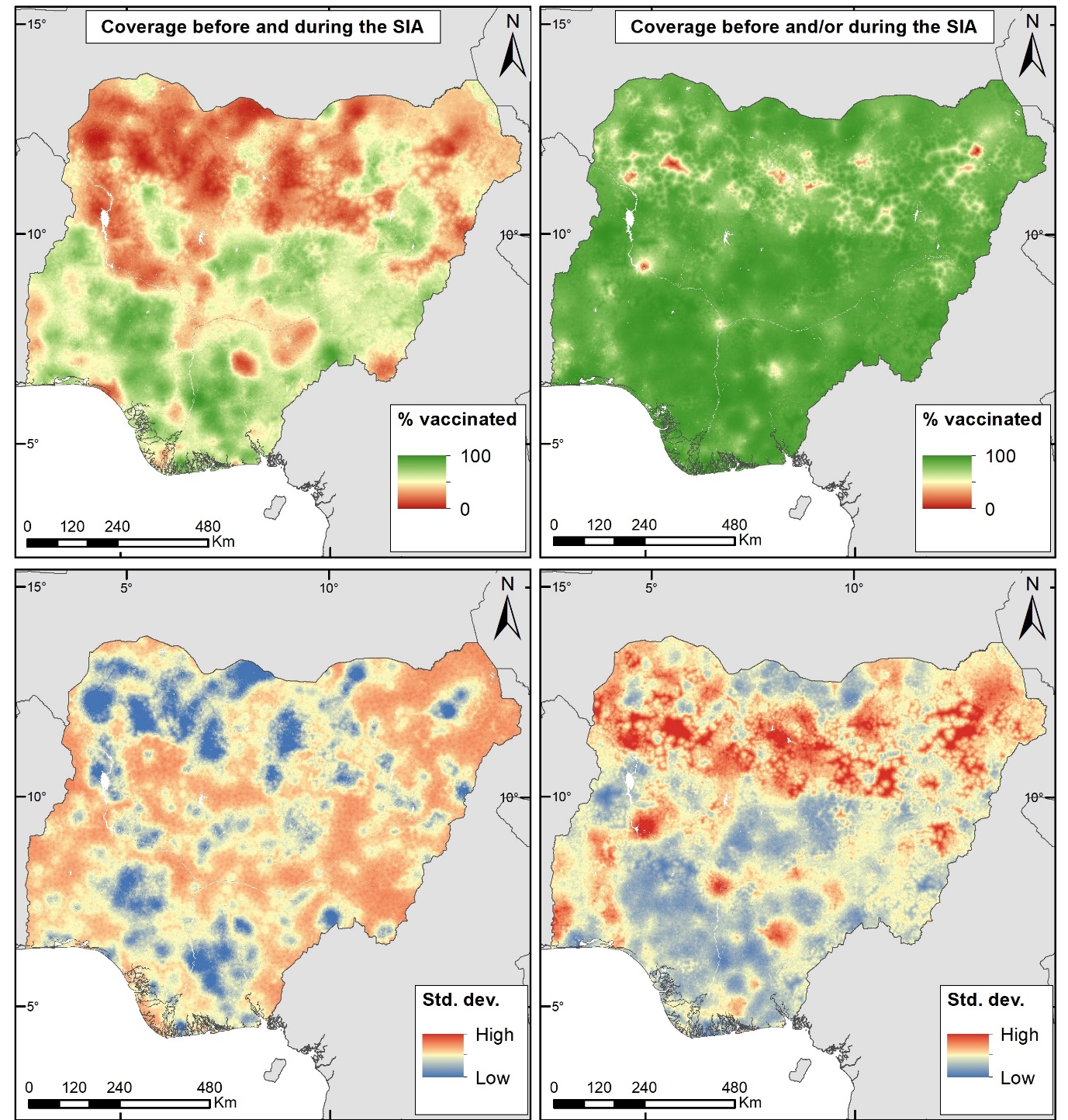


Figure 4: Predicted *coverage* *before and during the SIA* and *coverage* *before and/or during the SIA*, and the corresponding uncertainty estimates shown as standard deviations.


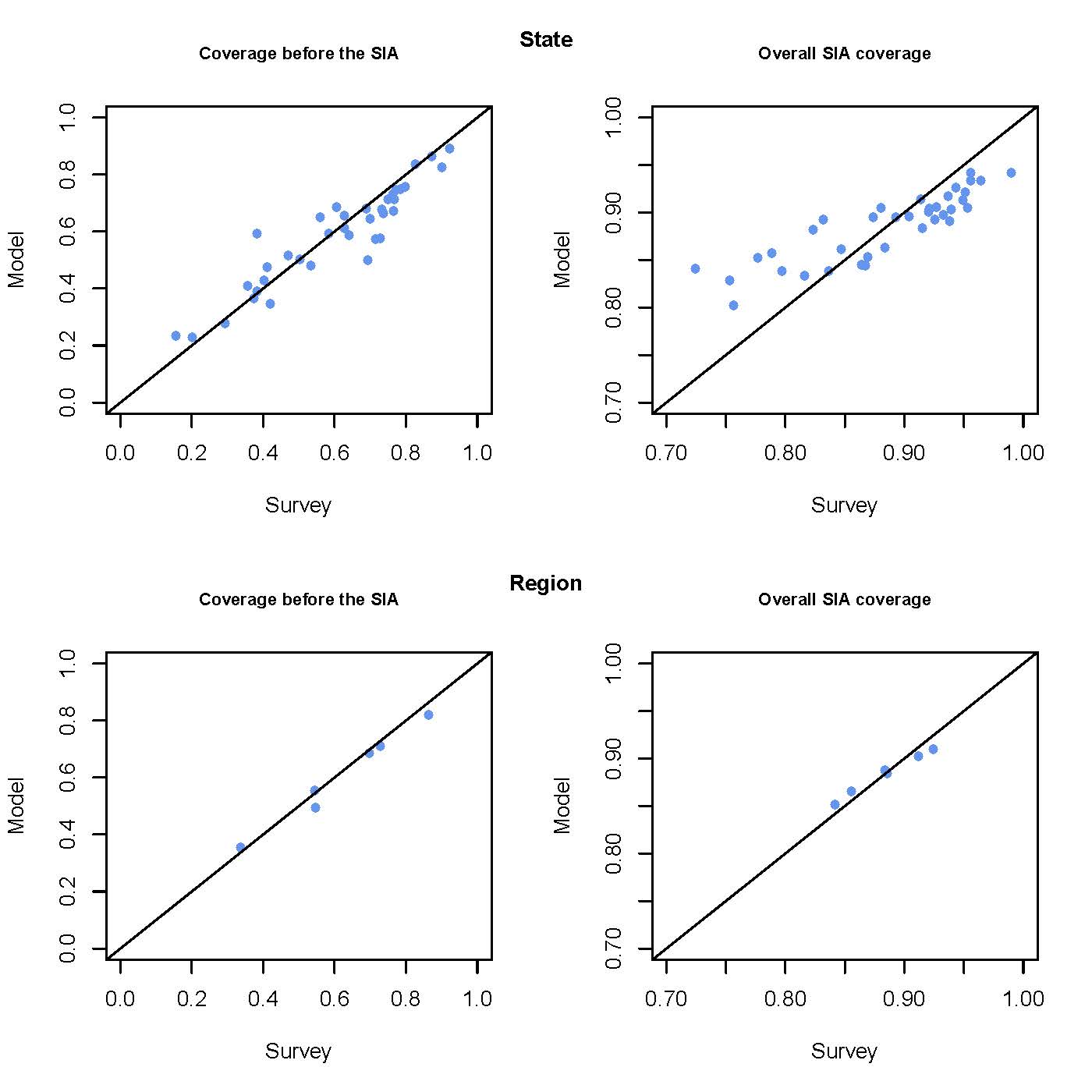
Figure 5: Direct survey vs predicted coverage estimates at the state and regional levels for *coverage before the SIA* and *overall SIA coverage*.


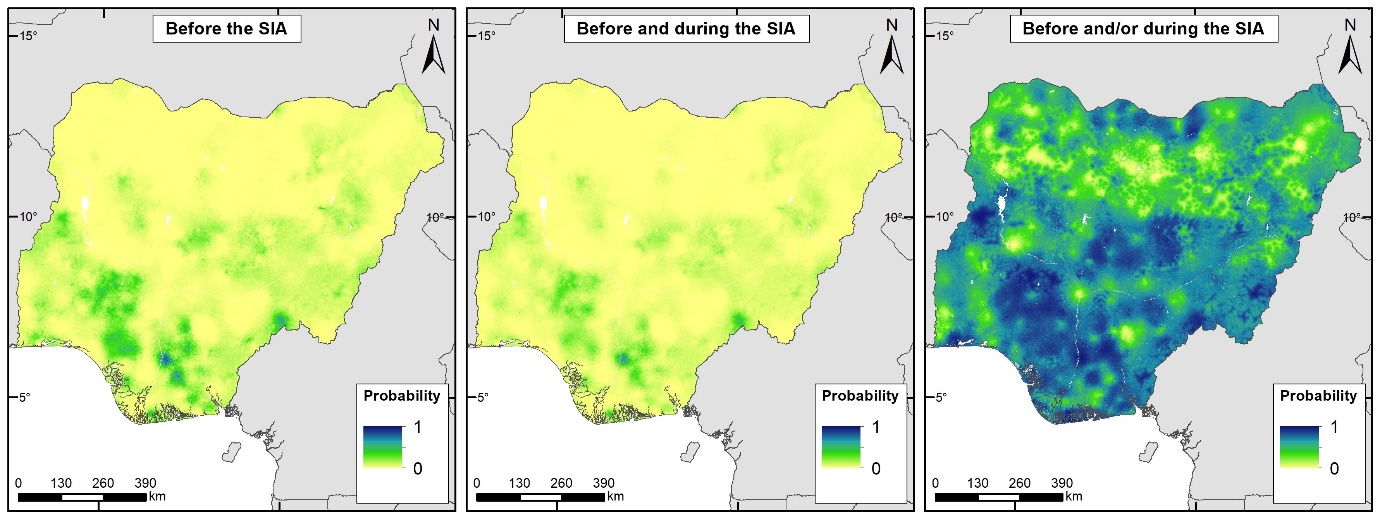


Figure 6: 1x1 km maps showing the probabilities of attaining 95% coverage among children aged 9-59 months for *coverage before the SIA*, *coverage before and during the SIA (i.e. coverage with at least two lifetime doses)* and *coverage before and/or during the SIA (i.e. coverage with at least one lifetime dose)*.


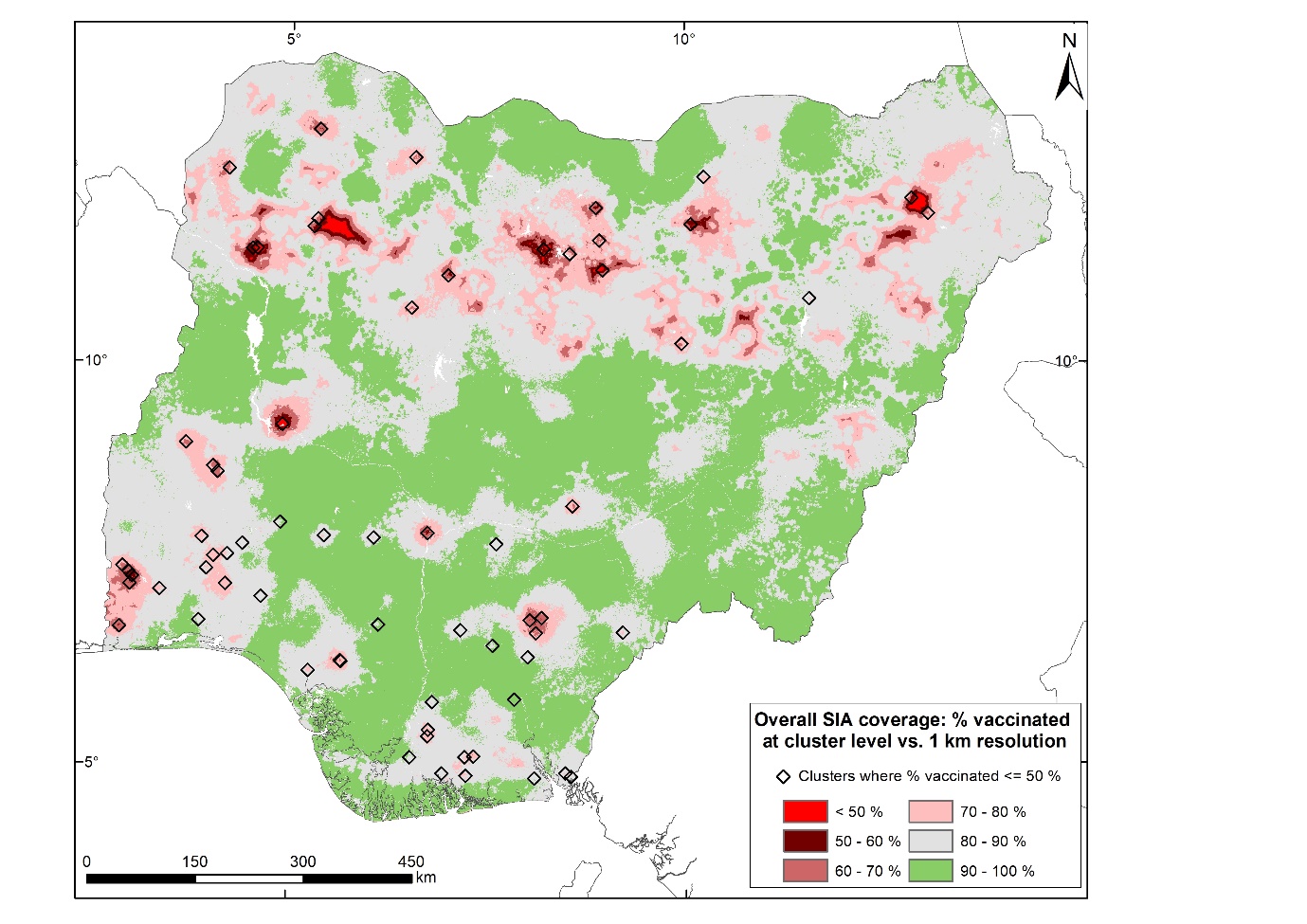


Figure 7: A map highlighting estimated low coverage areas (at 1x1 km) and low coverage clusters (i.e. clusters where <=50% coverage was observed) for *overall SIA* *coverage.* There were 66 of these low coverage clusters where at least 2 children were surveyed.


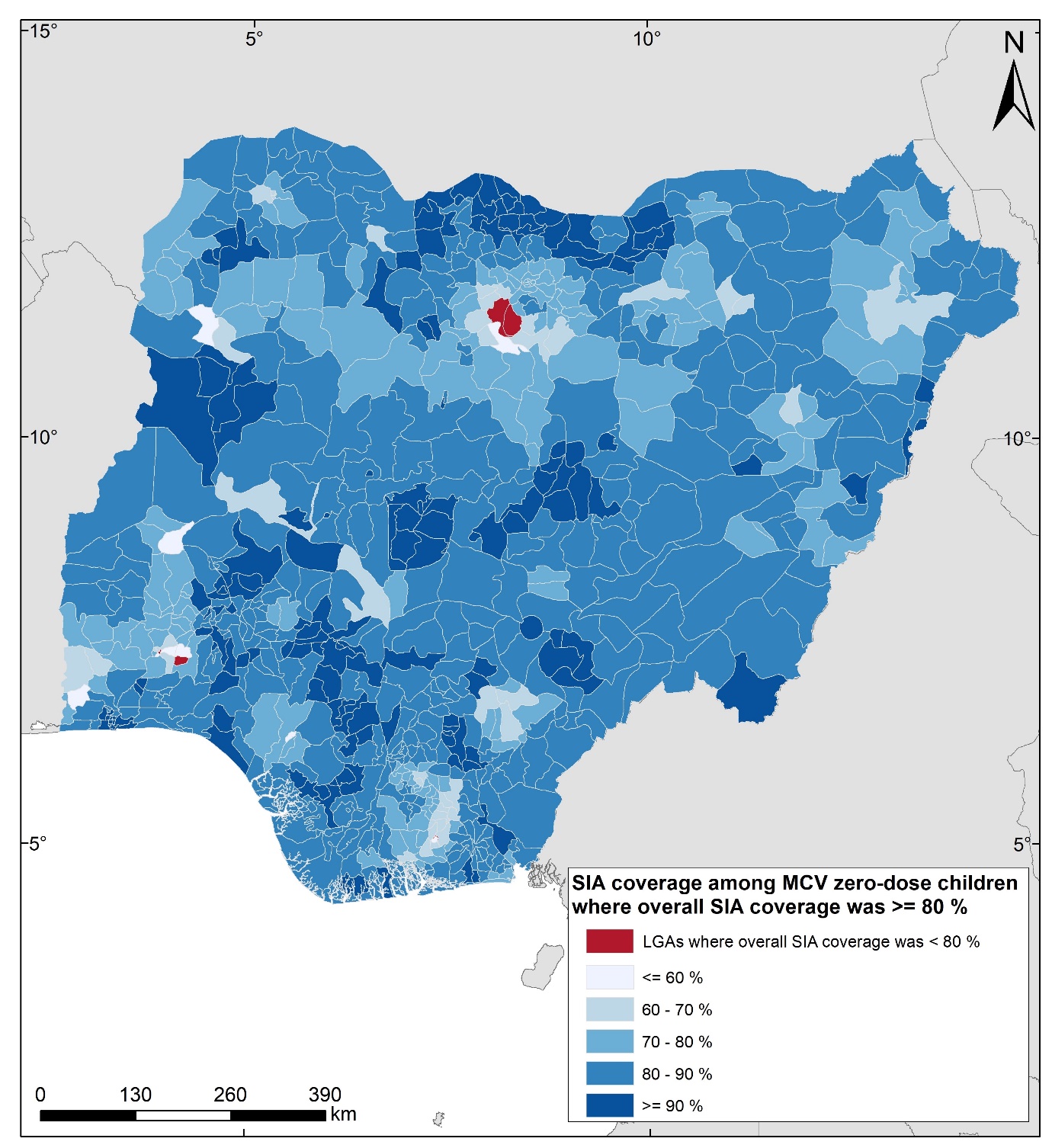


Figure 8: *SIA coverage among MCV zero-dose children* aged 9-59 months in LGAs/districts where predicted *overall SIA coverage* exceeded 80%.


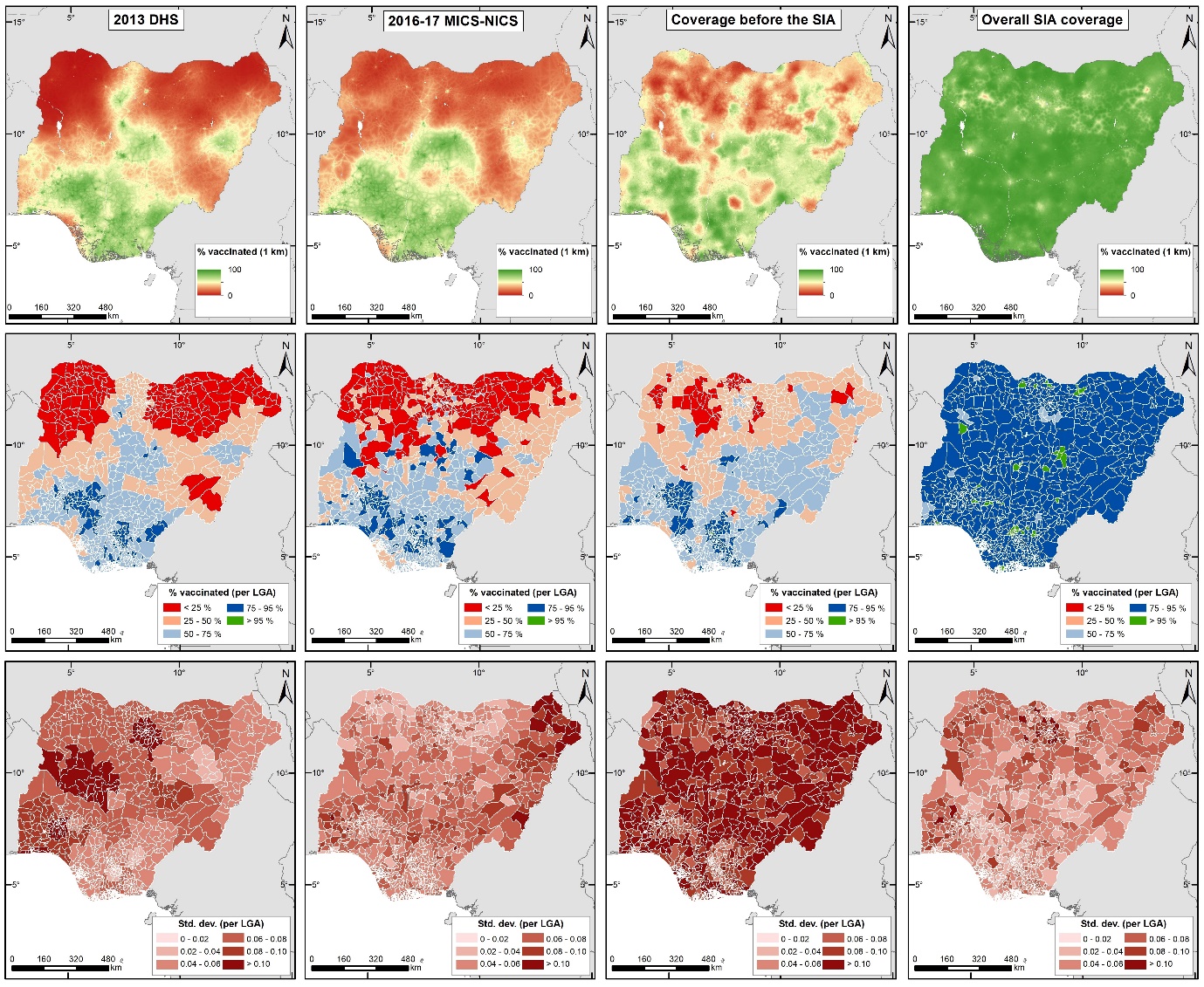


Figure 9: Comparison of predicted vaccination coverage at 1x1 km (top panel) and the LGA level (middle panel) using data from 2013 DHS, 2016-17 MICS-NICS and 2017-18 PCCS for children aged 9-59 months, 12-35 months and 9-59 months, respectively. LGAs attaining 95% coverage are shown in green in the middle panel. The bottom panel shows the uncertainties (i.e. standard deviations) associated with the LGA estimates.


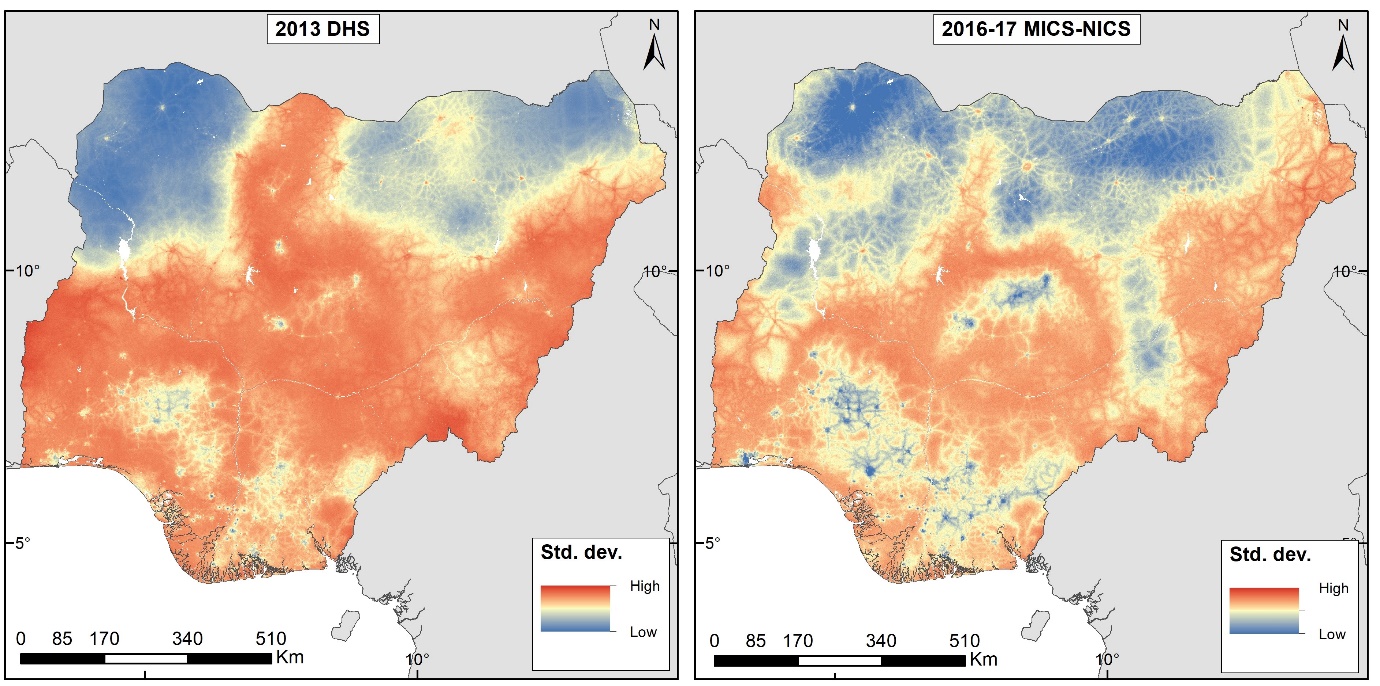


Figure 10: Uncertainty estimates (standard deviations) at 1x1 km for predicted measles vaccination coverage using 2013 DHS and 2016-17 MICS-NICS.


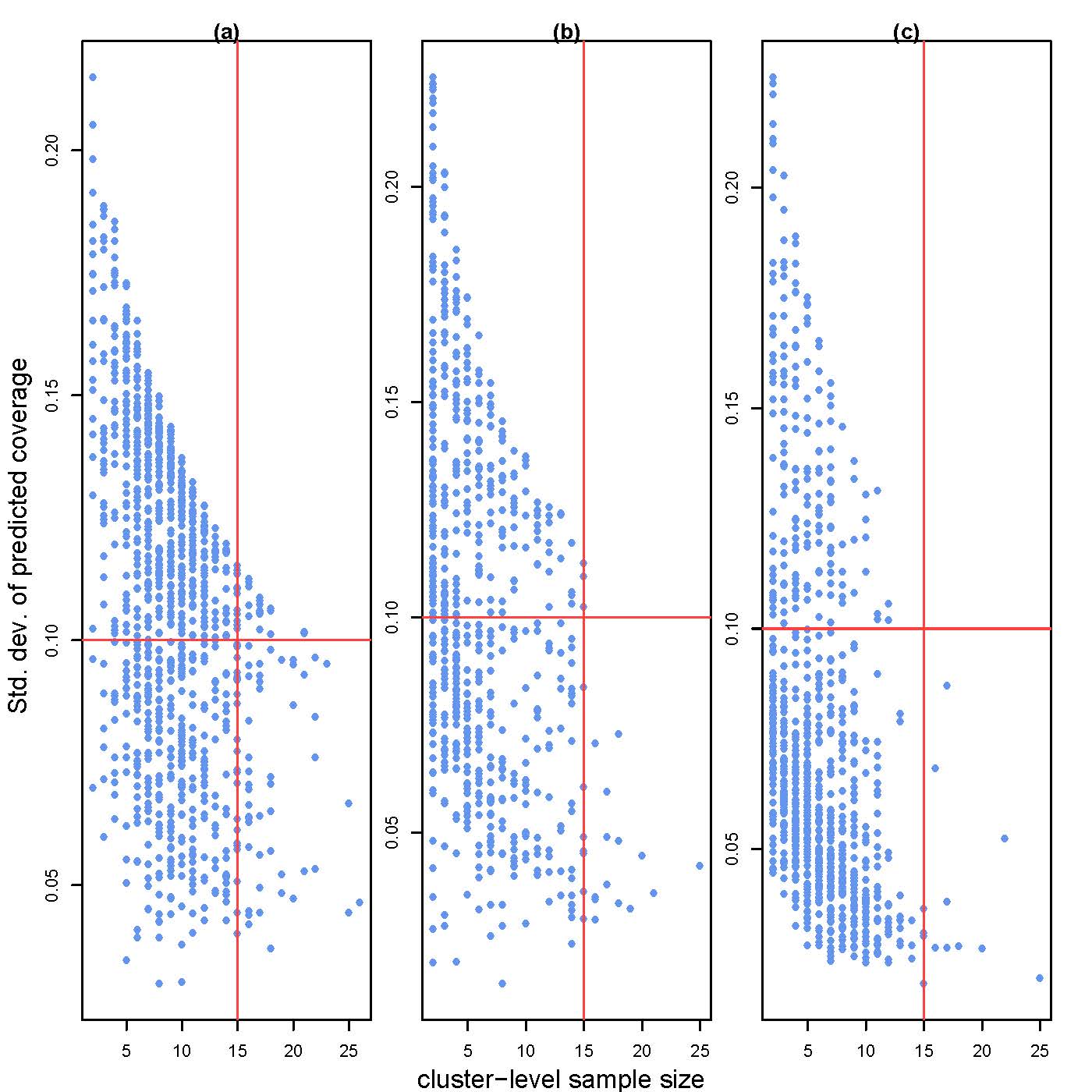


Figure 11: Plots of cluster-level sample size vs corresponding prediction standard deviation (error) for the three directly modelled indicators (a) *coverage before the SIA*, (b) *SIA coverage among zero-dose children* and (c) *SIA coverage among children vaccinated previously*. The red vertical and horizontal lines suggest that sample sizes > 15 are needed to achieve approximately 10% prediction error or less at the cluster level.

**Tables of LGA, state and regional modelled estimates of coverage for all five PCCS indicators of interest (uncertainty estimates are provided at the LGA level)**

Table 7: Modelled PCCS coverage estimates at the national and regional levels

|  | **Before SIA** | **Overall SIA coverage** | **SIA coverage among MCV zero dose children** | **Coverage before and during the SIA** | **Coverage before and/or during the SIA** |
| --- | --- | --- | --- | --- | --- |
| **National** | 57.7 | 88.0 | 83.3 | 54.0 | 91.8 |
| **Region** |  | | | | |
| north central | 55.6 | 91.0 | 88.3 | 53.0 | 93.6 |
| north east | 49.7 | 86.6 | 81.4 | 47.1 | 89.1 |
| north west | 35.6 | 85.2 | 81.4 | 33.9 | 86.9 |
| south east | 82.2 | 90.3 | 79.3 | 77.6 | 94.9 |
| south south | 68.8 | 88.8 | 85.0 | 63.0 | 94.5 |
| south west | 71.3 | 88.5 | 84.4 | 65.3 | 94.5 |

Table 8: Modelled PCCS coverage estimates at the state level

| **State** | **Coverage before SIA** | **Overall SIA coverage** | **SIA coverage among MCV zero dose children** | **Coverage before and during the SIA** | **Coverage before and/or during the SIA** |
| --- | --- | --- | --- | --- | --- |
| Abia | 73.2 | 84.5 | 67.0 | 68.8 | 88.9 |
| Adamawa | 50.5 | 89.2 | 84.5 | 48.2 | 91.5 |
| Akwa Ibom | 75.8 | 86.2 | 84.7 | 66.6 | 95.4 |
| Anambra | 89.3 | 94.3 | 83.3 | 86.0 | 97.6 |
| Bauchi | 41.2 | 83.4 | 79.0 | 39.0 | 85.6 |
| Bayelsa | 57.5 | 90.5 | 86.9 | 54.9 | 93.2 |
| Benue | 47.7 | 89.3 | 86.6 | 44.9 | 92.1 |
| Borno | 48.2 | 84.1 | 78.8 | 44.9 | 87.5 |
| Cross River | 67.3 | 89.5 | 86.2 | 62.3 | 94.6 |
| Delta | 68.2 | 92.7 | 88.7 | 65.0 | 95.9 |
| Ebonyi | 71.6 | 90.2 | 82.0 | 68.5 | 93.2 |
| Edo | 74.8 | 86.3 | 83.7 | 65.1 | 96.1 |
| Ekiti | 83.7 | 93.4 | 86.7 | 80.1 | 97.1 |
| Enugu | 82.8 | 91.4 | 87.3 | 77.4 | 96.8 |
| Gombe | 59.4 | 89.3 | 82.9 | 56.8 | 91.9 |
| Imo | 86.5 | 89.5 | 76.2 | 80.2 | 95.8 |
| Jigawa | 36.8 | 91.3 | 89.8 | 35.5 | 92.6 |
| Kaduna | 51.8 | 85.3 | 81.3 | 48.0 | 89.1 |
| Kano | 39.3 | 80.2 | 73.1 | 37.6 | 82.0 |
| Katsina | 23.7 | 90.5 | 89.3 | 22.7 | 91.4 |
| Kebbi | 28.0 | 83.9 | 80.5 | 27.0 | 84.9 |
| Kogi | 58.8 | 90.5 | 87.8 | 55.7 | 93.6 |
| Kwara | 64.5 | 90.3 | 87.8 | 60.4 | 94.5 |
| Lagos | 71.5 | 92.2 | 92.4 | 66.1 | 97.5 |
| Nassarawa | 61.2 | 90.6 | 86.7 | 58.2 | 93.6 |
| Niger | 42.9 | 89.6 | 87.0 | 41.3 | 91.3 |
| Ogun | 65.7 | 82.9 | 79.8 | 57.2 | 91.3 |
| Ondo | 68.8 | 91.8 | 88.6 | 64.8 | 95.8 |
| Osun | 75.2 | 85.7 | 83.4 | 66.5 | 94.4 |
| Oyo | 67.8 | 83.9 | 71.7 | 61.9 | 89.8 |
| Plateau | 66.4 | 93.4 | 89.9 | 64.2 | 95.6 |
| Rivers | 65.3 | 88.2 | 81.0 | 61.3 | 92.2 |
| Sokoto | 34.9 | 84.5 | 80.8 | 33.6 | 85.7 |
| Taraba | 57.7 | 89.8 | 85.0 | 54.9 | 92.6 |
| Yobe | 50.2 | 88.4 | 82.1 | 48.3 | 90.3 |
| Zamfara | 23.0 | 85.4 | 82.9 | 22.2 | 86.1 |
| Federal Capital Territory | 59.4 | 94.2 | 92.9 | 56.9 | 96.7 |

Table 9: Modelled PCCS coverage estimates and 95% credible intervals (in brackets) at the LGA/district level

| **State** | **LGA** | **Coverage before the SIA** | **Overall SIA coverage** | **SIA coverage among MCV zero-dose children** | **Coverage before and during the SIA** | **Coverage before and/or during the SIA** |
| --- | --- | --- | --- | --- | --- | --- |
| ABIA | ABA NORTH | 67.9 (49.3, 83.4) | 75.9 (61.3, 88.5) | 45.8 (24.8, 67.6) | 63.6 (45.1, 80.0) | 80.2 (65.9, 91.6) |
| ABIA | ABA SOUTH | 65.2 (47.4, 80.1) | 77.4 (63.0, 88.4) | 55.1 (30.7, 76.8) | 60.7 (42.9, 75.9) | 81.9 (67.7, 92.3) |
| ABIA | AROCHUKWU | 73.0 (55.8, 86.5) | 88.0 (78.9, 94.5) | 80.2 (59.4, 93.8) | 68.1 (51.1, 81.0) | 92.9 (83.9, 98.0) |
| ABIA | BENDE | 71.8 (50.2, 88.3) | 85.4 (72.1, 93.8) | 68.8 (39.1, 90.3) | 68.2 (47.5, 83.9) | 89.1 (76.6, 96.9) |
| ABIA | IKWUANO | 77.4 (59.4, 91.0) | 85.7 (72.1, 94.9) | 67.1 (34.1, 91.7) | 72.5 (55.1, 86.6) | 90.5 (76.7, 97.9) |
| ABIA | ISIALA NGWA NORTH | 84.8 (70.5, 94.3) | 89.3 (79.5, 96.1) | 67.3 (32.5, 92.0) | 80.5 (67.1, 90.8) | 93.7 (84.9, 98.6) |
| ABIA | ISIALA NGWA SOUTH | 80.7 (66.3, 91.8) | 88.2 (78.5, 95.1) | 68.2 (40.9, 89.9) | 76.7 (62.9, 87.4) | 92.1 (83.4, 97.9) |
| ABIA | ISUIKWUATO | 75.0 (51.0, 91.7) | 87.8 (73.4, 96.4) | 74.0 (40.2, 95.8) | 70.9 (48.0, 87.9) | 91.9 (77.7, 98.9) |
| ABIA | OBINGWA | 74.1 (59.6, 85.5) | 82.3 (72.7, 90.2) | 63.5 (41.0, 80.2) | 68.3 (55.1, 80.4) | 88.1 (78.3, 94.7) |
| ABIA | OHAFIA | 77.7 (57.8, 91.1) | 90.2 (78.6, 96.7) | 81.2 (49.1, 96.8) | 73.4 (54.1, 87.0) | 94.5 (84.0, 99.1) |
| ABIA | OSISIOMA | 68.6 (53.6, 81.4) | 80.6 (68.3, 89.9) | 60.2 (34.4, 81.1) | 64.3 (50.0, 77.2) | 85.0 (73.7, 93.5) |
| ABIA | UGWUNAGBO | 68.8 (51.1, 83.7) | 83.0 (70.6, 92.4) | 67.9 (39.9, 87.7) | 64.4 (47.5, 79.6) | 87.4 (74.8, 95.9) |
| ABIA | UKWA EAST | 75.8 (57.8, 90.2) | 86.7 (73.9, 94.6) | 77.1 (47.5, 94.8) | 69.8 (51.9, 84.4) | 92.8 (80.9, 98.6) |
| ABIA | UKWA WEST | 60.5 (38.6, 79.4) | 81.2 (65.1, 92.2) | 67.4 (36.5, 88.8) | 57.1 (35.8, 75.3) | 84.6 (67.9, 95.0) |
| ABIA | UMUNNEOCHI | 73.6 (55.5, 87.4) | 90.9 (80.7, 97.1) | 81.4 (54.6, 96.4) | 70.9 (53.4, 84.5) | 93.7 (84.0, 98.9) |
| ABIA | UMUAHIA NORTH | 75.6 (57.9, 89.6) | 84.1 (72.2, 92.9) | 63.5 (33.5, 85.8) | 70.7 (53.6, 83.7) | 89.0 (77.0, 96.4) |
| ABIA | UMUAHIA SOUTH | 80.4 (65.7, 91.4) | 87.0 (76.0, 94.6) | 65.1 (32.6, 89.3) | 76.1 (61.5, 87.0) | 91.4 (81.8, 97.4) |
| ADAMAWA | DEMSA | 39.7 (21.3, 61.8) | 83.7 (70.0, 93.4) | 79.8 (60.2, 92.8) | 37.4 (20.2, 58.2) | 86.1 (71.8, 95.1) |
| ADAMAWA | FUFORE | 49.0 (29.0, 67.7) | 87.8 (78.8, 94.3) | 83.4 (68.5, 93.2) | 46.4 (27.2, 64.3) | 90.4 (80.4, 96.3) |
| ADAMAWA | GANYE | 67.6 (36.6, 89.8) | 90.8 (76.6, 97.8) | 85.2 (56.1, 97.9) | 64.4 (34.9, 86.2) | 94.0 (80.5, 99.3) |
| ADAMAWA | GIREI | 46.0 (14.0, 83.2) | 87.4 (66.2, 97.2) | 83.9 (53.4, 97.9) | 43.4 (13.6, 77.8) | 90.0 (68.4, 98.8) |
| ADAMAWA | GOMBI | 45.0 (29.2, 63.4) | 91.9 (83.0, 97.1) | 91.5 (78.2, 98.1) | 42.4 (27.3, 59.8) | 94.4 (85.8, 98.8) |
| ADAMAWA | GUYUK | 74.8 (57.2, 87.5) | 90.9 (81.4, 97.0) | 77.3 (51.5, 94.0) | 73.1 (56.3, 85.8) | 92.6 (83.2, 98.1) |
| ADAMAWA | HONG | 29.4 (13.3, 49.8) | 88.3 (74.8, 95.8) | 86.6 (68.9, 96.2) | 27.9 (12.6, 47.4) | 89.7 (75.9, 96.9) |
| ADAMAWA | JADA | 72.1 (47.1, 91.1) | 89.4 (77.0, 96.9) | 77.5 (46.0, 95.9) | 69.3 (45.7, 88.3) | 92.2 (80.1, 98.8) |
| ADAMAWA | LAMURDE | 58.9 (27.0, 86.7) | 90.8 (76.5, 97.9) | 85.2 (59.2, 98.0) | 57.0 (26.5, 83.3) | 92.6 (77.7, 99.2) |
| ADAMAWA | MADAGALI | 55.8 (19.2, 88.6) | 90.9 (73.8, 98.3) | 87.8 (59.4, 99.0) | 53.1 (18.6, 85.5) | 93.5 (77.5, 99.5) |
| ADAMAWA | MAIHA | 25.8 (08.3, 50.4) | 93.6 (82.5, 98.5) | 93.0 (78.6, 99.0) | 24.9 (08.1, 49.0) | 94.5 (84.1, 99.1) |
| ADAMAWA | MAYO-BELWA | 53.8 (29.7, 77.0) | 84.0 (67.4, 94.2) | 74.1 (47.2, 92.3) | 51.5 (28.6, 73.5) | 86.3 (68.7, 96.5) |
| ADAMAWA | MICHIKA | 57.1 (32.6, 80.1) | 93.5 (83.8, 98.3) | 91.7 (74.1, 98.8) | 55.2 (32.2, 77.6) | 95.5 (86.0, 99.4) |
| ADAMAWA | MUBI NORTH | 25.2 (08.9, 48.3) | 90.5 (76.4, 97.5) | 89.4 (72.5, 97.8) | 24.3 (08.8, 46.8) | 91.3 (77.2, 98.2) |
| ADAMAWA | MUBI SOUTH | 20.6 (03.6, 51.4) | 91.7 (71.0, 99.1) | 91.2 (68.2, 99.3) | 19.9 (03.6, 50.2) | 92.4 (72.4, 99.4) |
| ADAMAWA | NUMAN | 51.4 (16.7, 84.3) | 88.9 (70.7, 97.8) | 84.2 (53.6, 98.4) | 49.3 (15.7, 81.1) | 91.0 (73.1, 99.1) |
| ADAMAWA | SHELLENG | 66.9 (40.8, 88.0) | 90.5 (78.2, 97.2) | 82.3 (55.8, 96.6) | 64.7 (39.8, 84.8) | 92.7 (80.2, 98.7) |
| ADAMAWA | SONG | 40.8 (15.5, 68.1) | 90.0 (76.5, 97.2) | 87.9 (68.2, 97.6) | 39.0 (15.0, 66.2) | 91.8 (77.2, 98.5) |
| ADAMAWA | TOUNGO | 68.4 (47.7, 85.5) | 92.0 (82.3, 97.4) | 89.5 (69.4, 98.2) | 64.6 (46.2, 81.4) | 95.7 (87.4, 99.3) |
| ADAMAWA | YOLA NORTH | 49.7 (28.3, 72.0) | 86.2 (71.9, 95.4) | 82.9 (58.7, 95.8) | 46.1 (26.5, 67.2) | 89.8 (76.0, 97.6) |
| ADAMAWA | YOLA SOUTH | 54.6 (31.7, 76.2) | 86.5 (72.6, 95.1) | 81.9 (58.1, 94.7) | 51.2 (30.1, 71.6) | 90.0 (75.7, 97.5) |
| AKWA IBOM | ABAK | 81.2 (61.1, 93.7) | 80.1 (62.0, 91.9) | 82.6 (54.3, 96.8) | 65.7 (44.1, 81.9) | 95.6 (87.8, 99.3) |
| AKWA IBOM | EASTERN OBOLO | 44.8 (23.8, 67.2) | 93.0 (82.9, 98.6) | 87.0 (72.3, 92.4) | 43.4 (23.1, 65.7) | 94.4 (84.2, 99.3) |
| AKWA IBOM | EKET | 79.4 (60.9, 91.5) | 91.4 (82.4, 97.3) | 82.9 (57.3, 95.6) | 75.2 (58.4, 87.2) | 95.6 (87.4, 99.3) |
| AKWA IBOM | ESIT EKET | 77.8 (55.9, 92.1) | 89.6 (77.2, 97.0) | 79.6 (44.8, 96.9) | 73.4 (51.1, 88.3) | 94.0 (82.9, 99.2) |
| AKWA IBOM | ESSIEN UDIM | 84.0 (71.0, 92.9) | 81.5 (69.5, 90.5) | 82.3 (55.5, 96.4) | 69.4 (55.5, 80.7) | 96.1 (90.2, 99.2) |
| AKWA IBOM | ETIM EKPO | 86.0 (71.1, 95.3) | 85.7 (73.4, 94.2) | 81.3 (54.8, 96.0) | 75.3 (59.7, 87.3) | 96.4 (90.5, 99.4) |
| AKWA IBOM | ETINAN | 75.1 (51.6, 91.1) | 88.8 (75.9, 96.6) | 87.3 (62.8, 98.0) | 68.2 (46.2, 84.1) | 95.7 (86.0, 99.4) |
| AKWA IBOM | IBENO | 68.5 (48.9, 84.9) | 89.0 (77.7, 96.1) | 79.3 (52.9, 93.8) | 64.8 (46.4, 80.9) | 92.6 (82.2, 98.4) |
| AKWA IBOM | IBESIKPO ASUTAN | 75.0 (55.9, 89.0) | 83.2 (69.5, 92.5) | 86.0 (64.0, 96.7) | 63.1 (45.8, 79.6) | 95.2 (87.2, 99.0) |
| AKWA IBOM | IBIONO IBOM | 73.4 (47.9, 90.9) | 85.4 (66.1, 95.5) | 88.7 (64.4, 98.5) | 62.7 (40.4, 82.5) | 96.1 (87.1, 99.5) |
| AKWA IBOM | IKA | 86.7 (73.1, 95.0) | 86.2 (75.1, 93.8) | 77.5 (46.7, 95.4) | 77.0 (62.3, 87.9) | 95.9 (89.4, 99.3) |
| AKWA IBOM | IKONO | 80.4 (62.2, 92.1) | 84.4 (70.0, 93.3) | 88.0 (66.2, 98.1) | 68.0 (51.3, 81.6) | 96.7 (90.8, 99.5) |
| AKWA IBOM | IKOT ABASI | 56.8 (33.1, 77.5) | 91.9 (80.8, 97.9) | 88.8 (68.6, 98.2) | 54.6 (32.9, 73.6) | 94.1 (83.0, 99.2) |
| AKWA IBOM | IKOT EKPENE | 84.4 (70.0, 94.2) | 79.1 (61.5, 90.3) | 85.6 (57.3, 98.3) | 66.6 (49.5, 80.5) | 96.9 (89.9, 99.6) |
| AKWA IBOM | INI | 76.4 (57.3, 90.3) | 88.2 (77.7, 95.3) | 81.7 (56.7, 96.0) | 70.5 (52.3, 84.4) | 94.1 (85.0, 98.8) |
| AKWA IBOM | ITU | 68.8 (41.7, 89.9) | 84.5 (66.6, 94.8) | 89.2 (67.7, 98.5) | 57.4 (33.6, 79.4) | 95.8 (86.9, 99.6) |
| AKWA IBOM | MBO | 73.9 (51.3, 90.4) | 85.8 (72.3, 94.2) | 76.4 (47.1, 93.7) | 67.4 (47.1, 84.0) | 92.3 (80.8, 98.4) |
| AKWA IBOM | MKPAT ENIN | 58.2 (38.7, 76.0) | 92.4 (83.9, 97.4) | 90.6 (75.4, 98.0) | 55.3 (35.8, 72.5) | 95.3 (87.6, 99.0) |
| AKWA IBOM | NSIT ATAI | 83.6 (66.6, 93.9) | 88.4 (75.3, 95.8) | 88.0 (62.4, 98.7) | 74.8 (58.4, 87.1) | 97.2 (90.1, 99.7) |
| AKWA IBOM | NSIT IBOM | 76.3 (55.8, 90.0) | 85.3 (72.2, 93.9) | 84.9 (61.6, 96.5) | 66.5 (47.9, 81.1) | 95.1 (86.9, 99.0) |
| AKWA IBOM | NSIT UBIUM | 82.7 (66.7, 93.2) | 91.5 (82.1, 97.3) | 87.7 (63.9, 98.4) | 77.2 (61.5, 88.6) | 97.0 (90.1, 99.7) |
| AKWA IBOM | OBOT AKARA | 80.8 (60.9, 93.8) | 85.0 (69.8, 94.2) | 79.3 (47.2, 96.7) | 71.1 (51.6, 86.3) | 94.7 (84.7, 99.3) |
| AKWA IBOM | OKOBO | 82.5 (67.4, 92.8) | 88.1 (77.8, 95.1) | 86.3 (61.7, 98.0) | 73.9 (59.6, 85.6) | 96.7 (90.3, 99.6) |
| AKWA IBOM | ONNA | 63.3 (40.7, 81.6) | 93.3 (84.6, 98.1) | 91.2 (75.8, 98.8) | 60.7 (39.3, 78.6) | 95.8 (88.1, 99.4) |
| AKWA IBOM | ORON | 80.8 (62.6, 93.5) | 87.0 (70.2, 96.1) | 85.8 (56.2, 98.0) | 71.6 (51.7, 86.8) | 96.2 (87.7, 99.5) |
| AKWA IBOM | ORUK ANAM | 74.5 (54.2, 88.4) | 89.1 (78.8, 95.9) | 84.8 (61.6, 97.0) | 68.8 (49.7, 83.0) | 94.9 (86.4, 99.1) |
| AKWA IBOM | UDUNG UKO | 80.4 (62.6, 93.3) | 88.3 (75.9, 96.0) | 82.5 (52.8, 97.2) | 73.5 (55.8, 87.6) | 95.3 (86.7, 99.4) |
| AKWA IBOM | UKANAFUN | 83.5 (65.2, 94.5) | 87.2 (74.1, 95.4) | 83.2 (54.3, 97.2) | 74.5 (57.2, 88.2) | 96.2 (88.7, 99.5) |
| AKWA IBOM | URUAN | 74.0 (53.9, 89.6) | 83.9 (67.8, 93.5) | 88.5 (63.0, 98.7) | 61.9 (42.1, 77.8) | 96.0 (87.0, 99.6) |
| AKWA IBOM | URUE OFFONG ORUKO | 83.5 (67.9, 93.3) | 88.9 (78.3, 95.7) | 81.5 (51.9, 96.4) | 76.6 (61.7, 88.0) | 95.8 (89.0, 99.3) |
| AKWA IBOM | UYO | 74.9 (54.4, 89.5) | 77.2 (59.4, 90.0) | 81.4 (55.7, 95.7) | 58.0 (38.6, 76.4) | 94.1 (84.8, 98.8) |
| ANAMBRA | AGUATA | 90.4 (79.3, 96.8) | 92.6 (85.0, 97.5) | 73.8 (39.6, 95.4) | 86.5 (75.6, 93.7) | 96.6 (90.9, 99.5) |
| ANAMBRA | ANAMBRA EAST | 90.2 (80.4, 96.2) | 95.4 (90.7, 98.4) | 89.6 (67.5, 98.5) | 87.1 (77.2, 93.5) | 98.6 (95.6, 99.8) |
| ANAMBRA | ANAMBRA WEST | 81.2 (66.9, 92.5) | 92.0 (83.4, 97.1) | 90.5 (70.1, 98.6) | 75.8 (62.2, 87.2) | 97.5 (91.8, 99.7) |
| ANAMBRA | ANAOCHA | 91.8 (81.4, 97.5) | 94.9 (89.4, 98.5) | 82.5 (50.0, 97.9) | 88.7 (78.0, 95.5) | 98.0 (93.7, 99.8) |
| ANAMBRA | AWKA NORTH | 81.6 (66.8, 91.9) | 95.1 (89.0, 98.5) | 89.1 (69.0, 98.1) | 79.6 (65.5, 90.0) | 97.1 (91.9, 99.6) |
| ANAMBRA | AWKA SOUTH | 82.8 (67.8, 92.8) | 95.2 (89.1, 98.6) | 87.8 (68.5, 97.1) | 80.8 (66.4, 91.2) | 97.1 (91.6, 99.5) |
| ANAMBRA | AYAMELUM | 79.8 (58.6, 94.8) | 91.3 (79.4, 97.5) | 84.1 (54.3, 98.1) | 75.4 (55.1, 90.6) | 95.6 (84.4, 99.6) |
| ANAMBRA | DUNUKOFIA | 90.8 (80.7, 96.7) | 95.7 (89.8, 98.9) | 87.8 (59.7, 98.5) | 88.1 (77.6, 94.9) | 98.4 (94.6, 99.8) |
| ANAMBRA | EKWUSIGO | 94.7 (84.5, 99.0) | 95.1 (86.7, 99.0) | 81.6 (41.8, 98.5) | 91.1 (79.4, 97.3) | 98.6 (94.6, 99.9) |
| ANAMBRA | IDEMILI NORTH | 95.5 (88.8, 98.9) | 96.3 (91.2, 99.0) | 85.0 (52.9, 98.7) | 92.8 (85.2, 97.2) | 99.0 (96.5, 99.9) |
| ANAMBRA | IDEMILI SOUTH | 95.2 (88.2, 98.8) | 96.2 (91.0, 98.9) | 83.8 (52.9, 98.3) | 92.5 (85.1, 97.1) | 98.9 (96.0, 99.9) |
| ANAMBRA | IHIALA | 84.4 (67.3, 94.7) | 89.6 (79.0, 96.2) | 79.3 (47.9, 96.4) | 78.4 (62.1, 89.8) | 95.6 (87.7, 99.4) |
| ANAMBRA | NJIKOKA | 90.3 (80.5, 96.5) | 96.0 (90.4, 98.9) | 87.7 (63.4, 98.4) | 88.0 (77.5, 94.7) | 98.3 (94.1, 99.8) |
| ANAMBRA | NNEWI NORTH | 94.7 (84.5, 99.0) | 95.0 (86.7, 98.8) | 80.0 (37.0, 98.4) | 91.2 (81.3, 97.0) | 98.5 (94.4, 99.9) |
| ANAMBRA | NNEWI SOUTH | 90.9 (77.7, 97.4) | 93.7 (86.1, 97.9) | 77.8 (42.9, 96.3) | 87.4 (74.9, 94.7) | 97.2 (91.2, 99.6) |
| ANAMBRA | OGBARU | 83.6 (66.8, 95.0) | 93.5 (85.2, 98.1) | 86.0 (57.5, 98.5) | 80.0 (63.3, 91.8) | 97.1 (90.3, 99.7) |
| ANAMBRA | ONITSHA NORTH | 94.8 (85.3, 98.8) | 96.8 (90.6, 99.4) | 87.2 (48.1, 99.2) | 92.6 (82.1, 97.7) | 99.0 (95.5, 100.0) |
| ANAMBRA | ONITSHA SOUTH | 93.9 (82.1, 99.1) | 96.4 (88.0, 99.5) | 86.8 (46.9, 99.3) | 91.5 (79.0, 98.0) | 98.8 (93.8, 100.0) |
| ANAMBRA | ORUMBA NORTH | 86.7 (74.0, 94.7) | 94.4 (88.2, 98.2) | 83.5 (56.3, 97.4) | 84.1 (71.6, 92.2) | 97.1 (91.6, 99.6) |
| ANAMBRA | ORUMBA SOUTH | 84.0 (70.0, 92.9) | 93.1 (85.7, 97.8) | 79.7 (50.9, 96.1) | 81.4 (67.6, 90.6) | 95.7 (89.0, 99.3) |
| ANAMBRA | OYI | 94.3 (86.8, 98.3) | 96.4 (91.2, 99.0) | 86.7 (57.5, 98.5) | 91.8 (83.9, 96.7) | 98.9 (96.2, 99.9) |
| BAUCHI | ALKALERI | 50.2 (27.2, 72.7) | 88.6 (77.4, 95.2) | 84.5 (66.1, 94.6) | 48.0 (26.4, 69.4) | 90.8 (79.8, 97.3) |
| BAUCHI | BAUCHI | 45.2 (23.7, 68.7) | 81.1 (66.5, 91.0) | 77.0 (53.8, 92.2) | 41.1 (22.5, 63.9) | 85.3 (70.6, 95.2) |
| BAUCHI | BOGORO | 57.6 (30.8, 81.8) | 88.7 (73.5, 97.2) | 81.7 (51.9, 96.8) | 55.9 (29.3, 78.8) | 90.4 (75.1, 98.4) |
| BAUCHI | DAMBAM | 45.5 (23.8, 68.7) | 85.3 (71.0, 94.6) | 79.6 (56.7, 94.2) | 43.2 (22.8, 64.7) | 87.5 (72.8, 96.5) |
| BAUCHI | DARAZO | 57.0 (37.2, 75.0) | 87.2 (76.7, 94.6) | 80.1 (62.1, 92.8) | 54.7 (35.8, 72.1) | 89.6 (79.0, 96.7) |
| BAUCHI | DASS | 63.8 (37.5, 85.0) | 94.3 (85.4, 98.8) | 92.2 (73.8, 99.1) | 61.8 (36.2, 82.4) | 96.3 (87.9, 99.6) |
| BAUCHI | GAMAWA | 35.9 (14.9, 59.2) | 81.9 (65.3, 92.2) | 78.5 (54.6, 92.4) | 33.4 (14.0, 54.6) | 84.4 (68.0, 94.6) |
| BAUCHI | GANJUWA | 29.0 (11.4, 51.3) | 81.4 (65.4, 91.4) | 78.2 (58.1, 90.9) | 27.4 (10.9, 47.8) | 83.1 (67.1, 92.9) |
| BAUCHI | GIADE | 34.0 (15.0, 56.0) | 79.8 (63.9, 91.3) | 76.4 (56.7, 91.0) | 31.3 (14.2, 52.6) | 82.5 (66.9, 93.8) |
| BAUCHI | ITAS GADAW | 37.9 (21.7, 55.6) | 75.9 (61.9, 87.0) | 67.7 (48.3, 83.5) | 36.6 (20.9, 54.3) | 77.3 (62.7, 88.2) |
| BAUCHI | JAMAARE | 49.9 (24.9, 72.8) | 84.9 (68.0, 94.8) | 78.7 (51.6, 94.3) | 47.8 (23.9, 70.0) | 87.0 (69.5, 96.4) |
| BAUCHI | KATAGUM | 33.2 (16.4, 51.5) | 76.6 (61.4, 88.1) | 71.5 (52.1, 86.3) | 30.9 (15.3, 48.2) | 78.8 (63.4, 90.1) |
| BAUCHI | KIRFI | 25.8 (08.1, 49.1) | 85.1 (68.7, 95.8) | 82.5 (62.0, 95.8) | 24.7 (07.5, 47.2) | 86.3 (69.8, 96.8) |
| BAUCHI | MISAU | 49.1 (31.0, 67.2) | 82.9 (70.7, 92.1) | 75.0 (55.7, 89.3) | 47.0 (30.3, 64.6) | 85.1 (72.2, 93.6) |
| BAUCHI | NINGI | 26.0 (09.2, 48.8) | 78.6 (61.1, 90.0) | 75.1 (53.3, 89.4) | 25.0 (09.1, 46.6) | 79.6 (62.4, 91.1) |
| BAUCHI | SHIRA | 35.8 (17.6, 57.0) | 84.4 (70.7, 93.9) | 82.8 (63.6, 94.2) | 32.8 (15.7, 53.3) | 87.3 (73.5, 96.2) |
| BAUCHI | TAFAWA BALEWA | 65.7 (45.9, 81.5) | 93.2 (85.9, 97.3) | 89.4 (73.1, 97.1) | 63.7 (44.7, 79.2) | 95.1 (88.2, 98.7) |
| BAUCHI | TORO | 40.2 (22.7, 59.6) | 88.3 (77.4, 95.2) | 85.3 (69.6, 94.6) | 38.9 (22.2, 57.3) | 89.6 (78.3, 96.2) |
| BAUCHI | WARJI | 33.7 (10.2, 65.1) | 84.4 (62.3, 95.8) | 80.8 (52.1, 95.9) | 32.4 (09.9, 61.9) | 85.6 (63.2, 96.9) |
| BAUCHI | ZAKI | 32.2 (13.0, 57.8) | 79.6 (60.0, 92.7) | 76.3 (50.1, 92.3) | 29.7 (11.9, 53.3) | 82.1 (61.5, 94.5) |
| BAYELSA | BRASS | 71.1 (52.0, 86.4) | 93.8 (85.5, 98.3) | 88.0 (71.1, 94.5) | 68.7 (50.4, 83.7) | 96.2 (89.9, 99.3) |
| BAYELSA | EKEREMOR | 56.5 (32.5, 80.8) | 90.5 (78.8, 96.9) | 86.5 (67.4, 97.0) | 54.0 (30.4, 76.5) | 92.9 (81.1, 98.5) |
| BAYELSA | KOLOKUMA/OPOKUMA | 62.8 (47.3, 77.1) | 92.3 (84.4, 96.9) | 89.7 (73.9, 97.7) | 60.2 (45.2, 74.0) | 94.9 (87.2, 98.8) |
| BAYELSA | NEMBE | 79.0 (64.3, 91.4) | 95.7 (89.2, 99.1) | 91.7 (72.4, 99.1) | 77.0 (62.9, 89.5) | 97.7 (92.0, 99.8) |
| BAYELSA | OGBIA | 61.0 (45.8, 74.9) | 88.5 (79.1, 94.9) | 83.4 (64.3, 94.4) | 57.8 (43.4, 71.1) | 91.7 (82.7, 97.3) |
| BAYELSA | SAGBAMA | 63.6 (47.5, 77.8) | 91.4 (83.1, 96.6) | 86.5 (67.9, 96.5) | 61.3 (45.5, 74.9) | 93.7 (85.2, 98.3) |
| BAYELSA | SOUTHERN IJAW | 41.9 (23.6, 62.0) | 90.5 (82.0, 96.0) | 88.0 (75.1, 95.5) | 40.3 (22.5, 59.4) | 92.1 (82.9, 97.3) |
| BAYELSA | YENAGOA | 52.0 (35.5, 68.2) | 87.4 (77.1, 94.6) | 85.4 (68.8, 95.0) | 48.0 (32.1, 63.0) | 91.4 (81.6, 97.2) |
| BENUE | ADO | 29.9 (15.4, 49.4) | 74.2 (58.7, 86.1) | 69.3 (50.1, 84.2) | 27.8 (14.2, 44.9) | 76.2 (60.8, 88.0) |
| BENUE | AGATU | 52.5 (20.7, 81.8) | 89.2 (71.7, 97.6) | 84.8 (54.3, 97.7) | 50.3 (20.1, 77.9) | 91.4 (73.2, 98.8) |
| BENUE | APA | 63.8 (39.9, 83.6) | 90.7 (78.7, 97.2) | 88.7 (66.3, 98.4) | 59.8 (37.6, 80.0) | 94.8 (84.5, 99.3) |
| BENUE | BURUKU | 37.7 (17.8, 59.9) | 93.3 (82.6, 98.4) | 92.5 (77.3, 98.7) | 36.4 (17.4, 57.2) | 94.6 (83.6, 99.1) |
| BENUE | GBOKO | 38.7 (17.3, 61.2) | 95.0 (85.7, 98.8) | 94.9 (82.1, 99.3) | 37.4 (16.7, 59.2) | 96.3 (87.0, 99.5) |
| BENUE | GUMA | 42.6 (21.6, 67.5) | 91.6 (80.0, 97.2) | 89.8 (71.1, 97.8) | 40.8 (20.5, 64.6) | 93.4 (81.3, 98.8) |
| BENUE | GWER | 55.9 (35.4, 75.2) | 90.9 (81.3, 96.8) | 88.7 (72.2, 97.8) | 52.5 (33.4, 70.2) | 94.3 (85.6, 98.8) |
| BENUE | GWER WEST | 58.3 (24.6, 86.9) | 90.6 (75.3, 97.9) | 89.8 (64.5, 98.8) | 54.2 (23.4, 81.8) | 94.7 (80.3, 99.5) |
| BENUE | KATSINA - ALA | 51.0 (25.3, 77.5) | 90.4 (77.4, 97.4) | 87.0 (62.5, 97.7) | 48.8 (24.5, 74.0) | 92.6 (80.0, 98.8) |
| BENUE | KONSHISHA | 47.0 (29.0, 66.4) | 89.8 (80.1, 95.9) | 87.3 (71.7, 96.1) | 44.4 (27.1, 62.5) | 92.4 (82.9, 97.8) |
| BENUE | KWANDE | 66.1 (38.9, 88.1) | 89.0 (76.3, 96.2) | 87.1 (65.3, 97.5) | 60.5 (34.3, 81.5) | 94.6 (83.1, 99.2) |
| BENUE | LOGO | 33.0 (11.9, 58.3) | 89.0 (73.0, 97.1) | 86.8 (65.5, 97.1) | 31.9 (11.2, 56.2) | 90.1 (74.2, 97.9) |
| BENUE | MAKURDI | 49.2 (19.2, 76.7) | 93.0 (80.6, 98.7) | 92.8 (73.6, 99.3) | 46.6 (18.2, 72.7) | 95.6 (84.2, 99.6) |
| BENUE | OBI | 56.1 (29.5, 82.6) | 80.6 (60.5, 93.6) | 72.3 (36.0, 94.6) | 50.8 (26.6, 75.6) | 85.9 (66.0, 97.1) |
| BENUE | OGBADIBO | 47.0 (29.9, 65.5) | 88.8 (76.6, 95.7) | 86.2 (67.0, 96.0) | 44.1 (27.6, 62.2) | 91.7 (80.4, 97.4) |
| BENUE | OHIMINI | 41.3 (19.3, 65.0) | 91.2 (77.2, 97.8) | 90.5 (70.3, 98.7) | 39.0 (18.1, 60.8) | 93.5 (79.0, 99.1) |
| BENUE | OJU | 59.9 (37.3, 79.5) | 77.7 (62.3, 89.3) | 62.4 (33.3, 85.9) | 55.0 (34.8, 73.5) | 82.6 (66.7, 93.6) |
| BENUE | OKPOKWU | 19.0 (06.5, 37.2) | 90.8 (79.1, 97.1) | 90.6 (77.2, 97.6) | 18.1 (06.3, 35.7) | 91.8 (80.1, 98.0) |
| BENUE | OTUKPO | 56.9 (35.1, 78.0) | 88.4 (76.1, 95.9) | 87.7 (67.8, 97.6) | 52.0 (32.6, 71.5) | 93.3 (82.0, 98.7) |
| BENUE | TARKA | 43.1 (17.6, 70.2) | 95.4 (86.5, 99.2) | 95.3 (82.4, 99.6) | 41.7 (17.2, 68.7) | 96.8 (87.8, 99.7) |
| BENUE | UKUM | 45.1 (15.1, 75.2) | 86.9 (68.2, 96.8) | 82.3 (52.2, 96.6) | 43.3 (14.6, 72.6) | 88.7 (68.9, 98.0) |
| BENUE | USHONGO | 44.5 (23.6, 64.9) | 91.7 (81.0, 97.3) | 90.1 (73.5, 98.0) | 42.3 (22.5, 61.5) | 93.9 (82.9, 98.7) |
| BENUE | VANDEIKYA | 60.3 (39.3, 79.0) | 88.2 (75.4, 95.8) | 83.5 (59.6, 95.9) | 56.6 (37.3, 74.7) | 91.9 (79.1, 97.9) |
| BORNO | ABADAM | 42.9 (12.5, 78.7) | 89.5 (73.6, 97.6) | 86.5 (64.0, 98.0) | 41.1 (11.9, 75.3) | 91.3 (74.8, 98.8) |
| BORNO | ASKIRA/UBA | 45.0 (28.4, 61.7) | 85.2 (73.0, 93.4) | 81.9 (63.3, 93.2) | 42.3 (26.8, 57.8) | 88.0 (77.0, 95.5) |
| BORNO | BAMA | 43.3 (13.9, 77.2) | 86.8 (69.6, 96.4) | 82.6 (57.7, 96.3) | 41.3 (13.2, 73.7) | 88.8 (70.9, 97.9) |
| BORNO | BAYO | 61.7 (29.6, 87.7) | 82.5 (59.9, 94.6) | 69.1 (31.7, 93.3) | 58.2 (27.3, 83.3) | 86.0 (62.2, 97.6) |
| BORNO | BIU | 66.0 (35.5, 89.1) | 87.7 (74.2, 95.9) | 81.9 (53.0, 96.5) | 61.4 (33.6, 82.8) | 92.3 (78.5, 98.7) |
| BORNO | CHIBOK | 35.0 (16.6, 57.8) | 77.2 (60.6, 90.2) | 73.2 (50.4, 89.9) | 31.7 (14.2, 51.5) | 80.5 (63.1, 92.8) |
| BORNO | DAMBOA | 45.5 (22.7, 71.0) | 83.1 (68.0, 93.0) | 77.8 (55.1, 91.5) | 42.6 (21.2, 65.6) | 86.0 (70.1, 95.3) |
| BORNO | DIKWA | 41.3 (10.3, 79.3) | 87.3 (67.5, 97.2) | 83.5 (58.1, 97.6) | 39.5 (10.2, 75.7) | 89.1 (68.2, 98.7) |
| BORNO | GUBIO | 43.2 (21.4, 68.5) | 88.0 (73.3, 96.2) | 83.9 (62.2, 95.9) | 41.8 (20.8, 66.5) | 89.4 (74.3, 97.2) |
| BORNO | GUZAMALA | 35.6 (12.2, 65.4) | 81.9 (62.4, 93.8) | 76.6 (50.8, 93.1) | 34.1 (11.8, 61.4) | 83.3 (63.2, 95.6) |
| BORNO | GWOZA | 49.4 (14.8, 83.8) | 88.4 (69.2, 97.5) | 84.7 (55.5, 98.0) | 46.7 (14.1, 79.7) | 91.0 (72.1, 98.9) |
| BORNO | HAWUL | 69.5 (43.5, 88.7) | 89.6 (77.8, 96.8) | 83.9 (58.9, 97.7) | 65.4 (41.4, 84.2) | 93.8 (81.9, 99.2) |
| BORNO | JERE | 52.3 (33.1, 70.8) | 80.9 (67.4, 91.2) | 75.2 (53.7, 91.3) | 47.0 (28.3, 64.7) | 86.3 (72.5, 95.6) |
| BORNO | KAGA | 40.9 (15.6, 68.8) | 83.5 (65.9, 95.6) | 78.3 (52.7, 95.1) | 39.2 (14.9, 66.2) | 85.1 (66.8, 96.5) |
| BORNO | KALA/BALGE | 40.3 (08.3, 80.4) | 88.6 (67.0, 98.1) | 85.3 (54.7, 98.5) | 38.8 (08.1, 77.5) | 90.1 (67.6, 99.1) |
| BORNO | KONDUGA | 51.0 (35.0, 66.8) | 78.0 (68.3, 85.5) | 69.5 (54.7, 81.5) | 46.1 (30.9, 60.8) | 82.9 (72.5, 90.2) |
| BORNO | KUKAWA | 41.2 (11.9, 74.2) | 85.3 (65.6, 96.0) | 80.7 (51.5, 96.4) | 39.3 (11.6, 70.0) | 87.2 (67.8, 97.8) |
| BORNO | KWAYA | 61.0 (23.9, 89.3) | 83.2 (58.2, 95.8) | 71.8 (33.2, 96.0) | 57.6 (23.1, 85.2) | 86.6 (60.3, 98.3) |
| BORNO | MAFA | 41.1 (12.3, 75.7) | 85.5 (66.2, 96.3) | 81.6 (54.6, 96.5) | 39.0 (12.0, 71.7) | 87.6 (67.4, 97.7) |
| BORNO | MAGUMERI | 24.1 (11.9, 40.2) | 75.1 (61.6, 85.7) | 71.1 (54.6, 83.5) | 23.3 (11.5, 38.7) | 75.9 (62.1, 86.7) |
| BORNO | MAIDUGURI | 63.3 (46.0, 77.4) | 78.5 (66.3, 88.4) | 71.5 (49.6, 87.9) | 54.6 (38.8, 68.5) | 87.2 (75.2, 95.2) |
| BORNO | MARTE | 39.9 (09.9, 77.8) | 86.0 (62.3, 97.1) | 82.0 (50.2, 97.5) | 38.3 (09.6, 74.0) | 87.7 (62.7, 98.3) |
| BORNO | MOBBAR | 44.2 (13.6, 81.3) | 89.0 (73.1, 97.5) | 85.7 (62.2, 97.9) | 42.2 (13.0, 77.6) | 91.0 (74.5, 98.8) |
| BORNO | MONGUNO | 32.1 (09.0, 62.3) | 80.2 (56.6, 94.2) | 75.1 (44.4, 93.6) | 30.9 (08.9, 59.9) | 81.5 (57.1, 95.7) |
| BORNO | NGALA | 42.3 (10.0, 77.7) | 88.0 (67.9, 97.7) | 84.5 (52.5, 98.1) | 40.4 (09.9, 75.2) | 89.9 (70.3, 98.8) |
| BORNO | NGANZAI | 24.2 (08.4, 44.7) | 78.2 (60.3, 90.9) | 74.5 (52.4, 90.1) | 23.3 (08.2, 43.3) | 79.1 (60.8, 92.3) |
| BORNO | SHANI | 67.0 (40.3, 87.0) | 89.5 (75.0, 97.1) | 79.8 (48.9, 95.9) | 64.8 (38.7, 84.5) | 91.7 (77.3, 98.7) |
| CROSS RIVER | ABI | 72.9 (54.1, 87.0) | 93.2 (83.3, 98.2) | 87.2 (58.0, 98.7) | 70.7 (52.6, 84.5) | 95.5 (85.3, 99.6) |
| CROSS RIVER | AKAMKPA | 57.4 (34.9, 76.8) | 90.1 (81.2, 96.0) | 90.0 (74.7, 97.9) | 52.7 (32.9, 69.9) | 94.9 (86.3, 99.0) |
| CROSS RIVER | AKPABUYO | 68.3 (47.3, 85.7) | 86.1 (72.0, 94.5) | 88.9 (67.8, 98.4) | 58.8 (40.4, 76.3) | 95.5 (86.2, 99.5) |
| CROSS RIVER | BAKASSI | 71.1 (36.1, 93.6) | 83.6 (61.3, 96.1) | 86.4 (53.6, 98.0) | 59.4 (26.9, 84.9) | 95.3 (81.7, 99.8) |
| CROSS RIVER | BEKWARA | 66.5 (45.7, 84.2) | 91.7 (79.3, 97.9) | 87.1 (59.9, 98.4) | 63.9 (43.6, 80.4) | 94.4 (82.6, 99.3) |
| CROSS RIVER | BIASE | 65.7 (44.8, 83.9) | 92.4 (82.7, 97.8) | 89.3 (68.8, 98.5) | 62.5 (42.2, 80.6) | 95.6 (86.2, 99.4) |
| CROSS RIVER | BOKI | 64.0 (35.9, 88.2) | 91.0 (79.9, 97.2) | 88.2 (68.6, 98.1) | 60.3 (33.2, 83.2) | 94.7 (84.6, 99.2) |
| CROSS RIVER | CALABAR MUNICIPAL | 66.5 (42.5, 85.4) | 87.5 (71.9, 96.3) | 87.1 (64.4, 98.4) | 59.1 (37.0, 77.7) | 94.9 (83.6, 99.5) |
| CROSS RIVER | CALABAR SOUTH | 69.1 (47.1, 87.6) | 86.7 (68.6, 96.8) | 75.4 (55.6, 82.9) | 62.1 (40.2, 81.3) | 93.7 (79.4, 99.6) |
| CROSS RIVER | ETUNG | 66.0 (33.0, 91.9) | 90.6 (74.0, 98.1) | 84.7 (50.3, 98.7) | 63.0 (31.5, 87.6) | 93.6 (76.0, 99.5) |
| CROSS RIVER | IKOM | 65.6 (31.2, 91.1) | 90.0 (73.8, 97.4) | 84.4 (57.5, 97.9) | 62.3 (29.3, 85.7) | 93.3 (78.1, 99.4) |
| CROSS RIVER | OBANLIKU | 76.0 (57.7, 89.4) | 85.6 (71.4, 94.4) | 88.6 (61.6, 98.7) | 65.1 (47.5, 80.5) | 96.4 (88.3, 99.6) |
| CROSS RIVER | OBUBRA | 68.5 (43.6, 88.9) | 90.9 (78.7, 97.3) | 83.2 (58.1, 97.0) | 66.1 (42.1, 85.6) | 93.3 (80.9, 98.9) |
| CROSS RIVER | OBUDU | 78.8 (59.2, 92.3) | 89.5 (78.4, 96.2) | 88.5 (60.3, 98.7) | 71.5 (52.6, 86.0) | 96.7 (88.2, 99.7) |
| CROSS RIVER | ODUKPANI | 61.8 (43.3, 78.7) | 89.9 (80.6, 96.0) | 91.6 (77.1, 98.3) | 55.8 (39.0, 71.0) | 95.9 (88.5, 99.2) |
| CROSS RIVER | OGOJA | 64.0 (38.0, 86.4) | 90.7 (77.1, 97.4) | 86.2 (62.8, 97.7) | 60.8 (36.3, 82.5) | 93.8 (80.9, 99.0) |
| CROSS RIVER | YAKURR | 73.4 (53.3, 88.6) | 92.1 (80.6, 98.0) | 84.6 (50.7, 98.2) | 70.8 (50.8, 85.3) | 94.7 (83.0, 99.3) |
| CROSS RIVER | YALA | 62.0 (36.3, 83.2) | 87.9 (74.6, 95.9) | 79.6 (51.8, 95.0) | 59.0 (35.2, 78.9) | 90.9 (76.6, 98.0) |
| DELTA | ANIOCHA NORTH | 73.0 (51.8, 89.9) | 93.8 (84.6, 98.3) | 90.5 (68.0, 99.3) | 70.2 (49.7, 86.1) | 96.6 (87.9, 99.8) |
| DELTA | ANIOCHA SOUTH | 73.6 (55.7, 87.7) | 94.2 (85.8, 98.4) | 89.7 (65.3, 99.0) | 71.3 (54.1, 85.8) | 96.5 (88.4, 99.7) |
| DELTA | BOMADI | 71.7 (44.5, 91.5) | 91.3 (77.2, 98.3) | 83.9 (49.6, 98.5) | 68.8 (43.1, 88.4) | 94.3 (80.2, 99.6) |
| DELTA | BURUTU | 68.1 (46.2, 85.8) | 91.4 (81.8, 96.9) | 86.1 (65.2, 96.2) | 64.9 (44.1, 81.9) | 94.6 (85.4, 98.9) |
| DELTA | ETHIOPE EAST | 59.1 (38.5, 78.3) | 90.0 (78.2, 96.7) | 89.6 (70.2, 98.2) | 54.5 (35.8, 72.9) | 94.6 (83.8, 99.1) |
| DELTA | ETHIOPE WEST | 57.9 (31.9, 81.9) | 94.0 (83.8, 98.6) | 92.5 (74.3, 99.2) | 55.9 (31.5, 79.1) | 96.1 (86.3, 99.6) |
| DELTA | IKA NORTH EAST | 60.4 (38.2, 78.5) | 92.9 (83.5, 98.0) | 90.6 (73.7, 98.5) | 58.0 (36.8, 76.0) | 95.3 (86.2, 99.3) |
| DELTA | IKA SOUTH | 71.7 (48.3, 87.9) | 92.1 (80.3, 97.8) | 89.4 (63.1, 98.6) | 67.6 (44.4, 83.8) | 96.2 (87.7, 99.5) |
| DELTA | ISOKO NORTH | 53.1 (34.7, 70.0) | 92.0 (83.5, 97.2) | 89.4 (75.0, 97.5) | 51.1 (33.1, 67.2) | 94.0 (85.9, 98.6) |
| DELTA | ISOKO SOUTH | 62.9 (41.5, 80.7) | 92.8 (82.5, 97.8) | 89.8 (70.4, 98.4) | 60.5 (39.9, 77.5) | 95.1 (85.3, 99.3) |
| DELTA | NDOKWA EAST | 56.8 (36.5, 76.1) | 92.0 (81.4, 97.4) | 89.6 (72.6, 97.9) | 54.1 (34.5, 73.1) | 94.7 (85.3, 98.9) |
| DELTA | NDOKWA WEST | 42.3 (24.0, 61.2) | 94.1 (85.8, 98.3) | 93.4 (81.7, 98.8) | 40.9 (23.5, 59.6) | 95.5 (87.3, 99.2) |
| DELTA | OKPE | 70.4 (47.4, 89.0) | 93.0 (82.2, 98.2) | 90.8 (67.3, 99.2) | 66.9 (45.3, 85.2) | 96.5 (87.6, 99.7) |
| DELTA | OSHIMILI NORTH | 85.1 (73.3, 93.3) | 93.9 (86.8, 97.9) | 89.6 (63.4, 98.9) | 81.1 (69.3, 90.0) | 97.9 (92.9, 99.8) |
| DELTA | OSHIMILI | 91.8 (82.9, 97.3) | 95.7 (89.8, 98.8) | 87.3 (55.0, 98.9) | 89.0 (80.0, 95.2) | 98.5 (94.3, 99.9) |
| DELTA | PATANI | 72.3 (49.9, 89.3) | 92.8 (80.7, 98.2) | 86.2 (55.3, 98.4) | 70.2 (48.5, 86.4) | 95.0 (82.7, 99.5) |
| DELTA | SEPELE | 60.8 (38.6, 80.9) | 91.0 (78.3, 97.2) | 90.2 (68.4, 98.7) | 56.6 (37.0, 75.5) | 95.2 (83.8, 99.4) |
| DELTA | UDU | 84.3 (64.5, 95.9) | 93.4 (83.7, 98.7) | 88.6 (54.3, 99.4) | 80.2 (61.2, 92.9) | 97.5 (90.6, 99.9) |
| DELTA | UGHELLI NORTH | 66.5 (44.5, 83.4) | 93.4 (84.8, 97.8) | 90.2 (73.0, 98.3) | 63.9 (43.2, 80.2) | 96.0 (88.9, 99.3) |
| DELTA | UGHELLI SOUTH | 75.9 (51.5, 91.3) | 93.3 (83.8, 98.2) | 88.2 (63.7, 98.8) | 73.0 (49.9, 88.9) | 96.2 (87.7, 99.7) |
| DELTA | UKWUANI | 44.6 (19.5, 72.1) | 94.2 (80.6, 98.9) | 93.1 (73.2, 98.8) | 43.2 (19.0, 69.4) | 95.6 (82.2, 99.5) |
| DELTA | UVWIE | 86.2 (68.8, 96.5) | 92.1 (81.9, 98.1) | 83.3 (44.7, 98.7) | 81.3 (63.7, 92.9) | 97.1 (89.4, 99.8) |
| DELTA | WARRI NORTH | 62.6 (33.7, 85.5) | 89.5 (77.0, 96.7) | 88.9 (68.3, 98.0) | 57.2 (32.0, 79.9) | 94.9 (84.9, 99.2) |
| DELTA | WARRI SOUTH | 80.3 (62.0, 93.0) | 92.5 (83.3, 98.0) | 81.1 (52.0, 97.9) | 76.2 (58.9, 89.8) | 96.6 (87.9, 99.7) |
| DELTA | WARRI SOUTH WEST | 69.4 (45.4, 89.0) | 91.7 (81.3, 97.4) | 89.1 (70.1, 97.8) | 65.1 (43.7, 83.9) | 96.0 (86.9, 99.5) |
| EBONYI | ABAKALIKI | 78.7 (63.5, 90.2) | 90.1 (79.9, 96.2) | 78.5 (53.4, 94.2) | 75.0 (60.2, 87.0) | 93.8 (84.5, 98.5) |
| EBONYI | AFIKPO NORTH | 77.0 (57.6, 90.3) | 93.7 (84.2, 98.4) | 88.7 (63.3, 99.0) | 74.2 (55.4, 87.6) | 96.5 (88.2, 99.7) |
| EBONYI | AFIKPO SOUTH | 81.2 (62.8, 92.9) | 93.8 (85.0, 98.4) | 86.6 (56.0, 98.9) | 78.4 (60.7, 90.1) | 96.6 (88.4, 99.8) |
| EBONYI | EBONYI | 75.0 (61.9, 85.0) | 85.0 (75.4, 91.7) | 72.0 (48.1, 87.9) | 69.8 (56.4, 80.3) | 90.2 (82.7, 95.6) |
| EBONYI | EZZA NORTH | 68.3 (51.4, 82.7) | 91.7 (82.1, 97.2) | 86.7 (66.0, 97.5) | 65.4 (49.6, 78.9) | 94.6 (85.9, 99.0) |
| EBONYI | EZZA SOUTH | 70.7 (53.8, 84.4) | 93.3 (85.1, 97.9) | 87.7 (68.4, 97.5) | 68.7 (52.8, 81.9) | 95.3 (86.9, 99.1) |
| EBONYI | IKWO | 70.2 (49.2, 87.0) | 90.3 (79.7, 96.9) | 80.6 (56.2, 95.9) | 67.9 (47.8, 84.2) | 92.6 (81.6, 98.5) |
| EBONYI | ISHIELU | 70.6 (53.3, 84.9) | 91.2 (82.1, 96.4) | 87.1 (66.5, 96.9) | 66.9 (50.7, 81.4) | 94.9 (87.4, 98.9) |
| EBONYI | IVO | 71.3 (50.7, 86.8) | 92.5 (81.5, 98.2) | 87.0 (59.4, 98.4) | 68.6 (48.7, 84.3) | 95.2 (84.6, 99.5) |
| EBONYI | IZZI | 66.9 (43.6, 84.4) | 81.9 (66.5, 91.7) | 63.9 (34.9, 86.3) | 63.4 (40.8, 80.4) | 85.3 (69.3, 94.9) |
| EBONYI | OHAOZARA | 71.3 (53.7, 85.8) | 95.3 (87.3, 98.7) | 92.8 (73.3, 99.2) | 69.5 (52.5, 83.4) | 97.2 (90.3, 99.7) |
| EBONYI | OHAUKWU | 64.6 (48.9, 78.1) | 82.7 (71.9, 90.5) | 70.3 (49.1, 86.6) | 60.5 (46.1, 74.0) | 86.7 (76.1, 94.1) |
| EBONYI | ONITCHA | 69.7 (52.4, 83.3) | 95.1 (88.6, 98.4) | 93.4 (79.8, 99.0) | 67.5 (51.2, 81.4) | 97.2 (91.4, 99.6) |
| EDO | AKOKO EDO | 65.5 (45.2, 81.2) | 94.9 (87.9, 98.5) | 93.5 (78.9, 99.0) | 63.4 (43.8, 78.7) | 97.1 (90.8, 99.5) |
| EDO | EGOR | 90.7 (75.9, 97.9) | 77.8 (55.5, 90.9) | 57.1 (25.6, 87.3) | 73.0 (49.9, 88.1) | 95.6 (86.7, 99.4) |
| EDO | ESAN CENTRAL | 62.2 (40.9, 81.2) | 93.1 (83.9, 98.1) | 93.5 (77.8, 99.4) | 58.5 (39.1, 76.3) | 96.8 (89.0, 99.7) |
| EDO | ESAN NORTH EAST | 60.0 (41.3, 76.5) | 93.7 (85.6, 98.1) | 94.1 (80.9, 99.3) | 56.7 (38.9, 72.2) | 97.0 (89.8, 99.7) |
| EDO | ESAN SOUTH EAST | 64.7 (46.2, 81.4) | 93.4 (84.7, 97.7) | 92.3 (76.9, 98.9) | 61.6 (43.8, 77.5) | 96.6 (89.4, 99.5) |
| EDO | ESAN WEST | 71.0 (45.6, 90.2) | 90.5 (77.8, 97.2) | 90.8 (67.7, 99.2) | 65.0 (41.3, 83.5) | 96.5 (87.6, 99.7) |
| EDO | ETSAKO CENTRAL | 68.1 (47.0, 85.6) | 93.4 (84.1, 98.2) | 89.8 (67.3, 98.7) | 65.7 (45.8, 82.6) | 95.8 (86.7, 99.5) |
| EDO | ETSAKO EAST | 59.9 (44.0, 75.8) | 94.7 (87.9, 98.3) | 92.4 (78.6, 98.6) | 58.2 (42.6, 73.4) | 96.4 (89.5, 99.3) |
| EDO | ETSAKO WEST | 56.1 (33.3, 76.6) | 90.2 (79.6, 96.6) | 87.4 (68.9, 97.2) | 53.1 (31.6, 72.6) | 93.1 (83.2, 98.7) |
| EDO | IGUEBEN | 67.4 (46.8, 84.5) | 93.5 (84.3, 98.2) | 92.5 (75.0, 99.3) | 64.2 (44.9, 81.8) | 96.8 (89.0, 99.7) |
| EDO | IKPOBA OKHA | 86.9 (72.8, 95.8) | 75.9 (58.7, 88.5) | 78.5 (48.7, 95.2) | 66.3 (48.4, 80.6) | 96.5 (89.8, 99.4) |
| EDO | OREDO | 88.2 (70.3, 97.3) | 75.0 (54.6, 88.9) | 77.2 (42.1, 96.3) | 66.8 (45.6, 83.8) | 96.4 (89.0, 99.6) |
| EDO | ORHIONMWON | 68.6 (49.6, 84.8) | 91.1 (82.5, 96.7) | 87.7 (68.4, 97.7) | 64.4 (46.8, 80.4) | 95.4 (87.1, 99.3) |
| EDO | OVIA NORTH EAST | 85.5 (68.8, 95.0) | 85.1 (72.7, 93.1) | 79.7 (54.7, 94.8) | 74.4 (59.3, 86.6) | 96.1 (89.2, 99.2) |
| EDO | OVIA SOUTH WEST | 80.1 (62.6, 91.9) | 88.4 (78.4, 95.0) | 80.0 (54.6, 95.3) | 73.5 (56.8, 86.1) | 95.0 (87.6, 99.0) |
| EDO | OWAN EAST | 62.2 (40.2, 81.8) | 93.0 (83.3, 97.7) | 91.0 (72.0, 98.6) | 59.6 (38.7, 78.0) | 95.6 (86.8, 99.4) |
| EDO | OWAN WEST | 71.4 (45.4, 90.1) | 92.7 (82.2, 98.0) | 90.1 (65.8, 99.1) | 67.7 (43.0, 85.7) | 96.4 (86.8, 99.7) |
| EDO | UHUNMWONDE | 86.1 (70.0, 95.7) | 84.6 (71.5, 94.0) | 82.6 (56.4, 96.5) | 73.8 (57.1, 86.9) | 96.9 (90.3, 99.5) |
| EKITI | ADO-EKITI | 80.2 (64.0, 92.3) | 94.1 (85.5, 98.5) | 89.1 (64.3, 99.0) | 77.3 (61.4, 89.3) | 97.0 (90.0, 99.7) |
| EKITI | EFON | 87.7 (71.0, 96.9) | 94.5 (86.4, 98.7) | 87.3 (55.6, 98.9) | 84.4 (68.7, 94.6) | 97.8 (91.1, 99.9) |
| EKITI | EKITI EAST | 75.1 (55.4, 90.1) | 92.5 (82.8, 98.1) | 90.7 (69.7, 98.9) | 70.7 (50.9, 85.9) | 96.9 (89.8, 99.7) |
| EKITI | EKITI SOUTH WEST | 82.9 (66.7, 93.8) | 95.0 (88.3, 98.6) | 89.6 (63.7, 99.0) | 80.4 (64.5, 91.4) | 97.5 (91.9, 99.8) |
| EKITI | EKITI WEST | 85.6 (69.2, 95.1) | 94.6 (87.1, 98.5) | 88.1 (56.7, 99.0) | 82.6 (66.3, 92.7) | 97.6 (91.4, 99.8) |
| EKITI | EMURE | 83.8 (67.2, 94.1) | 94.6 (85.1, 98.8) | 90.4 (65.7, 99.2) | 80.6 (63.5, 91.8) | 97.8 (91.5, 99.8) |
| EKITI | GBONYIN | 82.3 (66.3, 93.7) | 93.4 (85.3, 98.1) | 88.8 (62.9, 98.7) | 78.5 (62.7, 89.9) | 97.2 (91.0, 99.7) |
| EKITI | IDO-OSI | 86.1 (70.9, 95.2) | 93.9 (85.7, 98.4) | 84.8 (49.9, 98.7) | 82.9 (68.3, 92.5) | 97.0 (88.7, 99.8) |
| EKITI | IJERO | 88.6 (73.2, 96.6) | 94.0 (85.9, 98.3) | 86.9 (53.1, 99.3) | 84.7 (70.0, 93.8) | 97.9 (91.8, 99.9) |
| EKITI | IKERE | 85.3 (69.4, 95.1) | 95.2 (87.2, 98.9) | 89.9 (61.2, 99.3) | 82.6 (66.9, 93.0) | 97.9 (91.9, 99.9) |
| EKITI | IKOLE | 81.5 (66.4, 92.8) | 88.1 (77.2, 95.1) | 77.0 (49.0, 94.1) | 75.0 (60.6, 86.9) | 94.6 (87.2, 98.8) |
| EKITI | ILEJEMEJE | 87.0 (73.0, 95.7) | 94.0 (84.8, 98.7) | 82.9 (39.2, 99.0) | 84.1 (70.0, 93.5) | 96.9 (88.9, 99.8) |
| EKITI | IREPODUN/IFELODUN | 80.1 (64.2, 92.0) | 93.6 (85.4, 98.2) | 87.0 (62.0, 98.4) | 77.2 (61.7, 89.3) | 96.5 (88.9, 99.6) |
| EKITI | ISE ORUN | 86.9 (72.1, 95.3) | 94.5 (85.7, 98.6) | 89.4 (61.6, 99.1) | 83.4 (68.6, 92.6) | 98.0 (92.9, 99.8) |
| EKITI | MOBA | 89.0 (76.1, 96.6) | 94.5 (85.9, 98.8) | 86.2 (48.1, 99.3) | 85.6 (72.7, 94.3) | 97.9 (91.5, 99.9) |
| EKITI | OYE | 82.2 (62.9, 93.9) | 88.7 (76.4, 96.1) | 75.8 (40.1, 96.1) | 76.5 (57.8, 89.1) | 94.4 (84.1, 99.2) |
| ENUGU | ANINRI | 74.0 (56.1, 88.6) | 93.9 (85.0, 98.3) | 90.4 (68.6, 98.9) | 71.4 (54.0, 86.2) | 96.6 (88.3, 99.7) |
| ENUGU | AWGU | 81.8 (64.8, 92.6) | 94.6 (87.0, 98.6) | 89.1 (64.8, 98.9) | 79.2 (62.8, 90.1) | 97.2 (90.2, 99.7) |
| ENUGU | ENUGU EAST | 86.5 (72.0, 95.8) | 90.4 (79.2, 96.7) | 90.7 (67.3, 98.9) | 78.8 (63.4, 90.2) | 98.2 (93.6, 99.8) |
| ENUGU | ENUGU NORTH | 89.3 (77.5, 96.3) | 90.4 (80.1, 96.6) | 91.2 (67.5, 99.2) | 81.1 (68.4, 90.4) | 98.6 (94.5, 99.9) |
| ENUGU | ENUGU SOUTH | 90.0 (78.9, 96.5) | 93.5 (86.0, 98.1) | 92.3 (70.7, 99.3) | 84.7 (73.2, 93.2) | 98.8 (95.2, 99.9) |
| ENUGU | EZEAGU | 82.7 (65.4, 94.7) | 94.6 (86.6, 98.6) | 87.3 (60.3, 98.4) | 80.4 (63.4, 92.0) | 96.9 (89.1, 99.7) |
| ENUGU | IGBOETITI | 81.9 (64.0, 93.2) | 88.9 (76.8, 96.4) | 83.2 (53.8, 97.6) | 75.0 (57.5, 87.4) | 95.8 (87.4, 99.4) |
| ENUGU | IGBOEZE NORTH | 86.5 (70.8, 96.0) | 90.3 (80.0, 96.6) | 84.5 (51.8, 98.2) | 79.7 (64.2, 90.6) | 97.1 (89.4, 99.7) |
| ENUGU | IGBOEZE SOUTH | 88.5 (75.2, 96.4) | 89.5 (78.2, 96.0) | 84.7 (55.7, 98.1) | 80.5 (66.5, 90.9) | 97.5 (91.9, 99.7) |
| ENUGU | ISIUZO | 58.3 (39.3, 76.2) | 88.5 (77.3, 95.6) | 85.3 (66.3, 96.1) | 54.7 (36.6, 71.0) | 92.1 (81.5, 97.8) |
| ENUGU | NKANU EAST | 81.6 (68.3, 90.5) | 94.1 (88.0, 97.7) | 92.7 (77.8, 98.9) | 77.7 (64.5, 86.7) | 98.0 (93.9, 99.7) |
| ENUGU | NKANU WEST | 87.9 (75.5, 95.4) | 95.6 (89.9, 98.8) | 92.8 (72.2, 99.4) | 84.9 (72.9, 93.4) | 98.7 (94.9, 99.9) |
| ENUGU | NSUKKA | 84.8 (73.0, 93.8) | 87.5 (77.0, 94.8) | 82.4 (55.4, 97.2) | 76.0 (62.5, 86.5) | 96.3 (90.0, 99.4) |
| ENUGU | OJI-RIVER | 82.1 (66.0, 92.6) | 95.6 (89.7, 98.9) | 88.8 (67.9, 98.7) | 80.5 (64.8, 91.0) | 97.2 (91.2, 99.7) |
| ENUGU | UDENU | 77.5 (60.3, 90.2) | 87.8 (76.8, 94.9) | 81.0 (55.3, 95.5) | 71.0 (53.5, 84.4) | 94.3 (86.4, 98.7) |
| ENUGU | UDI | 85.3 (71.7, 94.5) | 93.8 (87.1, 97.7) | 87.6 (65.2, 97.8) | 81.8 (69.3, 91.3) | 97.3 (91.7, 99.5) |
| ENUGU | UZO-UWANI | 74.6 (50.9, 89.8) | 88.9 (77.1, 95.6) | 80.9 (54.7, 95.0) | 69.8 (47.9, 84.7) | 93.6 (81.6, 98.7) |
| FCT | ABAJI | 37.7 (21.1, 56.4) | 94.0 (86.6, 98.3) | 92.8 (81.2, 98.4) | 36.9 (20.8, 55.0) | 94.8 (87.3, 98.9) |
| FCT | AMAC | 65.6 (51.1, 77.2) | 94.1 (87.0, 97.9) | 93.1 (80.0, 98.7) | 62.4 (48.3, 74.3) | 97.2 (91.8, 99.4) |
| FCT | BWARI | 75.9 (59.7, 88.2) | 93.6 (85.5, 98.0) | 90.3 (71.4, 98.6) | 72.6 (56.2, 84.3) | 96.9 (90.3, 99.6) |
| FCT | GWAGWALADA | 36.2 (21.7, 52.9) | 95.8 (89.5, 98.8) | 95.1 (86.5, 99.0) | 35.6 (21.3, 51.8) | 96.4 (89.8, 99.3) |
| FCT | KUJE | 43.8 (23.4, 65.1) | 94.9 (87.5, 98.6) | 93.7 (82.2, 98.8) | 42.9 (23.2, 64.0) | 95.8 (88.4, 99.2) |
| FCT | KWALI | 30.6 (17.7, 47.4) | 94.2 (87.9, 98.0) | 93.3 (84.9, 98.0) | 30.1 (17.6, 46.5) | 94.6 (88.3, 98.4) |
| GOMBE | AKKO | 67.8 (53.0, 80.6) | 87.6 (77.9, 94.2) | 81.6 (61.3, 93.8) | 63.3 (49.9, 75.6) | 92.1 (84.3, 97.4) |
| GOMBE | BALANGA | 70.6 (51.4, 86.3) | 89.8 (79.8, 96.2) | 76.1 (52.3, 91.8) | 68.8 (49.9, 83.8) | 91.6 (81.9, 97.4) |
| GOMBE | BILLIRI | 60.0 (41.2, 75.7) | 92.7 (84.2, 97.5) | 90.0 (74.2, 97.8) | 57.9 (40.2, 73.5) | 94.8 (86.7, 98.8) |
| GOMBE | DUKKU | 43.6 (24.0, 65.4) | 85.1 (71.2, 94.6) | 80.5 (59.8, 93.7) | 42.0 (23.6, 62.1) | 86.7 (71.9, 96.1) |
| GOMBE | FUNAKAYE | 29.9 (15.4, 49.3) | 88.5 (78.7, 95.1) | 87.9 (75.7, 95.3) | 28.0 (14.4, 45.4) | 90.5 (80.9, 96.4) |
| GOMBE | GOMBE | 78.6 (56.5, 93.5) | 90.8 (76.8, 98.0) | 81.7 (49.6, 97.4) | 74.7 (52.7, 90.5) | 94.7 (82.5, 99.5) |
| GOMBE | KALTUNGO | 67.9 (46.4, 85.4) | 90.8 (80.9, 96.9) | 81.6 (59.8, 95.0) | 66.0 (45.0, 83.1) | 92.6 (82.2, 98.2) |
| GOMBE | KWANI | 51.3 (32.1, 69.3) | 89.8 (80.0, 96.2) | 85.8 (68.7, 95.9) | 49.3 (31.3, 66.7) | 91.8 (81.6, 97.6) |
| GOMBE | NAFADA | 40.7 (22.0, 61.3) | 90.0 (78.0, 96.9) | 87.1 (69.3, 96.7) | 39.4 (21.3, 58.8) | 91.3 (79.2, 97.9) |
| GOMBE | SHOMGOM | 65.9 (45.1, 83.4) | 93.5 (84.8, 98.1) | 91.0 (73.3, 98.4) | 63.5 (43.2, 80.4) | 95.9 (87.8, 99.4) |
| GOMBE | YAMALTU/DEBA | 69.8 (50.6, 84.6) | 87.7 (76.8, 94.7) | 75.3 (50.5, 91.5) | 66.8 (48.4, 81.4) | 90.7 (80.0, 97.0) |
| IMO | ABOH MBAISE | 89.4 (75.6, 96.7) | 89.8 (79.3, 96.5) | 75.8 (37.2, 96.6) | 82.6 (69.6, 92.0) | 96.6 (89.7, 99.6) |
| IMO | AHIAZU MBAISE | 91.4 (80.0, 97.3) | 89.0 (75.7, 96.2) | 74.2 (27.4, 97.2) | 83.3 (68.7, 92.8) | 97.0 (90.7, 99.7) |
| IMO | EHIME MBANO | 89.7 (75.8, 96.9) | 88.7 (76.9, 95.8) | 73.7 (29.5, 96.4) | 82.1 (66.6, 92.0) | 96.3 (88.1, 99.6) |
| IMO | EZINIHITTE MBAISE | 88.2 (73.0, 96.4) | 89.1 (77.0, 96.3) | 70.8 (27.1, 96.7) | 81.8 (66.0, 92.1) | 95.6 (86.6, 99.6) |
| IMO | IDEATO SOUTH | 90.1 (78.8, 96.4) | 91.5 (83.1, 96.9) | 68.9 (28.2, 94.3) | 85.7 (73.7, 93.5) | 95.9 (89.2, 99.3) |
| IMO | IDEATO NORTH | 86.3 (72.2, 94.9) | 90.2 (80.8, 96.2) | 65.3 (31.1, 90.6) | 82.7 (69.1, 91.6) | 93.9 (85.6, 98.8) |
| IMO | IHITTE UBOMA | 85.4 (67.7, 95.8) | 86.6 (72.2, 95.4) | 72.3 (30.1, 95.9) | 77.4 (58.9, 89.6) | 94.7 (83.4, 99.4) |
| IMO | IKEDURU | 92.1 (83.2, 97.2) | 91.3 (83.4, 96.6) | 80.4 (42.8, 97.8) | 85.6 (75.6, 92.8) | 97.8 (93.3, 99.8) |
| IMO | ISIALA MBANO | 92.4 (83.0, 97.4) | 91.5 (83.2, 96.6) | 75.9 (37.0, 96.5) | 86.4 (76.5, 93.3) | 97.4 (92.2, 99.7) |
| IMO | ISU | 91.3 (80.7, 97.2) | 91.7 (83.0, 97.3) | 78.8 (45.2, 96.3) | 85.6 (73.9, 94.0) | 97.4 (91.8, 99.7) |
| IMO | MBAITOLI | 89.9 (78.0, 96.4) | 90.9 (80.9, 96.7) | 83.5 (56.2, 97.5) | 83.2 (69.6, 91.8) | 97.6 (93.1, 99.7) |
| IMO | NGOR OKPALA | 77.5 (56.8, 92.3) | 87.5 (75.5, 95.5) | 75.6 (41.5, 95.4) | 72.1 (52.7, 86.6) | 92.9 (81.0, 98.8) |
| IMO | NJABA | 87.5 (75.9, 95.3) | 91.7 (83.2, 96.8) | 80.2 (51.4, 95.7) | 82.7 (70.9, 91.6) | 96.5 (90.5, 99.4) |
| IMO | NKWERRE | 92.0 (81.4, 97.8) | 91.7 (81.7, 97.5) | 70.9 (25.4, 96.4) | 86.8 (73.9, 95.1) | 96.9 (90.5, 99.8) |
| IMO | NWANGELE | 92.7 (83.5, 98.1) | 91.4 (81.0, 97.4) | 73.6 (30.1, 96.8) | 86.8 (74.2, 94.7) | 97.3 (91.2, 99.8) |
| IMO | OBOWO | 86.0 (69.3, 95.6) | 87.8 (73.4, 95.7) | 70.2 (25.5, 96.6) | 79.1 (62.3, 90.6) | 94.7 (83.8, 99.4) |
| IMO | OGUTA | 74.1 (54.7, 88.3) | 87.8 (76.8, 94.8) | 85.2 (63.3, 96.4) | 67.0 (49.9, 81.4) | 94.9 (86.2, 99.0) |
| IMO | OHAJI EGBEMA | 70.2 (50.8, 85.7) | 82.6 (69.7, 91.5) | 78.3 (54.9, 93.3) | 61.0 (42.1, 76.9) | 91.9 (81.4, 97.7) |
| IMO | OKIGWE | 82.1 (62.2, 94.4) | 89.9 (78.1, 97.1) | 74.7 (35.9, 95.8) | 77.8 (58.4, 90.9) | 94.1 (83.5, 99.3) |
| IMO | ORLU | 87.4 (75.7, 94.1) | 90.8 (83.0, 96.2) | 70.3 (33.0, 93.9) | 83.2 (71.5, 90.9) | 95.0 (87.5, 99.0) |
| IMO | ORSU | 85.7 (71.5, 95.1) | 90.7 (80.5, 96.6) | 71.1 (35.9, 93.2) | 81.8 (67.4, 91.8) | 94.6 (86.0, 98.9) |
| IMO | ORU EAST | 82.6 (70.4, 91.9) | 90.6 (82.6, 95.8) | 80.1 (55.2, 94.6) | 77.8 (65.5, 87.4) | 95.3 (88.5, 99.0) |
| IMO | ORU WEST | 79.7 (63.1, 91.6) | 89.1 (77.6, 95.9) | 79.7 (45.6, 96.0) | 74.2 (57.9, 87.1) | 94.5 (84.6, 99.1) |
| IMO | OWERRI MUNICIPAL | 88.5 (72.1, 97.5) | 87.5 (71.9, 96.2) | 81.7 (44.6, 97.6) | 78.8 (61.4, 91.1) | 97.2 (89.8, 99.8) |
| IMO | OWERRI NORTH | 89.1 (76.9, 96.7) | 89.1 (78.4, 96.1) | 82.0 (49.2, 97.5) | 80.9 (67.4, 90.2) | 97.3 (91.8, 99.7) |
| IMO | OWERRI WEST | 84.8 (67.0, 95.3) | 87.3 (74.9, 95.3) | 82.1 (48.9, 97.4) | 75.8 (59.5, 88.1) | 96.3 (89.1, 99.6) |
| IMO | ONUIMO | 89.0 (73.5, 97.3) | 91.8 (81.3, 97.6) | 73.9 (32.4, 96.6) | 84.7 (69.3, 94.1) | 96.1 (88.1, 99.6) |
| JIGAWA | AUYO | 28.9 (14.0, 47.2) | 94.9 (88.4, 98.0) | 94.8 (86.6, 98.4) | 28.0 (13.4, 45.9) | 95.8 (89.2, 98.7) |
| JIGAWA | BABURA | 43.7 (22.0, 70.3) | 92.0 (79.5, 97.7) | 92.4 (76.3, 99.0) | 40.8 (20.5, 65.1) | 94.9 (83.9, 99.3) |
| JIGAWA | BIRNIWA | 45.8 (23.6, 68.5) | 94.7 (85.2, 98.7) | 93.5 (79.5, 99.1) | 44.6 (23.3, 66.9) | 95.9 (86.3, 99.5) |
| JIGAWA | BIRNIN KUDU | 33.3 (13.2, 56.6) | 85.6 (70.0, 95.5) | 82.5 (60.6, 95.4) | 32.2 (13.1, 54.6) | 86.7 (70.7, 96.7) |
| JIGAWA | BUJI | 42.4 (18.5, 68.7) | 89.5 (73.9, 97.5) | 86.3 (63.2, 97.5) | 41.2 (18.1, 65.8) | 90.8 (74.4, 98.4) |
| JIGAWA | DUTSE | 25.5 (08.2, 51.0) | 87.6 (69.5, 96.8) | 86.0 (65.1, 96.7) | 24.7 (08.1, 49.6) | 88.5 (70.2, 97.3) |
| JIGAWA | GAGARAWA | 44.6 (25.9, 64.0) | 90.2 (80.0, 96.6) | 86.7 (71.7, 96.1) | 43.4 (25.3, 62.8) | 91.3 (81.0, 97.4) |
| JIGAWA | GARKI | 36.5 (16.3, 61.2) | 92.9 (81.0, 98.1) | 92.2 (76.8, 98.7) | 35.1 (15.9, 58.5) | 94.3 (82.3, 99.1) |
| JIGAWA | GUMEL | 43.1 (21.0, 68.2) | 91.5 (77.2, 98.2) | 89.2 (67.7, 98.4) | 41.7 (20.3, 67.0) | 92.8 (78.4, 98.9) |
| JIGAWA | GURI | 22.8 (09.0, 42.8) | 89.6 (77.5, 96.6) | 88.9 (74.4, 96.8) | 21.7 (08.5, 40.8) | 90.7 (79.1, 97.3) |
| JIGAWA | GWARAM | 38.7 (18.8, 60.0) | 89.1 (76.5, 96.0) | 86.9 (70.9, 95.9) | 37.1 (18.2, 57.8) | 90.7 (78.1, 97.1) |
| JIGAWA | GWIWA | 35.1 (15.2, 59.4) | 94.7 (84.8, 99.0) | 94.1 (81.1, 99.3) | 34.2 (14.8, 57.6) | 95.6 (86.0, 99.4) |
| JIGAWA | HADEJIA | 44.3 (21.3, 68.8) | 96.4 (88.3, 99.4) | 97.1 (87.5, 99.7) | 42.7 (20.1, 66.9) | 98.0 (90.9, 99.8) |
| JIGAWA | JAHUN | 36.0 (16.8, 60.3) | 91.5 (79.2, 97.6) | 89.6 (73.3, 97.5) | 35.1 (16.5, 59.1) | 92.4 (80.3, 98.0) |
| JIGAWA | KAFIN HAUSA | 30.1 (13.5, 51.4) | 88.4 (73.1, 96.2) | 86.1 (68.3, 95.9) | 29.1 (13.1, 49.4) | 89.4 (74.0, 97.0) |
| JIGAWA | KAUGAMA | 39.4 (22.9, 57.8) | 95.1 (88.4, 98.4) | 94.2 (85.4, 98.7) | 38.5 (22.4, 56.4) | 96.0 (89.8, 99.1) |
| JIGAWA | KAZAURE | 51.9 (23.9, 79.0) | 93.8 (83.3, 98.7) | 93.2 (77.1, 99.4) | 49.7 (23.3, 75.4) | 96.0 (85.8, 99.7) |
| JIGAWA | KIRI KASAMA | 33.1 (17.6, 52.3) | 92.9 (82.2, 97.8) | 92.7 (79.8, 98.3) | 31.7 (17.0, 50.2) | 94.3 (83.8, 98.8) |
| JIGAWA | KIYAWA | 35.5 (13.0, 65.4) | 87.7 (68.0, 97.3) | 84.6 (58.8, 97.2) | 34.5 (12.5, 62.9) | 88.7 (69.1, 98.1) |
| JIGAWA | MAIGATARI | 41.9 (20.0, 63.7) | 88.4 (76.2, 95.8) | 84.3 (65.1, 94.9) | 40.8 (19.8, 61.8) | 89.5 (77.1, 96.8) |
| JIGAWA | MALAM MADORI | 42.1 (21.5, 64.2) | 96.4 (90.6, 99.0) | 96.6 (88.9, 99.5) | 40.9 (20.9, 62.3) | 97.7 (91.9, 99.6) |
| JIGAWA | MIGA | 35.9 (14.0, 62.5) | 93.5 (82.4, 98.7) | 92.4 (76.6, 99.0) | 35.0 (13.8, 61.6) | 94.4 (83.5, 99.2) |
| JIGAWA | RINGIM | 25.8 (09.0, 49.1) | 90.0 (74.8, 97.5) | 89.0 (70.7, 97.9) | 25.0 (08.7, 47.7) | 90.8 (75.5, 98.3) |
| JIGAWA | RONI | 44.3 (15.4, 75.7) | 93.3 (81.2, 98.9) | 91.6 (69.0, 99.3) | 42.9 (15.3, 73.4) | 94.7 (82.5, 99.5) |
| JIGAWA | SULE TANKARKAR | 35.9 (16.9, 57.8) | 94.2 (86.2, 98.4) | 93.6 (82.8, 98.7) | 34.8 (16.4, 56.3) | 95.3 (87.5, 99.1) |
| JIGAWA | TAURA | 44.0 (23.8, 64.0) | 94.0 (85.1, 98.4) | 92.5 (79.8, 98.6) | 43.0 (23.2, 62.9) | 95.0 (86.2, 99.0) |
| JIGAWA | YANKWASHI | 38.6 (15.7, 67.1) | 95.2 (85.2, 99.1) | 95.0 (81.7, 99.5) | 37.4 (15.4, 64.3) | 96.4 (86.7, 99.7) |
| KADUNA | BIRNIN GWARI | 24.1 (10.3, 44.1) | 78.3 (62.1, 89.1) | 75.0 (56.4, 87.4) | 22.9 (10.1, 41.6) | 79.4 (63.0, 90.4) |
| KADUNA | CHIKUN | 43.1 (23.8, 62.1) | 87.4 (77.8, 94.2) | 85.7 (72.0, 95.1) | 40.1 (22.7, 57.6) | 90.5 (81.0, 96.8) |
| KADUNA | GIWA | 26.3 (11.0, 47.2) | 81.3 (62.4, 93.6) | 79.2 (55.0, 94.0) | 24.4 (09.9, 43.7) | 83.2 (64.3, 95.0) |
| KADUNA | IGABI | 45.3 (28.4, 63.0) | 83.0 (71.9, 91.2) | 79.9 (63.4, 91.4) | 41.3 (26.1, 58.1) | 87.0 (75.8, 94.8) |
| KADUNA | IKARA | 49.7 (27.8, 73.6) | 72.1 (52.4, 86.7) | 54.6 (25.7, 79.4) | 47.2 (26.0, 70.5) | 74.6 (55.0, 89.1) |
| KADUNA | JABA | 77.3 (52.5, 94.6) | 90.7 (77.3, 97.8) | 84.7 (50.7, 98.6) | 72.7 (49.2, 89.9) | 95.4 (84.6, 99.6) |
| KADUNA | JEMAA | 56.0 (32.7, 79.3) | 88.6 (75.5, 96.3) | 86.8 (67.8, 97.0) | 51.7 (30.6, 73.3) | 92.9 (80.6, 98.5) |
| KADUNA | KACHIA | 64.6 (42.0, 84.0) | 90.8 (82.1, 96.4) | 87.3 (69.9, 96.9) | 60.9 (39.5, 80.0) | 94.5 (85.7, 98.8) |
| KADUNA | KADUNA NORTH | 51.8 (30.7, 73.1) | 86.0 (71.9, 94.8) | 86.5 (66.6, 96.8) | 45.9 (27.1, 66.5) | 92.0 (80.1, 98.3) |
| KADUNA | KADUNA SOUTH | 51.8 (30.0, 72.2) | 88.6 (75.1, 96.1) | 91.1 (74.5, 98.6) | 45.7 (26.0, 65.4) | 94.7 (84.5, 99.2) |
| KADUNA | KAGARKO | 76.3 (57.7, 89.8) | 93.1 (85.8, 97.6) | 89.1 (70.4, 98.0) | 72.8 (55.4, 86.2) | 96.6 (90.0, 99.5) |
| KADUNA | KAJURU | 49.4 (17.5, 79.8) | 86.2 (67.2, 96.6) | 81.0 (49.1, 96.4) | 46.8 (16.9, 74.7) | 88.9 (68.8, 98.1) |
| KADUNA | KAURA | 66.9 (36.2, 88.0) | 88.4 (73.4, 96.6) | 84.9 (57.2, 98.1) | 61.6 (33.0, 81.9) | 93.7 (78.7, 99.2) |
| KADUNA | KAURU | 44.2 (24.8, 65.5) | 81.3 (67.8, 91.8) | 74.5 (52.4, 89.3) | 42.2 (23.7, 62.7) | 83.3 (68.8, 93.7) |
| KADUNA | KUBAU | 36.7 (15.7, 62.1) | 80.6 (62.7, 92.6) | 74.4 (49.6, 91.1) | 35.3 (15.0, 59.3) | 82.0 (63.3, 94.0) |
| KADUNA | KUDAN | 66.3 (45.9, 82.7) | 84.2 (70.5, 93.2) | 74.5 (44.5, 93.9) | 61.0 (41.3, 77.5) | 89.5 (75.3, 97.5) |
| KADUNA | LERE | 25.4 (10.8, 47.7) | 81.7 (64.4, 93.3) | 78.4 (59.2, 92.6) | 24.3 (10.5, 44.3) | 82.8 (65.4, 94.2) |
| KADUNA | MAKARFI | 64.0 (36.8, 87.2) | 80.1 (58.3, 93.3) | 62.6 (24.0, 90.9) | 60.0 (34.3, 82.3) | 84.1 (61.3, 96.9) |
| KADUNA | SABON-GARI | 66.9 (46.8, 84.2) | 86.6 (73.4, 95.0) | 85.1 (59.3, 97.7) | 60.0 (41.5, 76.7) | 93.6 (82.0, 99.1) |
| KADUNA | SANGA | 50.6 (27.7, 73.5) | 92.9 (82.5, 98.2) | 91.3 (74.4, 98.7) | 48.7 (26.6, 71.2) | 94.8 (84.6, 99.2) |
| KADUNA | SOBA | 55.7 (28.1, 80.5) | 83.4 (67.6, 93.9) | 75.4 (46.3, 93.4) | 52.2 (26.5, 75.5) | 87.0 (70.4, 96.7) |
| KADUNA | ZANGO KATAF | 69.1 (43.3, 89.0) | 88.8 (77.0, 96.1) | 82.8 (57.6, 96.7) | 64.6 (40.4, 84.3) | 93.3 (82.2, 98.9) |
| KADUNA | ZARIA | 65.9 (43.8, 84.6) | 87.2 (71.2, 95.8) | 86.8 (60.0, 98.5) | 59.1 (38.6, 77.1) | 94.1 (81.5, 99.2) |
| KANO | AJINGI | 17.0 (04.2, 39.3) | 80.7 (58.0, 92.8) | 78.9 (52.2, 92.6) | 16.4 (04.0, 38.7) | 81.4 (59.0, 93.2) |
| KANO | ALBASU | 15.4 (04.3, 36.4) | 80.0 (56.0, 94.2) | 78.2 (51.4, 93.8) | 14.9 (04.2, 35.1) | 80.6 (56.1, 94.4) |
| KANO | BAGWAI | 54.6 (23.6, 82.8) | 85.3 (62.6, 96.9) | 75.7 (39.0, 96.2) | 53.0 (23.0, 80.1) | 86.9 (64.4, 98.2) |
| KANO | BEBEJI | 42.5 (21.1, 66.3) | 63.1 (42.7, 81.5) | 43.6 (19.7, 70.1) | 40.7 (20.1, 63.4) | 65.0 (44.0, 83.7) |
| KANO | BICHI | 50.4 (26.5, 73.6) | 88.3 (70.8, 97.0) | 82.5 (54.0, 96.4) | 49.0 (25.9, 71.7) | 89.6 (72.0, 97.9) |
| KANO | BUNKURE | 32.6 (16.3, 53.2) | 84.1 (70.3, 93.4) | 80.6 (63.7, 92.7) | 31.4 (15.8, 50.7) | 85.4 (71.0, 94.8) |
| KANO | DALA | 49.3 (18.9, 80.9) | 80.3 (55.2, 95.8) | 70.9 (30.1, 95.6) | 46.4 (17.8, 77.1) | 83.2 (57.1, 97.6) |
| KANO | DAMBATTA | 43.7 (14.0, 78.8) | 88.7 (69.6, 97.9) | 85.9 (59.3, 98.4) | 41.7 (13.4, 74.2) | 90.7 (71.5, 99.0) |
| KANO | DAWAKIN KUDU | 28.5 (10.3, 54.8) | 81.7 (59.6, 94.7) | 78.4 (50.6, 94.6) | 27.2 (09.9, 50.3) | 82.9 (60.8, 95.8) |
| KANO | DAWAKIN TOFA | 44.3 (21.7, 70.9) | 81.0 (64.0, 93.1) | 72.4 (46.0, 90.3) | 43.0 (21.1, 67.6) | 82.3 (64.8, 94.4) |
| KANO | DOGUWA | 20.1 (06.4, 40.8) | 81.8 (63.2, 94.1) | 79.0 (57.4, 93.6) | 19.4 (06.2, 39.5) | 82.4 (63.4, 94.7) |
| KANO | FAGGE | 44.6 (11.5, 82.4) | 80.4 (50.5, 97.2) | 73.2 (26.1, 97.7) | 41.8 (10.8, 76.2) | 83.3 (52.1, 98.7) |
| KANO | GABASAWA | 24.4 (05.5, 55.8) | 81.4 (55.2, 94.5) | 78.8 (47.6, 94.9) | 23.3 (05.4, 53.2) | 82.5 (56.5, 95.5) |
| KANO | GARKO | 20.3 (08.3, 37.7) | 75.6 (58.3, 88.0) | 72.5 (51.7, 87.5) | 19.4 (07.8, 37.0) | 76.5 (59.0, 89.3) |
| KANO | GARUN MALLAM | 43.1 (18.2, 70.0) | 80.2 (58.9, 94.7) | 72.0 (41.3, 93.5) | 41.5 (17.8, 67.9) | 81.8 (60.9, 95.7) |
| KANO | GAYA | 16.3 (03.7, 40.4) | 80.2 (55.6, 95.1) | 78.2 (49.3, 95.1) | 15.7 (03.6, 38.8) | 80.8 (56.2, 95.9) |
| KANO | GEZAWA | 31.7 (08.1, 68.1) | 77.3 (48.4, 95.0) | 71.9 (36.2, 95.6) | 30.0 (07.5, 64.1) | 79.0 (49.7, 96.8) |
| KANO | GWALE | 49.4 (17.5, 81.1) | 80.5 (55.1, 96.3) | 71.3 (26.6, 96.4) | 46.5 (16.9, 77.6) | 83.5 (57.6, 98.0) |
| KANO | GWARZO | 57.8 (37.1, 76.3) | 80.3 (66.7, 90.7) | 64.2 (40.7, 84.0) | 56.0 (35.7, 73.8) | 82.2 (68.3, 93.0) |
| KANO | KABO | 56.9 (26.7, 83.0) | 82.9 (60.0, 96.6) | 69.8 (29.9, 95.8) | 55.1 (25.7, 80.2) | 84.6 (61.1, 97.9) |
| KANO | KANO MUNICIPAL | 48.5 (16.4, 82.3) | 81.9 (58.0, 96.3) | 74.3 (30.5, 96.3) | 45.5 (15.4, 77.3) | 84.9 (60.5, 98.1) |
| KANO | KARAYE | 45.9 (19.7, 74.0) | 76.6 (51.3, 93.9) | 64.3 (28.5, 92.1) | 44.4 (18.8, 71.7) | 78.1 (52.5, 95.0) |
| KANO | KIBIYA | 30.6 (15.9, 49.0) | 79.4 (62.5, 90.9) | 75.0 (54.4, 89.8) | 29.4 (15.5, 46.8) | 80.6 (63.3, 92.3) |
| KANO | KIRU | 46.7 (21.8, 73.3) | 68.3 (46.9, 84.7) | 49.1 (17.3, 76.1) | 44.8 (20.6, 69.7) | 70.2 (47.6, 87.5) |
| KANO | KUMBOTSO | 41.7 (15.4, 72.9) | 81.8 (57.0, 95.8) | 75.9 (38.2, 95.6) | 39.4 (14.6, 68.5) | 84.2 (58.1, 97.3) |
| KANO | KUNCHI | 46.1 (18.0, 79.8) | 91.9 (75.2, 98.6) | 89.2 (65.4, 98.7) | 44.8 (17.8, 77.4) | 93.2 (76.4, 99.2) |
| KANO | KURA | 42.2 (21.2, 66.3) | 85.2 (68.5, 95.0) | 80.3 (55.6, 94.7) | 40.5 (19.9, 63.7) | 86.9 (70.3, 96.6) |
| KANO | MADOBI | 50.6 (31.1, 72.4) | 83.4 (65.4, 95.0) | 74.2 (43.5, 94.2) | 48.9 (29.6, 69.9) | 85.1 (67.4, 96.5) |
| KANO | MAKODA | 46.8 (18.1, 78.1) | 90.0 (72.8, 98.2) | 86.6 (60.8, 98.1) | 45.1 (17.6, 75.3) | 91.6 (74.5, 98.9) |
| KANO | MINIJIBIR | 37.0 (11.5, 71.4) | 81.2 (54.7, 95.4) | 75.5 (41.8, 95.2) | 35.5 (11.2, 68.0) | 82.7 (56.3, 96.8) |
| KANO | NASSARAWA | 42.5 (11.0, 78.9) | 81.4 (52.0, 97.1) | 74.5 (29.3, 97.1) | 40.3 (10.6, 73.3) | 83.6 (53.2, 98.4) |
| KANO | RANO | 36.3 (18.6, 58.2) | 76.4 (57.8, 90.5) | 69.0 (44.4, 88.5) | 34.9 (17.7, 56.0) | 77.8 (58.6, 92.1) |
| KANO | RIMIN GADO | 56.3 (26.2, 82.5) | 83.9 (57.4, 96.5) | 72.8 (31.4, 95.8) | 54.3 (25.4, 79.8) | 85.9 (59.9, 98.1) |
| KANO | ROGO | 46.5 (21.6, 73.1) | 75.3 (54.8, 90.9) | 63.5 (32.2, 88.8) | 43.9 (20.8, 68.5) | 78.0 (56.2, 93.4) |
| KANO | SHANONO | 56.2 (33.8, 76.7) | 84.6 (69.8, 94.3) | 73.5 (46.3, 91.3) | 54.5 (33.0, 74.7) | 86.2 (71.5, 96.1) |
| KANO | SUMAILA | 14.2 (03.7, 32.7) | 71.6 (52.2, 86.5) | 68.8 (46.6, 86.0) | 13.7 (03.6, 31.8) | 72.1 (52.6, 86.9) |
| KANO | TAKAI | 17.0 (05.3, 37.2) | 80.3 (57.5, 93.8) | 78.3 (52.0, 93.4) | 16.4 (05.1, 35.5) | 80.8 (58.3, 94.1) |
| KANO | TARAUNI | 42.1 (10.5, 80.4) | 80.8 (50.7, 97.2) | 74.0 (29.2, 97.4) | 39.7 (10.1, 74.9) | 83.2 (52.7, 98.5) |
| KANO | TOFA | 49.5 (21.2, 77.2) | 82.3 (58.9, 95.7) | 72.3 (33.5, 94.4) | 48.0 (20.9, 73.8) | 83.8 (60.3, 96.9) |
| KANO | TSANYAWA | 49.7 (22.9, 77.4) | 87.1 (68.5, 97.1) | 80.4 (49.8, 96.4) | 48.3 (22.3, 75.8) | 88.4 (69.9, 98.2) |
| KANO | TUDUN | 32.6 (16.6, 51.7) | 72.6 (56.8, 86.4) | 64.1 (45.0, 82.6) | 31.4 (16.1, 50.0) | 73.8 (57.3, 87.8) |
| KANO | UNGOGO | 42.6 (14.9, 75.3) | 79.4 (54.7, 94.9) | 71.5 (33.3, 94.6) | 40.3 (14.1, 70.5) | 81.6 (56.5, 96.7) |
| KANO | WARAWA | 28.3 (07.7, 58.8) | 76.4 (47.5, 94.1) | 71.6 (35.7, 94.1) | 26.9 (07.4, 56.0) | 77.8 (48.4, 95.6) |
| KANO | WUDIL | 21.6 (05.1, 49.2) | 75.5 (46.6, 92.8) | 72.0 (37.5, 92.9) | 20.5 (04.9, 47.1) | 76.6 (47.8, 93.9) |
| KATSINA | BAKORI | 28.1 (07.1, 59.7) | 87.5 (64.8, 97.3) | 86.3 (59.1, 98.1) | 26.4 (06.9, 55.4) | 89.1 (67.5, 98.6) |
| KATSINA | BATAGARAWA | 20.9 (09.8, 36.4) | 89.9 (79.5, 96.1) | 89.5 (77.7, 96.5) | 19.9 (09.2, 34.5) | 90.9 (80.3, 97.1) |
| KATSINA | BATSARI | 23.1 (07.6, 48.3) | 95.0 (84.7, 99.0) | 94.8 (82.3, 99.2) | 22.5 (07.3, 46.3) | 95.6 (85.9, 99.3) |
| KATSINA | BAURE | 34.9 (10.3, 67.1) | 93.5 (80.5, 99.0) | 93.4 (75.7, 99.5) | 33.3 (09.8, 64.4) | 95.1 (82.3, 99.6) |
| KATSINA | BINDAWA | 15.9 (04.9, 32.6) | 95.0 (85.2, 99.0) | 95.0 (84.3, 99.2) | 15.5 (04.7, 31.8) | 95.5 (85.8, 99.3) |
| KATSINA | CHARANCHI | 24.0 (09.1, 43.3) | 89.8 (76.8, 96.7) | 88.7 (73.1, 96.6) | 23.4 (09.0, 42.0) | 90.4 (77.1, 97.1) |
| KATSINA | DANDUME | 16.8 (03.6, 41.6) | 90.4 (71.3, 98.4) | 90.4 (69.6, 98.9) | 15.7 (03.4, 39.0) | 91.5 (73.1, 99.0) |
| KATSINA | DANJA | 40.8 (21.1, 64.6) | 82.3 (62.5, 93.9) | 77.9 (52.4, 93.6) | 37.6 (19.2, 60.6) | 85.5 (65.4, 96.3) |
| KATSINA | DAN-MUSA | 33.2 (09.7, 62.3) | 91.1 (73.6, 98.5) | 89.2 (66.1, 98.8) | 32.3 (09.5, 60.6) | 91.9 (74.3, 99.0) |
| KATSINA | DAURA | 09.7 (02.7, 23.9) | 92.7 (81.0, 97.9) | 92.5 (80.5, 98.0) | 09.4 (02.7, 22.6) | 92.9 (81.4, 98.1) |
| KATSINA | DUTSI | 11.7 (03.1, 27.6) | 93.5 (81.9, 98.6) | 93.3 (81.1, 98.7) | 11.4 (03.0, 26.6) | 93.8 (82.3, 98.8) |
| KATSINA | DUTSIN MA | 27.5 (07.7, 57.1) | 88.2 (68.3, 97.2) | 86.4 (60.5, 97.3) | 26.7 (07.5, 55.2) | 89.0 (68.9, 97.9) |
| KATSINA | FASKARI | 17.5 (06.3, 34.6) | 89.5 (75.7, 96.4) | 88.9 (73.1, 96.7) | 16.8 (06.1, 32.9) | 90.2 (76.7, 97.1) |
| KATSINA | FUNTUA | 25.5 (07.0, 51.4) | 90.6 (74.7, 98.1) | 91.3 (70.9, 98.9) | 23.2 (06.1, 47.4) | 92.8 (76.5, 99.1) |
| KATSINA | INGAWA | 24.6 (06.8, 51.8) | 93.9 (80.9, 99.0) | 93.3 (76.5, 99.2) | 24.0 (06.6, 50.7) | 94.6 (81.1, 99.4) |
| KATSINA | JIBIA | 33.3 (12.2, 58.5) | 92.9 (80.1, 98.3) | 92.1 (75.1, 98.8) | 32.1 (11.9, 56.6) | 94.0 (81.3, 99.1) |
| KATSINA | KAFUR | 35.8 (13.9, 63.7) | 78.1 (57.0, 91.8) | 71.7 (42.4, 90.6) | 34.0 (13.4, 61.0) | 79.8 (57.7, 93.1) |
| KATSINA | KAITA | 17.2 (04.0, 40.4) | 92.9 (78.5, 98.6) | 92.7 (76.3, 99.0) | 16.6 (03.9, 38.4) | 93.5 (79.1, 99.2) |
| KATSINA | KANKARA | 28.7 (09.2, 58.7) | 87.5 (67.4, 97.3) | 85.3 (61.7, 97.5) | 27.8 (09.1, 55.0) | 88.4 (68.5, 98.0) |
| KATSINA | KANKIA | 25.1 (11.4, 45.1) | 90.4 (77.9, 97.2) | 88.9 (73.7, 97.1) | 24.5 (11.1, 43.9) | 91.0 (78.5, 97.8) |
| KATSINA | KATSINA | 20.0 (06.6, 39.4) | 90.8 (78.5, 97.2) | 90.9 (77.2, 97.9) | 18.7 (06.1, 37.9) | 92.1 (80.3, 98.2) |
| KATSINA | KURFI | 28.7 (12.4, 51.5) | 92.8 (79.5, 98.2) | 91.9 (74.8, 98.6) | 27.9 (12.2, 50.6) | 93.5 (80.0, 98.9) |
| KATSINA | KUSADA | 33.4 (09.7, 66.6) | 91.6 (74.4, 98.7) | 89.9 (65.7, 98.8) | 32.6 (09.4, 65.0) | 92.5 (74.8, 99.2) |
| KATSINA | MAI'ADUA | 10.0 (02.7, 24.3) | 92.4 (80.2, 98.0) | 92.2 (79.0, 98.1) | 09.7 (02.6, 23.6) | 92.7 (80.2, 98.1) |
| KATSINA | MALUMFASHI | 37.8 (11.6, 72.2) | 83.0 (57.2, 96.2) | 78.0 (42.7, 96.0) | 36.2 (11.3, 69.8) | 84.7 (58.4, 97.6) |
| KATSINA | MANI | 12.7 (03.4, 28.5) | 93.9 (83.2, 98.8) | 93.8 (82.3, 99.1) | 12.3 (03.3, 27.0) | 94.3 (83.4, 99.1) |
| KATSINA | MASHI | 10.1 (02.7, 24.1) | 95.0 (85.6, 98.9) | 95.0 (85.2, 99.1) | 09.7 (02.6, 23.5) | 95.3 (85.7, 99.2) |
| KATSINA | MATAZU | 34.7 (10.0, 67.5) | 88.1 (64.8, 98.0) | 85.0 (54.0, 98.0) | 33.8 (09.8, 66.0) | 88.9 (65.2, 98.6) |
| KATSINA | MUSAWA | 44.0 (19.1, 72.2) | 87.6 (70.0, 97.4) | 82.7 (56.5, 97.0) | 42.9 (18.8, 69.9) | 88.7 (70.5, 98.1) |
| KATSINA | RIMI | 20.8 (08.7, 38.2) | 90.5 (79.5, 97.1) | 90.0 (77.3, 97.3) | 20.0 (08.4, 36.7) | 91.4 (80.5, 97.6) |
| KATSINA | SABUWA | 11.1 (02.0, 29.8) | 82.2 (55.0, 96.3) | 81.3 (51.6, 96.5) | 10.6 (01.8, 28.5) | 82.7 (55.2, 96.8) |
| KATSINA | SAFANA | 26.0 (08.4, 49.8) | 93.1 (80.2, 98.7) | 92.2 (75.7, 98.9) | 25.4 (08.4, 48.8) | 93.7 (80.6, 99.1) |
| KATSINA | SANDAMU | 14.7 (04.3, 33.1) | 95.3 (85.1, 99.1) | 95.2 (83.9, 99.2) | 14.3 (04.1, 31.8) | 95.6 (85.5, 99.2) |
| KATSINA | ZANGO | 15.7 (04.2, 36.0) | 94.4 (82.0, 99.0) | 94.4 (81.6, 99.3) | 15.2 (03.9, 34.3) | 95.0 (82.6, 99.4) |
| KEBBI | ALIERO | 07.4 (01.5, 19.4) | 93.5 (78.7, 99.0) | 93.4 (78.0, 99.1) | 07.2 (01.5, 18.4) | 93.7 (78.9, 99.2) |
| KEBBI | AREWA DANDI | 26.9 (09.6, 51.5) | 83.2 (67.4, 93.7) | 79.9 (60.8, 93.1) | 26.0 (09.4, 50.3) | 84.1 (68.2, 94.8) |
| KEBBI | ARGUNGU | 19.2 (05.9, 40.1) | 88.7 (70.7, 97.7) | 87.6 (66.5, 98.0) | 18.7 (05.7, 39.2) | 89.2 (70.8, 98.2) |
| KEBBI | AUGIE | 25.9 (13.0, 44.1) | 89.6 (78.8, 96.6) | 88.2 (75.6, 96.5) | 25.2 (12.5, 43.4) | 90.2 (79.3, 97.1) |
| KEBBI | BAGUDO | 40.0 (18.5, 65.8) | 85.3 (69.8, 94.2) | 80.9 (59.7, 93.8) | 38.3 (18.2, 62.7) | 87.0 (71.6, 95.9) |
| KEBBI | BIRNIN KEBBI | 09.1 (02.8, 19.7) | 78.3 (62.4, 89.5) | 76.9 (60.1, 89.3) | 08.8 (02.8, 19.3) | 78.5 (62.8, 89.9) |
| KEBBI | BUNZA | 28.2 (10.5, 50.2) | 82.8 (64.1, 94.6) | 79.2 (55.0, 94.0) | 27.4 (10.3, 49.3) | 83.5 (64.5, 95.1) |
| KEBBI | DANDI | 47.1 (21.3, 73.5) | 85.1 (68.7, 95.6) | 78.8 (53.2, 94.6) | 45.3 (20.3, 69.2) | 86.9 (70.0, 97.0) |
| KEBBI | WASAGU/DANKO | 40.2 (14.6, 67.7) | 80.5 (61.5, 92.6) | 74.1 (47.7, 91.8) | 38.7 (14.3, 66.0) | 82.0 (62.5, 94.0) |
| KEBBI | PAKAL | 39.3 (15.3, 64.2) | 80.5 (60.2, 93.3) | 73.6 (45.6, 92.2) | 37.8 (15.0, 62.2) | 82.0 (61.1, 94.9) |
| KEBBI | GWANDU | 18.5 (06.5, 38.3) | 95.0 (84.6, 99.0) | 94.8 (83.8, 99.2) | 18.0 (06.4, 37.7) | 95.5 (85.5, 99.3) |
| KEBBI | JEGA | 12.4 (03.1, 29.0) | 86.8 (67.8, 97.0) | 86.0 (64.7, 97.1) | 11.9 (03.0, 28.0) | 87.2 (67.9, 97.3) |
| KEBBI | KALGO | 12.3 (03.0, 29.4) | 78.4 (58.2, 92.4) | 76.6 (54.1, 92.1) | 12.0 (02.9, 28.2) | 78.7 (58.3, 92.6) |
| KEBBI | KOKO/BESSE | 28.9 (12.4, 50.2) | 64.1 (45.4, 80.8) | 55.3 (31.7, 77.1) | 27.1 (11.6, 47.6) | 66.0 (46.8, 83.0) |
| KEBBI | MAIYAMA | 21.8 (09.9, 38.8) | 82.9 (67.3, 93.4) | 80.8 (62.5, 93.0) | 21.0 (09.3, 37.1) | 83.8 (68.2, 94.2) |
| KEBBI | NGASKI | 25.6 (11.7, 45.8) | 94.2 (85.0, 98.2) | 93.7 (82.6, 98.5) | 24.9 (11.3, 44.7) | 94.9 (85.7, 98.8) |
| KEBBI | SAKABA | 64.1 (35.3, 87.9) | 89.5 (73.3, 97.5) | 81.0 (47.8, 97.4) | 62.0 (34.2, 85.3) | 91.5 (75.1, 99.1) |
| KEBBI | SHANGA | 26.8 (11.6, 47.6) | 71.9 (52.3, 87.1) | 65.8 (42.0, 85.1) | 25.6 (11.2, 45.4) | 73.0 (53.5, 88.6) |
| KEBBI | SURU | 31.6 (10.9, 59.2) | 81.5 (59.8, 94.6) | 77.0 (48.9, 94.3) | 30.6 (10.6, 56.7) | 82.6 (60.4, 95.5) |
| KEBBI | YAURI | 28.4 (14.1, 47.1) | 91.6 (81.9, 97.3) | 90.5 (76.8, 97.5) | 27.6 (13.7, 45.6) | 92.4 (82.4, 98.0) |
| KEBBI | ZURU | 39.6 (17.3, 66.6) | 89.2 (74.4, 96.9) | 86.6 (66.0, 97.0) | 38.6 (16.8, 63.9) | 90.3 (75.4, 97.8) |
| KOGI | ADAVI | 47.5 (24.1, 70.8) | 90.5 (77.0, 97.4) | 89.0 (71.3, 97.9) | 45.2 (23.9, 66.4) | 92.8 (81.1, 98.7) |
| KOGI | AJAOKUTA | 41.6 (18.7, 67.7) | 91.2 (76.6, 98.2) | 89.0 (65.9, 98.4) | 40.2 (17.6, 65.4) | 92.6 (78.0, 98.9) |
| KOGI | ANKPA | 65.9 (48.6, 81.0) | 90.1 (80.6, 96.7) | 86.4 (65.7, 97.2) | 62.0 (45.8, 76.5) | 94.1 (85.4, 98.8) |
| KOGI | BASSA | 45.9 (19.5, 73.8) | 86.9 (69.1, 96.3) | 82.2 (58.8, 96.5) | 44.0 (18.9, 71.4) | 88.8 (70.2, 97.9) |
| KOGI | DEKINA | 57.1 (33.3, 78.4) | 91.3 (79.7, 97.5) | 89.3 (68.5, 98.5) | 54.0 (31.7, 74.2) | 94.3 (82.8, 99.2) |
| KOGI | IBAJI | 69.1 (44.2, 88.9) | 92.4 (82.3, 97.8) | 88.4 (66.9, 98.6) | 66.0 (41.7, 85.3) | 95.5 (85.5, 99.5) |
| KOGI | IDAH | 65.3 (40.7, 84.6) | 91.8 (79.4, 98.0) | 88.2 (64.4, 98.3) | 62.5 (38.5, 82.0) | 94.7 (84.1, 99.4) |
| KOGI | IGALAMELLA | 65.9 (47.2, 81.9) | 92.2 (83.6, 96.9) | 88.6 (70.6, 97.3) | 63.0 (45.7, 79.0) | 95.1 (86.8, 98.9) |
| KOGI | IJUMU | 78.5 (60.3, 91.1) | 90.6 (79.6, 97.0) | 91.2 (70.8, 99.1) | 71.7 (54.3, 85.2) | 97.4 (90.3, 99.8) |
| KOGI | KABBA/BUNU | 69.1 (47.5, 87.5) | 88.7 (78.2, 96.0) | 89.0 (68.6, 98.2) | 62.3 (42.7, 80.5) | 95.5 (87.1, 99.3) |
| KOGI | KOGI | 39.8 (14.5, 71.7) | 82.8 (63.5, 95.1) | 76.9 (47.7, 94.7) | 38.4 (14.1, 68.5) | 84.2 (64.3, 96.5) |
| KOGI | LOKOJA | 43.1 (19.9, 67.0) | 77.8 (57.5, 92.3) | 69.1 (41.5, 90.7) | 41.0 (19.5, 63.9) | 79.8 (60.0, 94.4) |
| KOGI | MOPAMURO | 80.8 (52.6, 95.6) | 92.5 (81.2, 98.2) | 89.6 (62.5, 99.2) | 76.0 (49.9, 92.0) | 97.3 (88.7, 99.8) |
| KOGI | OFU | 61.2 (35.9, 82.2) | 92.6 (81.8, 97.8) | 90.1 (69.2, 98.5) | 58.5 (34.7, 79.3) | 95.2 (84.4, 99.3) |
| KOGI | OGORI MAGOGO | 64.4 (39.9, 84.8) | 95.7 (86.0, 99.1) | 95.0 (77.8, 99.6) | 62.5 (37.8, 83.1) | 97.6 (89.4, 99.9) |
| KOGI | OKEHI | 53.1 (27.7, 77.4) | 92.4 (79.8, 98.3) | 92.7 (76.3, 99.1) | 49.8 (25.9, 72.8) | 95.7 (84.5, 99.6) |
| KOGI | OKENE | 52.8 (33.5, 72.6) | 95.4 (88.2, 98.8) | 95.4 (84.5, 99.4) | 51.0 (31.9, 69.6) | 97.2 (90.3, 99.7) |
| KOGI | OLAMABORO | 61.8 (40.3, 81.0) | 92.2 (81.2, 97.5) | 90.0 (68.8, 98.7) | 58.5 (38.5, 76.3) | 95.4 (85.8, 99.4) |
| KOGI | OMALA | 51.4 (31.5, 70.8) | 89.3 (79.3, 95.9) | 85.2 (68.7, 95.4) | 49.4 (30.4, 68.0) | 91.3 (81.3, 97.4) |
| KOGI | YAGBA EAST | 76.0 (50.2, 91.7) | 92.0 (81.6, 97.4) | 88.7 (67.1, 98.3) | 71.6 (47.5, 87.6) | 96.5 (88.0, 99.6) |
| KOGI | YAGBA WEST | 79.4 (53.4, 95.9) | 92.8 (80.5, 98.5) | 87.9 (56.3, 99.0) | 75.5 (49.9, 92.6) | 96.7 (86.9, 99.8) |
| KWARA | ASA | 65.2 (45.6, 81.8) | 87.9 (77.7, 94.5) | 84.4 (65.4, 95.5) | 59.8 (41.4, 76.0) | 93.3 (83.8, 98.3) |
| KWARA | BARUTEN | 64.3 (43.0, 82.6) | 89.2 (79.8, 95.0) | 84.0 (66.6, 94.6) | 60.6 (40.4, 77.4) | 92.9 (83.8, 97.7) |
| KWARA | EDU | 40.7 (22.8, 61.8) | 90.2 (78.2, 96.7) | 87.5 (70.8, 96.9) | 39.4 (22.2, 59.9) | 91.5 (78.7, 97.9) |
| KWARA | EKITI | 87.6 (73.5, 96.2) | 94.3 (86.7, 98.6) | 85.4 (51.2, 98.9) | 84.5 (70.7, 93.9) | 97.5 (91.8, 99.8) |
| KWARA | IFELODUN | 67.8 (48.5, 83.6) | 93.1 (86.9, 97.1) | 91.0 (79.7, 97.6) | 64.8 (46.5, 79.7) | 96.1 (90.8, 99.0) |
| KWARA | ILORIN-EAST | 65.3 (47.7, 81.7) | 89.3 (80.0, 95.4) | 87.7 (70.8, 96.8) | 60.2 (43.0, 76.2) | 94.4 (85.8, 98.7) |
| KWARA | ILORIN-SOUTH | 63.6 (43.0, 82.4) | 89.4 (77.8, 96.1) | 87.0 (66.3, 97.1) | 59.2 (39.4, 78.3) | 93.8 (82.7, 98.9) |
| KWARA | ILORIN-WEST | 63.1 (42.7, 78.8) | 88.0 (77.1, 95.3) | 87.8 (69.3, 96.9) | 57.0 (39.1, 72.8) | 94.1 (85.5, 98.6) |
| KWARA | IREPODUN | 79.2 (63.7, 90.3) | 93.6 (86.2, 97.8) | 91.0 (70.3, 98.8) | 75.3 (59.9, 86.4) | 97.5 (92.1, 99.7) |
| KWARA | OSIN | 82.1 (62.4, 94.3) | 94.7 (86.7, 98.7) | 91.6 (69.5, 99.3) | 78.9 (59.8, 91.7) | 97.9 (92.2, 99.9) |
| KWARA | KAIAMA | 64.5 (43.2, 83.1) | 89.0 (77.9, 95.6) | 83.6 (63.4, 95.3) | 61.2 (41.9, 78.9) | 92.4 (81.4, 97.9) |
| KWARA | MORO | 63.9 (42.4, 80.7) | 88.5 (78.3, 95.1) | 82.4 (59.9, 94.7) | 60.5 (39.6, 76.7) | 91.9 (82.4, 97.8) |
| KWARA | OFFA | 69.4 (46.5, 86.9) | 89.5 (76.5, 97.0) | 92.2 (73.3, 99.3) | 62.2 (41.0, 80.0) | 96.7 (88.5, 99.7) |
| KWARA | OKE ERO | 88.2 (77.0, 95.8) | 95.3 (88.8, 98.8) | 88.5 (62.4, 99.0) | 85.5 (74.3, 93.5) | 98.1 (93.4, 99.8) |
| KWARA | OYUN | 70.2 (51.0, 84.7) | 90.1 (79.8, 96.1) | 90.3 (70.8, 98.8) | 64.1 (46.6, 77.7) | 96.2 (88.6, 99.6) |
| KWARA | PATEGI | 44.1 (20.7, 68.6) | 93.8 (84.1, 98.5) | 92.9 (78.6, 98.9) | 42.5 (19.8, 66.3) | 95.4 (85.2, 99.3) |
| LAGOS | AGEGE | 85.5 (70.9, 94.7) | 92.0 (82.0, 97.6) | 87.0 (58.3, 98.8) | 80.1 (63.7, 91.5) | 97.5 (91.3, 99.9) |
| LAGOS | AJEROMI/IFELODUN | 59.4 (38.8, 78.4) | 93.8 (84.8, 98.3) | 94.1 (79.3, 99.6) | 56.1 (35.9, 75.6) | 97.1 (89.3, 99.8) |
| LAGOS | ALIMOSHO | 80.9 (67.5, 90.4) | 89.0 (79.5, 95.3) | 92.8 (76.0, 99.1) | 71.8 (58.4, 82.9) | 98.1 (93.1, 99.8) |
| LAGOS | AMUWO-ODOFIN | 68.3 (51.8, 82.4) | 92.2 (83.0, 97.2) | 94.2 (79.4, 99.4) | 62.9 (46.4, 77.7) | 97.6 (92.0, 99.8) |
| LAGOS | APAPA | 53.6 (33.5, 73.7) | 95.6 (89.1, 98.7) | 97.7 (90.5, 99.8) | 50.6 (31.1, 70.8) | 98.6 (93.9, 99.9) |
| LAGOS | BADAGRY | 66.6 (40.7, 86.9) | 87.3 (71.7, 96.0) | 85.7 (57.8, 98.0) | 59.9 (36.8, 80.1) | 94.0 (81.8, 99.3) |
| LAGOS | EPE | 67.1 (42.5, 87.4) | 89.9 (78.2, 96.7) | 85.9 (61.9, 97.7) | 63.0 (39.6, 83.5) | 94.0 (83.3, 99.0) |
| LAGOS | ETI-OSA | 64.6 (41.7, 82.4) | 93.1 (81.9, 98.1) | 93.5 (74.9, 99.4) | 60.5 (39.5, 78.4) | 97.2 (89.8, 99.8) |
| LAGOS | IBEJU/LEKKI | 68.8 (40.3, 90.7) | 89.7 (75.7, 97.6) | 85.3 (57.0, 98.0) | 64.4 (36.9, 85.8) | 94.2 (80.7, 99.4) |
| LAGOS | IFAKO/IJAIYE | 86.1 (73.5, 94.0) | 90.6 (81.2, 96.4) | 86.6 (63.7, 98.4) | 79.0 (64.7, 89.0) | 97.7 (92.6, 99.7) |
| LAGOS | IKEJA | 80.7 (67.2, 90.4) | 92.8 (85.9, 97.0) | 91.6 (74.7, 99.1) | 75.4 (61.2, 85.4) | 98.0 (93.5, 99.8) |
| LAGOS | IKORODU | 78.4 (61.0, 90.7) | 90.5 (79.9, 96.5) | 89.4 (66.9, 98.1) | 71.9 (55.6, 85.4) | 97.0 (90.4, 99.6) |
| LAGOS | KOSOFE | 74.1 (56.7, 86.6) | 92.1 (83.3, 97.2) | 88.6 (65.4, 99.1) | 69.8 (53.4, 82.8) | 96.4 (88.4, 99.7) |
| LAGOS | LAGOS ISLAND | 59.4 (45.1, 72.7) | 95.2 (90.0, 98.2) | 96.0 (88.0, 99.4) | 56.4 (42.6, 70.3) | 98.2 (94.2, 99.7) |
| LAGOS | LAGOS MAINLAND | 63.3 (46.2, 77.7) | 95.4 (89.4, 98.5) | 98.5 (93.7, 99.8) | 59.5 (42.8, 74.0) | 99.2 (96.8, 99.9) |
| LAGOS | MUSHIN | 66.8 (46.6, 81.4) | 94.6 (89.2, 98.0) | 96.7 (89.5, 99.6) | 62.9 (43.6, 78.4) | 98.6 (95.1, 99.9) |
| LAGOS | OJO | 79.3 (56.7, 93.3) | 88.8 (73.8, 96.9) | 88.8 (61.5, 99.3) | 71.0 (49.5, 86.9) | 97.1 (89.2, 99.8) |
| LAGOS | OSHODI/ISOLO | 72.5 (52.8, 86.4) | 92.6 (85.3, 97.1) | 92.7 (76.0, 99.4) | 67.8 (49.5, 81.2) | 97.3 (90.6, 99.8) |
| LAGOS | SHOMOLU | 69.8 (51.5, 83.8) | 94.4 (87.9, 98.1) | 95.4 (85.4, 99.6) | 65.8 (47.4, 79.8) | 98.4 (94.2, 99.9) |
| LAGOS | SURULERE | 59.9 (43.8, 75.7) | 94.8 (89.0, 98.0) | 96.1 (87.7, 99.5) | 56.6 (41.3, 72.4) | 98.2 (93.8, 99.8) |
| NASSARAWA | AKWANGA | 63.2 (42.3, 80.5) | 95.1 (87.0, 98.8) | 92.8 (75.0, 99.2) | 61.6 (40.8, 78.3) | 96.7 (89.2, 99.6) |
| NASSARAWA | AWE | 59.1 (23.9, 87.8) | 90.2 (75.2, 97.6) | 85.2 (59.7, 97.8) | 56.5 (22.5, 84.0) | 92.8 (77.7, 99.2) |
| NASSARAWA | DOMA | 45.0 (22.6, 70.2) | 90.6 (79.4, 96.7) | 88.3 (71.2, 97.3) | 43.0 (21.7, 66.4) | 92.6 (80.7, 98.4) |
| NASSARAWA | KARU | 61.4 (43.1, 77.6) | 90.4 (81.3, 96.1) | 88.9 (72.3, 97.1) | 57.1 (40.6, 72.6) | 94.7 (85.9, 98.7) |
| NASSARAWA | KEANA | 53.0 (22.7, 83.2) | 81.1 (61.5, 93.8) | 73.9 (39.9, 93.8) | 48.2 (20.4, 77.4) | 85.9 (66.1, 97.0) |
| NASSARAWA | KEFFI | 75.3 (52.9, 91.3) | 90.4 (78.5, 96.9) | 92.3 (69.4, 99.4) | 68.3 (46.4, 85.5) | 97.4 (89.0, 99.8) |
| NASSARAWA | KOKONA | 65.6 (46.7, 81.8) | 93.7 (86.7, 97.8) | 93.6 (80.6, 99.0) | 62.2 (43.7, 78.3) | 97.1 (91.4, 99.6) |
| NASSARAWA | LAFIA | 70.1 (47.5, 88.2) | 91.5 (81.1, 97.4) | 85.1 (58.5, 97.5) | 67.2 (45.1, 85.1) | 94.4 (84.0, 99.1) |
| NASSARAWA | NASARAWA | 51.0 (31.1, 70.2) | 90.0 (80.3, 96.0) | 86.4 (71.2, 95.7) | 49.0 (30.0, 67.2) | 92.0 (82.4, 97.7) |
| NASSARAWA | NASSARAWA-EGGON | 68.1 (42.6, 86.9) | 93.6 (82.6, 98.4) | 89.0 (67.1, 98.6) | 66.1 (41.0, 85.0) | 95.6 (84.5, 99.5) |
| NASSARAWA | OBI | 62.2 (29.9, 88.8) | 85.8 (67.7, 95.8) | 77.7 (44.6, 96.3) | 58.2 (28.9, 83.8) | 89.8 (72.5, 98.6) |
| NASSARAWA | TOTO | 49.7 (26.4, 73.0) | 91.2 (79.7, 97.4) | 87.8 (69.2, 97.2) | 48.2 (25.6, 70.4) | 92.6 (81.0, 98.5) |
| NASSARAWA | WAMBA | 67.4 (47.0, 83.6) | 93.7 (85.5, 98.2) | 88.9 (71.5, 98.1) | 65.8 (46.1, 82.0) | 95.3 (86.6, 99.3) |
| NIGER | AGAIE | 41.7 (20.9, 64.5) | 91.4 (79.9, 97.3) | 89.1 (72.8, 97.6) | 40.6 (20.5, 62.5) | 92.6 (80.6, 98.3) |
| NIGER | AGWARA | 24.1 (09.4, 44.4) | 95.2 (86.5, 98.9) | 95.1 (84.2, 99.2) | 23.4 (09.1, 43.2) | 95.9 (87.4, 99.3) |
| NIGER | BIDA | 37.4 (06.1, 79.7) | 91.1 (68.8, 98.9) | 90.2 (58.5, 99.5) | 35.4 (06.0, 76.0) | 93.1 (71.5, 99.7) |
| NIGER | BORGU | 38.9 (21.8, 57.9) | 92.4 (84.6, 96.9) | 91.2 (81.3, 97.1) | 37.4 (21.3, 55.3) | 93.9 (85.9, 98.1) |
| NIGER | BOSSO | 43.1 (20.7, 68.0) | 91.4 (79.6, 97.7) | 89.1 (72.4, 97.9) | 41.7 (20.1, 65.4) | 92.8 (80.9, 98.7) |
| NIGER | CHANCHAGA | 45.8 (19.4, 73.8) | 92.5 (77.8, 98.6) | 91.4 (70.4, 99.2) | 43.9 (18.7, 71.5) | 94.4 (79.9, 99.5) |
| NIGER | EDATI | 21.1 (06.7, 44.7) | 93.5 (81.7, 98.7) | 93.2 (79.7, 99.0) | 20.4 (06.6, 43.6) | 94.2 (82.4, 99.1) |
| NIGER | GBAKO | 32.3 (11.0, 61.9) | 89.6 (72.6, 97.4) | 87.4 (65.0, 97.8) | 31.1 (10.9, 59.2) | 90.7 (73.7, 98.3) |
| NIGER | GURARA | 62.4 (40.5, 81.4) | 93.5 (82.2, 98.4) | 90.4 (70.6, 98.7) | 60.7 (40.0, 78.2) | 95.2 (84.3, 99.3) |
| NIGER | KATCHA | 39.4 (15.5, 66.3) | 91.2 (77.8, 97.8) | 89.0 (70.6, 97.8) | 38.2 (15.1, 63.9) | 92.4 (78.8, 98.6) |
| NIGER | KONTAGORA | 49.4 (15.6, 82.3) | 91.3 (76.7, 98.2) | 89.5 (65.2, 98.9) | 47.1 (15.2, 77.5) | 93.6 (79.2, 99.4) |
| NIGER | LAPAI | 46.3 (26.4, 66.8) | 90.0 (78.6, 96.1) | 86.3 (69.5, 95.7) | 45.1 (25.9, 65.1) | 91.2 (80.3, 97.2) |
| NIGER | LAVUN | 31.7 (12.4, 57.8) | 90.5 (77.8, 96.8) | 89.1 (73.3, 96.9) | 30.5 (12.0, 55.0) | 91.6 (78.7, 97.6) |
| NIGER | MGAMA | 28.5 (10.3, 53.3) | 91.9 (80.3, 98.0) | 91.0 (76.9, 98.4) | 27.5 (09.9, 51.0) | 93.0 (81.2, 98.8) |
| NIGER | MARIGA | 57.6 (32.7, 80.8) | 89.0 (77.3, 95.8) | 83.5 (63.9, 95.4) | 55.1 (31.4, 77.9) | 91.5 (79.8, 97.8) |
| NIGER | MASHEGU | 37.3 (19.3, 58.2) | 86.4 (75.9, 94.0) | 83.1 (67.5, 93.7) | 35.8 (18.6, 55.4) | 87.9 (77.1, 95.3) |
| NIGER | MOKWA | 26.6 (12.9, 47.1) | 75.2 (58.8, 88.2) | 69.8 (50.7, 86.6) | 25.6 (12.2, 45.4) | 76.2 (59.6, 89.6) |
| NIGER | MUYA | 32.5 (10.4, 62.3) | 86.7 (69.0, 96.3) | 84.3 (61.9, 96.7) | 31.1 (10.2, 59.9) | 88.1 (70.5, 97.5) |
| NIGER | PAIKORO | 39.3 (19.5, 62.8) | 88.8 (74.9, 96.6) | 86.1 (65.0, 96.9) | 37.9 (18.8, 60.4) | 90.3 (76.0, 97.8) |
| NIGER | RAFI | 63.6 (41.3, 81.9) | 89.7 (78.9, 95.8) | 86.3 (67.2, 96.5) | 59.7 (39.0, 78.1) | 93.6 (83.0, 98.6) |
| NIGER | RIJAU | 47.6 (20.0, 74.9) | 86.2 (69.6, 95.0) | 80.5 (54.6, 95.0) | 45.9 (19.4, 72.0) | 87.9 (70.8, 96.6) |
| NIGER | SHIRORO | 36.8 (17.8, 59.6) | 86.7 (73.3, 94.5) | 83.7 (65.0, 94.2) | 35.2 (17.1, 56.4) | 88.3 (74.7, 95.9) |
| NIGER | SULEJA | 71.1 (45.1, 88.8) | 95.6 (86.3, 99.3) | 93.5 (74.0, 99.6) | 69.2 (43.9, 86.8) | 97.5 (90.1, 99.9) |
| NIGER | TAFA | 75.0 (53.8, 89.7) | 95.9 (88.5, 99.2) | 93.3 (73.8, 99.5) | 73.1 (52.5, 87.8) | 97.7 (91.3, 99.9) |
| NIGER | WUSHISHI | 63.5 (33.2, 88.1) | 91.3 (77.7, 97.8) | 86.5 (59.0, 98.0) | 60.9 (32.1, 85.1) | 93.9 (80.1, 99.2) |
| OGUN | ABEOKUTA SOUTH | 77.1 (50.4, 93.8) | 77.2 (54.3, 92.2) | 70.1 (27.4, 94.6) | 62.2 (37.6, 83.1) | 92.1 (73.9, 99.3) |
| OGUN | ABEOKUTA NORTH | 64.0 (40.4, 82.6) | 72.7 (55.9, 86.2) | 66.4 (33.5, 88.1) | 51.4 (31.3, 70.3) | 85.2 (70.4, 94.9) |
| OGUN | ADO ODO/OTA | 78.7 (60.3, 91.5) | 82.0 (66.7, 91.4) | 82.7 (60.2, 95.7) | 66.0 (47.3, 80.5) | 94.7 (86.6, 98.8) |
| OGUN | EGBADO NORTH | 50.9 (28.3, 73.1) | 71.7 (55.6, 85.1) | 63.3 (37.6, 84.2) | 42.9 (23.1, 61.7) | 79.7 (63.4, 91.9) |
| OGUN | EGBADO SOUTH | 63.8 (39.8, 84.4) | 71.8 (54.4, 86.3) | 58.7 (30.6, 83.9) | 53.2 (30.8, 73.2) | 82.5 (66.5, 94.3) |
| OGUN | EWEKORO | 63.9 (37.6, 83.7) | 81.1 (63.3, 92.3) | 73.7 (40.5, 91.7) | 56.7 (33.6, 76.7) | 88.3 (72.9, 96.8) |
| OGUN | IFO | 80.9 (66.5, 90.7) | 86.8 (75.4, 93.8) | 87.5 (67.6, 97.2) | 71.2 (57.9, 83.2) | 96.5 (90.8, 99.4) |
| OGUN | IJEBU NORTH EAST | 48.8 (26.6, 71.2) | 84.9 (69.6, 94.6) | 81.6 (56.0, 95.4) | 45.1 (24.4, 66.0) | 88.7 (73.0, 97.3) |
| OGUN | IJEBU EAST | 53.7 (32.4, 73.0) | 86.3 (74.1, 93.9) | 81.9 (59.8, 94.2) | 50.0 (30.3, 68.1) | 90.0 (77.2, 96.7) |
| OGUN | IJEBU NORTH | 44.6 (22.8, 67.5) | 86.9 (72.0, 95.3) | 84.7 (62.5, 96.4) | 41.5 (21.3, 63.1) | 90.0 (75.0, 97.8) |
| OGUN | IJEBU ODE | 58.8 (38.6, 77.6) | 85.2 (72.5, 93.4) | 81.4 (61.7, 93.2) | 53.4 (34.7, 71.2) | 90.6 (80.1, 97.1) |
| OGUN | IKENNE | 59.0 (33.6, 81.4) | 91.2 (78.9, 97.6) | 91.5 (72.6, 99.0) | 54.6 (31.1, 76.8) | 95.6 (84.6, 99.5) |
| OGUN | IMEKO AFON | 47.8 (21.5, 73.4) | 79.0 (62.9, 90.5) | 76.0 (50.8, 92.4) | 41.2 (18.7, 63.7) | 85.6 (70.2, 95.5) |
| OGUN | IPOKIA | 56.7 (24.2, 86.6) | 79.1 (57.7, 92.7) | 70.8 (37.2, 92.9) | 50.2 (20.8, 78.9) | 85.5 (64.7, 96.9) |
| OGUN | OBAFEMI OWODE | 70.8 (51.1, 86.0) | 83.4 (70.4, 92.8) | 80.6 (56.2, 95.1) | 60.9 (42.8, 75.9) | 93.4 (82.6, 98.7) |
| OGUN | ODEDA | 68.0 (48.4, 84.8) | 82.6 (70.0, 92.4) | 79.2 (53.6, 94.9) | 58.7 (40.2, 75.3) | 91.9 (80.3, 98.2) |
| OGUN | ODOGBOLU | 61.3 (42.2, 78.4) | 90.1 (80.1, 96.3) | 88.3 (69.4, 97.1) | 57.3 (39.4, 73.4) | 94.2 (84.7, 98.6) |
| OGUN | OGUN WATERSIDE | 39.4 (17.8, 63.0) | 85.9 (69.1, 95.2) | 81.9 (56.3, 94.7) | 37.3 (17.0, 59.5) | 88.1 (70.3, 96.8) |
| OGUN | REMO NORTH | 56.3 (30.0, 80.1) | 90.4 (78.1, 97.4) | 91.7 (74.8, 99.0) | 51.4 (27.5, 74.2) | 95.4 (84.3, 99.5) |
| OGUN | SAGAMU | 71.6 (51.3, 87.7) | 89.6 (78.7, 95.9) | 88.6 (68.7, 98.1) | 65.5 (46.4, 80.9) | 95.7 (87.2, 99.4) |
| ONDO | AKOKO NORTH EAST | 86.1 (70.0, 95.3) | 94.2 (85.7, 98.6) | 90.8 (65.6, 99.4) | 82.2 (66.1, 92.1) | 98.1 (92.5, 99.9) |
| ONDO | AKOKO SOUTH EAST | 87.3 (70.7, 96.3) | 95.6 (88.0, 99.2) | 91.1 (60.7, 99.6) | 84.6 (68.5, 94.4) | 98.3 (92.5, 99.9) |
| ONDO | AKOKO SOUTH WEST | 85.4 (67.7, 95.9) | 95.1 (86.5, 98.9) | 90.5 (66.7, 99.4) | 82.5 (65.1, 93.6) | 98.0 (91.9, 99.9) |
| ONDO | AKOKO NORTH WEST | 82.2 (63.2, 93.6) | 94.1 (85.6, 98.4) | 91.6 (71.6, 99.2) | 78.5 (60.3, 90.6) | 97.9 (92.1, 99.8) |
| ONDO | AKURE NORTH | 83.5 (66.0, 93.7) | 90.6 (77.7, 97.0) | 86.1 (54.5, 98.5) | 77.3 (60.3, 88.4) | 96.8 (89.7, 99.7) |
| ONDO | AKURE SOUTH | 79.6 (59.4, 92.8) | 89.0 (74.0, 96.7) | 84.5 (49.3, 98.1) | 72.8 (52.9, 86.8) | 95.7 (85.3, 99.6) |
| ONDO | ESE ODO | 41.4 (20.4, 66.7) | 89.4 (76.6, 96.9) | 87.8 (69.0, 97.2) | 39.1 (19.7, 61.3) | 91.8 (79.0, 98.2) |
| ONDO | IDANRE | 73.9 (55.1, 88.4) | 89.4 (79.4, 95.7) | 82.8 (58.7, 95.9) | 69.2 (50.5, 84.0) | 94.2 (85.1, 98.7) |
| ONDO | IFEDORE | 80.3 (61.2, 93.2) | 94.1 (85.4, 98.5) | 90.0 (65.1, 98.9) | 77.2 (58.8, 90.0) | 97.2 (89.7, 99.7) |
| ONDO | ILAJE | 27.4 (13.7, 46.0) | 92.5 (83.5, 97.7) | 90.2 (79.5, 95.2) | 26.3 (12.8, 44.4) | 93.7 (85.0, 98.4) |
| ONDO | ILE OLUJI | 76.4 (53.0, 92.2) | 92.3 (80.3, 97.9) | 90.1 (68.4, 99.1) | 71.9 (48.5, 88.0) | 96.8 (88.7, 99.7) |
| ONDO | IRELE | 59.9 (38.7, 78.9) | 90.7 (79.3, 96.8) | 85.8 (60.5, 96.9) | 57.1 (36.7, 75.2) | 93.5 (82.7, 98.6) |
| ONDO | ODIGBO | 59.1 (31.7, 82.9) | 89.8 (78.6, 96.4) | 86.7 (61.4, 97.5) | 55.6 (29.6, 76.7) | 93.3 (82.0, 98.8) |
| ONDO | OKITIPUPA | 49.2 (28.2, 69.5) | 93.0 (83.2, 98.3) | 92.0 (75.5, 98.8) | 47.0 (27.4, 66.8) | 95.2 (85.6, 99.3) |
| ONDO | ONDO EAST | 67.7 (41.0, 88.3) | 91.6 (80.3, 97.8) | 89.3 (66.6, 98.6) | 63.7 (38.8, 84.0) | 95.5 (85.4, 99.5) |
| ONDO | ONDO WEST | 61.7 (36.5, 82.5) | 89.1 (76.5, 96.4) | 90.2 (67.8, 98.6) | 55.5 (32.5, 75.6) | 95.2 (84.8, 99.4) |
| ONDO | OSE | 80.6 (63.4, 93.3) | 92.5 (84.1, 97.3) | 87.9 (67.1, 98.2) | 76.2 (59.9, 89.1) | 96.9 (90.5, 99.6) |
| ONDO | OWO | 83.1 (61.9, 95.5) | 92.5 (81.7, 98.1) | 87.6 (58.5, 98.9) | 78.5 (56.8, 92.0) | 97.1 (89.9, 99.8) |
| OSUN | ATAKUMOSA EAST | 73.5 (55.2, 88.5) | 89.3 (77.8, 96.2) | 90.6 (72.4, 98.6) | 66.1 (48.9, 80.3) | 96.8 (89.1, 99.6) |
| OSUN | ATAKUMOSA WEST | 75.0 (57.1, 87.8) | 89.2 (79.4, 95.7) | 90.1 (71.6, 98.6) | 67.6 (50.7, 81.4) | 96.6 (89.6, 99.5) |
| OSUN | AYEDAADE | 64.1 (45.6, 81.6) | 83.7 (71.3, 92.5) | 76.1 (52.1, 92.6) | 58.3 (41.0, 74.9) | 89.5 (78.1, 96.8) |
| OSUN | AYEDIRE | 71.8 (46.1, 89.7) | 82.0 (64.8, 93.8) | 73.2 (34.4, 95.9) | 63.1 (40.1, 82.3) | 90.7 (73.9, 98.7) |
| OSUN | BOLUWADURO | 81.6 (62.7, 93.6) | 88.3 (72.9, 96.3) | 90.1 (65.2, 99.3) | 72.5 (51.2, 86.5) | 97.4 (89.0, 99.8) |
| OSUN | BORIPE | 83.4 (66.5, 94.4) | 86.3 (72.6, 95.2) | 88.5 (58.8, 99.0) | 72.4 (54.6, 85.2) | 97.3 (90.2, 99.8) |
| OSUN | EDE NORTH | 76.8 (56.1, 90.7) | 85.9 (70.3, 95.0) | 89.1 (65.7, 99.0) | 66.2 (46.5, 82.1) | 96.6 (88.6, 99.7) |
| OSUN | EDE SOUTH | 70.8 (47.7, 88.2) | 88.7 (75.1, 96.2) | 90.6 (71.6, 98.8) | 63.2 (42.4, 80.4) | 96.3 (88.1, 99.6) |
| OSUN | EGBEDORE | 80.8 (63.8, 92.0) | 85.3 (71.8, 94.5) | 86.8 (59.7, 98.6) | 69.5 (52.7, 83.8) | 96.6 (89.0, 99.6) |
| OSUN | EJIGBO | 73.3 (51.0, 89.7) | 84.4 (69.9, 93.9) | 77.4 (44.1, 95.0) | 65.4 (45.3, 81.5) | 92.3 (79.9, 98.4) |
| OSUN | IFE EAST | 70.3 (45.7, 89.0) | 87.3 (69.3, 96.6) | 89.8 (58.3, 99.2) | 61.6 (39.4, 80.6) | 96.0 (83.4, 99.7) |
| OSUN | IFE NORTH | 60.9 (41.9, 77.4) | 89.0 (77.5, 95.7) | 89.3 (70.6, 97.8) | 55.1 (38.0, 70.9) | 94.8 (85.2, 99.0) |
| OSUN | IFE SOUTH | 69.0 (43.2, 88.3) | 85.4 (67.3, 95.7) | 88.9 (62.3, 98.8) | 58.9 (35.3, 78.6) | 95.5 (83.9, 99.6) |
| OSUN | IFE CENTRAL | 63.3 (40.1, 83.2) | 89.8 (77.0, 96.9) | 91.9 (71.6, 99.2) | 57.0 (35.9, 75.9) | 96.1 (85.6, 99.7) |
| OSUN | IFEDAYO | 89.0 (74.5, 97.0) | 93.6 (83.3, 98.3) | 88.6 (55.0, 99.3) | 84.3 (69.6, 93.9) | 98.2 (93.2, 99.9) |
| OSUN | IFELODUN | 85.5 (67.4, 95.9) | 83.5 (66.5, 94.1) | 88.0 (55.5, 99.1) | 71.5 (53.0, 85.3) | 97.6 (90.9, 99.8) |
| OSUN | ILA | 85.5 (70.5, 94.7) | 90.1 (78.9, 96.4) | 89.8 (62.3, 99.1) | 77.7 (61.4, 88.8) | 97.9 (92.7, 99.8) |
| OSUN | ILESA EAST | 79.5 (61.3, 91.8) | 88.7 (76.3, 96.2) | 85.1 (55.5, 98.3) | 72.4 (54.1, 85.6) | 95.8 (85.7, 99.6) |
| OSUN | ILESA WEST | 76.5 (56.0, 90.9) | 87.2 (72.5, 95.7) | 85.2 (54.7, 98.3) | 68.5 (49.1, 84.3) | 95.3 (83.1, 99.5) |
| OSUN | IREPODUN | 83.6 (62.4, 95.3) | 85.5 (69.3, 95.9) | 86.1 (49.6, 99.1) | 72.2 (50.6, 87.4) | 96.9 (89.0, 99.8) |
| OSUN | IREWOLE | 69.0 (44.4, 87.4) | 76.9 (58.6, 90.5) | 55.4 (21.6, 83.9) | 62.1 (39.1, 81.7) | 83.8 (66.1, 95.6) |
| OSUN | ISOKAN | 63.3 (38.4, 83.9) | 75.0 (56.8, 89.1) | 51.9 (22.7, 79.9) | 58.7 (36.2, 80.0) | 79.5 (60.0, 93.0) |
| OSUN | IWO | 70.8 (45.0, 89.0) | 78.1 (59.2, 91.2) | 70.0 (27.5, 94.7) | 59.7 (35.7, 78.0) | 89.3 (72.7, 98.2) |
| OSUN | OBOKUN | 82.6 (67.8, 92.9) | 90.2 (79.9, 96.6) | 87.5 (59.1, 98.3) | 75.9 (61.3, 87.5) | 97.0 (89.9, 99.6) |
| OSUN | ODOOTIN | 82.0 (66.1, 92.9) | 87.5 (76.2, 95.0) | 88.7 (60.8, 99.2) | 72.3 (55.9, 85.0) | 97.2 (89.6, 99.8) |
| OSUN | OLAOLUWA | 69.0 (43.6, 89.4) | 82.6 (64.3, 94.1) | 77.6 (42.4, 96.6) | 60.4 (38.1, 80.2) | 91.2 (75.7, 98.7) |
| OSUN | OLORUNDA | 83.1 (59.9, 96.0) | 85.2 (66.2, 96.5) | 87.6 (52.8, 99.2) | 71.0 (48.6, 88.1) | 97.2 (88.9, 99.9) |
| OSUN | ORIADE | 84.1 (71.9, 92.6) | 92.8 (86.2, 97.2) | 87.4 (62.7, 98.2) | 79.7 (67.8, 88.6) | 97.2 (91.4, 99.6) |
| OSUN | OROLU | 85.4 (64.5, 96.6) | 85.8 (67.4, 96.4) | 84.1 (45.0, 99.1) | 74.4 (50.2, 89.9) | 96.9 (88.5, 99.8) |
| OSUN | OSOGBO | 81.3 (56.8, 95.3) | 85.3 (66.0, 96.3) | 87.7 (52.4, 99.2) | 69.7 (47.0, 87.5) | 96.9 (88.1, 99.9) |
| OYO | AFIJIO | 56.4 (34.2, 76.9) | 81.2 (64.5, 92.6) | 76.1 (49.2, 93.7) | 50.1 (30.4, 69.8) | 87.5 (71.9, 96.6) |
| OYO | AKINYELE | 66.7 (46.5, 84.0) | 81.6 (67.4, 91.6) | 72.2 (45.7, 91.0) | 59.6 (41.3, 77.0) | 88.6 (75.2, 96.7) |
| OYO | ATIBA | 57.2 (27.2, 82.5) | 83.1 (65.1, 93.5) | 76.4 (45.6, 94.4) | 52.1 (24.0, 76.3) | 88.2 (70.0, 97.2) |
| OYO | ATISBO | 53.8 (29.4, 74.9) | 87.8 (75.8, 95.5) | 83.7 (58.6, 95.6) | 50.6 (27.9, 70.1) | 91.0 (78.7, 97.5) |
| OYO | EGBEDA | 75.1 (55.9, 89.7) | 81.6 (66.7, 92.2) | 58.8 (23.3, 84.5) | 68.7 (51.4, 84.2) | 88.0 (73.5, 96.5) |
| OYO | IBADAN NORTH | 75.4 (59.4, 88.8) | 84.0 (68.6, 93.7) | 66.1 (33.1, 87.7) | 69.2 (52.8, 83.6) | 90.2 (77.1, 97.5) |
| OYO | IBADAN NORTH EAST | 79.9 (63.9, 91.4) | 84.3 (70.1, 93.5) | 48.9 (17.3, 73.6) | 75.0 (59.2, 87.6) | 89.1 (75.7, 96.5) |
| OYO | IBADAN NORTH WEST | 76.2 (58.9, 89.7) | 83.7 (69.2, 93.4) | 68.8 (32.1, 91.8) | 69.2 (51.7, 83.5) | 90.7 (77.4, 97.8) |
| OYO | IBADAN SOUTH EAST | 76.7 (57.9, 90.7) | 83.1 (68.2, 93.3) | 58.3 (23.9, 82.7) | 70.9 (52.2, 85.9) | 88.9 (74.9, 97.0) |
| OYO | IBADAN SOUTH WEST | 74.2 (55.3, 88.7) | 84.1 (69.6, 93.6) | 71.4 (36.1, 92.2) | 67.5 (48.5, 82.4) | 90.7 (77.7, 97.7) |
| OYO | IBARAPA CENTRAL | 71.8 (45.9, 90.2) | 82.7 (66.8, 94.6) | 70.8 (31.0, 95.3) | 64.6 (41.9, 83.4) | 89.8 (73.2, 98.3) |
| OYO | IBARAPA EAST | 76.4 (49.0, 94.9) | 88.5 (72.1, 97.0) | 79.0 (38.2, 97.7) | 71.1 (43.8, 89.1) | 93.8 (79.2, 99.4) |
| OYO | IBARAPA NORTH | 70.3 (45.1, 91.0) | 86.6 (71.7, 96.1) | 77.1 (38.3, 96.4) | 65.1 (41.5, 85.2) | 91.8 (76.9, 98.9) |
| OYO | IDO | 69.7 (48.3, 88.2) | 82.5 (67.6, 92.2) | 72.4 (42.7, 91.5) | 62.5 (42.4, 80.3) | 89.7 (76.0, 97.2) |
| OYO | IREPO | 57.8 (24.0, 87.3) | 77.4 (53.0, 92.7) | 70.2 (30.0, 95.2) | 49.9 (20.8, 77.1) | 85.3 (61.7, 97.8) |
| OYO | ISEYIN | 63.8 (29.1, 92.0) | 86.6 (68.0, 96.2) | 80.8 (45.7, 97.6) | 58.8 (26.1, 85.6) | 91.7 (72.0, 99.2) |
| OYO | ITESIWAJU | 57.1 (25.9, 83.9) | 87.8 (72.0, 96.6) | 84.5 (57.4, 97.6) | 52.8 (23.8, 77.8) | 92.1 (76.2, 98.9) |
| OYO | IWAJOWA | 49.7 (23.7, 74.1) | 89.1 (75.2, 96.8) | 86.5 (64.1, 97.3) | 46.8 (23.0, 70.0) | 92.0 (79.1, 98.5) |
| OYO | KAJOLA | 61.1 (23.5, 91.6) | 87.8 (67.1, 97.2) | 85.0 (49.6, 99.0) | 56.1 (22.0, 86.0) | 92.9 (73.1, 99.6) |
| OYO | LAGELU | 72.1 (52.8, 87.4) | 80.4 (64.9, 91.9) | 64.9 (29.9, 89.1) | 64.5 (47.5, 80.1) | 88.0 (74.6, 96.7) |
| OYO | OGBOMOSO NORTH | 76.8 (47.9, 93.8) | 85.1 (66.9, 95.6) | 77.3 (37.8, 96.9) | 68.7 (43.3, 87.3) | 93.2 (78.2, 99.4) |
| OYO | OGBOMOSO SOUTH | 74.3 (46.7, 92.2) | 86.2 (69.6, 96.1) | 78.3 (43.7, 97.1) | 67.7 (43.1, 86.2) | 92.8 (78.1, 99.2) |
| OYO | OGO OLUWA | 70.2 (50.7, 87.0) | 85.3 (72.6, 93.6) | 77.1 (53.0, 92.6) | 64.2 (45.5, 80.2) | 91.4 (80.1, 97.8) |
| OYO | OLORUNSOGO | 63.4 (38.8, 83.2) | 73.3 (54.5, 86.5) | 58.7 (29.9, 83.2) | 54.3 (32.3, 73.7) | 82.4 (63.7, 94.0) |
| OYO | OLUYOLE | 66.4 (44.2, 84.7) | 84.6 (69.7, 94.4) | 73.7 (40.0, 94.3) | 61.1 (41.0, 79.0) | 89.9 (75.2, 97.8) |
| OYO | ONA ORA | 72.0 (50.1, 88.2) | 81.6 (64.9, 92.5) | 61.8 (26.5, 87.7) | 66.4 (46.7, 82.2) | 87.1 (70.5, 96.5) |
| OYO | OORELOPE | 53.2 (22.4, 82.5) | 77.9 (54.3, 93.0) | 72.0 (31.8, 95.3) | 46.2 (19.1, 73.8) | 84.9 (60.2, 97.8) |
| OYO | ORIRE | 64.6 (38.0, 86.2) | 83.6 (68.9, 93.6) | 73.8 (44.5, 92.7) | 59.4 (35.2, 79.2) | 88.8 (73.4, 97.3) |
| OYO | OYO EAST | 56.5 (33.1, 76.6) | 80.4 (62.9, 92.5) | 76.6 (48.2, 94.4) | 49.1 (28.6, 68.7) | 87.8 (71.9, 97.2) |
| OYO | OYO WEST | 59.6 (37.1, 79.8) | 78.9 (60.4, 91.6) | 74.5 (44.2, 93.4) | 50.8 (31.0, 71.1) | 87.7 (70.4, 97.2) |
| OYO | SAKI EAST | 59.9 (31.0, 85.6) | 86.7 (67.4, 96.1) | 81.9 (47.7, 96.9) | 55.3 (29.3, 79.3) | 91.2 (73.8, 98.7) |
| OYO | SAKI WEST | 58.5 (28.1, 84.5) | 88.5 (73.9, 96.5) | 84.0 (54.3, 97.2) | 55.0 (25.8, 79.0) | 92.1 (76.4, 98.7) |
| OYO | SURULERE | 74.6 (51.7, 89.9) | 87.1 (73.8, 95.3) | 79.9 (51.9, 95.5) | 68.3 (45.4, 84.9) | 93.4 (82.9, 98.8) |
| PLATEAU | BARKIN LADI | 69.6 (46.9, 87.0) | 95.7 (88.4, 99.1) | 93.0 (74.9, 99.3) | 68.1 (45.3, 85.2) | 97.2 (90.5, 99.7) |
| PLATEAU | BASSA | 49.3 (26.5, 72.5) | 91.8 (80.6, 97.8) | 89.5 (72.1, 98.2) | 47.8 (25.4, 70.0) | 93.3 (82.0, 98.8) |
| PLATEAU | BOKKOS | 69.1 (48.1, 85.7) | 94.9 (86.2, 98.7) | 91.3 (70.4, 99.0) | 67.6 (47.0, 83.9) | 96.4 (87.9, 99.6) |
| PLATEAU | JOS EAST | 66.0 (39.8, 87.2) | 95.6 (87.9, 99.1) | 93.2 (76.2, 99.4) | 64.6 (38.9, 85.5) | 97.0 (89.3, 99.8) |
| PLATEAU | JOS NORTH | 63.7 (39.4, 84.2) | 94.8 (85.1, 98.9) | 93.5 (77.4, 99.4) | 61.6 (38.8, 82.2) | 96.9 (88.9, 99.7) |
| PLATEAU | JOS SOUTH | 64.3 (41.6, 83.7) | 95.4 (86.9, 99.0) | 93.9 (78.4, 99.4) | 62.6 (40.1, 82.1) | 97.1 (89.8, 99.7) |
| PLATEAU | KANAM | 64.8 (38.0, 85.8) | 91.0 (79.4, 97.0) | 85.5 (62.0, 97.0) | 62.2 (36.9, 81.8) | 93.6 (81.1, 98.7) |
| PLATEAU | KANKE | 71.9 (52.9, 86.1) | 92.5 (84.0, 97.5) | 87.5 (67.2, 97.6) | 69.2 (50.8, 83.2) | 95.2 (87.4, 99.0) |
| PLATEAU | LANGTANG NORTH | 67.5 (43.8, 85.0) | 88.4 (76.9, 95.5) | 82.9 (61.2, 95.2) | 63.0 (41.1, 80.0) | 92.8 (82.7, 98.0) |
| PLATEAU | LANGTANG SOUTH | 62.7 (30.1, 87.9) | 89.3 (71.9, 97.3) | 83.1 (53.0, 97.6) | 59.7 (29.3, 83.6) | 92.3 (75.9, 99.0) |
| PLATEAU | MANGU | 73.5 (57.7, 86.7) | 95.8 (89.6, 98.9) | 92.9 (78.7, 98.9) | 71.9 (56.2, 85.1) | 97.4 (91.8, 99.6) |
| PLATEAU | MIKANG | 73.3 (54.9, 87.3) | 92.4 (84.0, 97.3) | 86.6 (68.0, 97.4) | 70.6 (52.9, 84.1) | 95.1 (86.9, 99.1) |
| PLATEAU | PANKSHIN | 74.3 (51.7, 90.7) | 94.7 (86.6, 98.6) | 91.4 (71.7, 98.9) | 72.1 (50.8, 87.7) | 96.9 (89.5, 99.7) |
| PLATEAU | QUAANPAN | 71.3 (49.2, 88.8) | 93.5 (84.9, 98.1) | 88.6 (68.4, 98.3) | 69.2 (48.4, 86.1) | 95.7 (87.7, 99.5) |
| PLATEAU | RIYOM | 61.2 (32.3, 86.2) | 93.5 (81.2, 98.5) | 90.7 (69.6, 99.0) | 59.2 (31.0, 83.9) | 95.4 (84.0, 99.5) |
| PLATEAU | SHENDAM | 68.1 (49.1, 83.7) | 92.2 (84.0, 97.1) | 86.8 (68.2, 96.5) | 65.7 (47.5, 80.9) | 94.5 (86.4, 98.7) |
| PLATEAU | WASE | 59.7 (34.8, 83.5) | 88.3 (76.6, 95.5) | 83.8 (62.7, 95.7) | 55.9 (32.9, 77.9) | 92.1 (81.5, 98.1) |
| RIVERS | ABUA/ODUAL | 61.7 (38.6, 81.5) | 86.0 (73.0, 94.5) | 79.2 (55.8, 94.1) | 57.6 (35.9, 76.2) | 90.1 (77.1, 97.6) |
| RIVERS | AHOADA EAST | 60.2 (35.8, 83.0) | 87.5 (73.3, 96.2) | 84.4 (58.1, 97.7) | 55.4 (32.3, 76.3) | 92.3 (77.7, 98.8) |
| RIVERS | AHOADA WEST | 56.9 (32.8, 78.3) | 88.8 (75.8, 95.9) | 87.5 (65.7, 97.8) | 52.3 (30.1, 72.2) | 93.3 (81.4, 98.8) |
| RIVERS | AKUKU TORU | 70.2 (39.8, 92.7) | 94.1 (82.3, 98.9) | 91.5 (70.1, 99.1) | 67.7 (38.5, 89.8) | 96.6 (86.3, 99.7) |
| RIVERS | ANDONI | 70.7 (44.2, 89.6) | 93.5 (82.5, 98.4) | 89.4 (64.9, 97.8) | 68.0 (42.8, 86.5) | 96.2 (87.2, 99.7) |
| RIVERS | ASARI TORU | 66.1 (39.6, 87.2) | 91.8 (81.1, 97.8) | 88.7 (69.1, 98.2) | 62.9 (37.5, 83.9) | 95.0 (84.8, 99.3) |
| RIVERS | BONNY | 68.6 (41.8, 86.9) | 93.5 (84.5, 98.4) | 87.9 (68.6, 95.8) | 66.2 (41.0, 84.3) | 95.9 (86.5, 99.6) |
| RIVERS | DEGEMA | 65.6 (36.3, 88.0) | 93.0 (82.9, 98.1) | 89.8 (68.7, 97.9) | 63.1 (35.4, 84.8) | 95.4 (86.2, 99.1) |
| RIVERS | ELEME | 71.0 (47.4, 88.3) | 87.4 (72.5, 96.1) | 76.3 (41.9, 95.3) | 67.1 (44.7, 84.4) | 91.4 (76.7, 98.4) |
| RIVERS | EMOHUA | 60.5 (36.9, 80.0) | 86.1 (72.7, 94.6) | 81.2 (55.9, 95.0) | 55.8 (34.6, 73.7) | 90.8 (76.8, 97.7) |
| RIVERS | ETCHE | 59.6 (31.5, 82.6) | 82.7 (62.7, 94.2) | 73.2 (40.8, 94.0) | 55.1 (29.5, 77.2) | 87.2 (68.3, 97.3) |
| RIVERS | GOKANA | 64.9 (43.2, 82.1) | 93.0 (83.9, 98.0) | 89.4 (72.3, 97.2) | 62.2 (41.4, 79.6) | 95.7 (87.2, 99.3) |
| RIVERS | IKWERRE | 62.5 (35.3, 85.2) | 84.8 (69.1, 95.0) | 78.5 (49.1, 95.6) | 57.1 (33.1, 78.8) | 90.3 (74.5, 98.3) |
| RIVERS | KHANA | 67.0 (46.6, 83.4) | 91.2 (81.3, 96.8) | 87.6 (69.0, 96.8) | 63.2 (44.1, 79.1) | 95.0 (86.1, 98.9) |
| RIVERS | OBIO/AKPOR | 68.9 (44.5, 88.9) | 83.7 (66.2, 94.0) | 67.7 (35.1, 88.1) | 63.7 (40.8, 82.7) | 88.9 (70.7, 97.8) |
| RIVERS | OGBA/EGBEMA/NDONI | 63.8 (37.7, 84.7) | 83.7 (68.0, 94.1) | 81.2 (55.7, 95.6) | 55.8 (33.1, 75.2) | 91.7 (78.4, 98.3) |
| RIVERS | OGU/BOLO | 64.1 (39.8, 83.9) | 92.6 (81.0, 98.1) | 89.1 (65.9, 98.4) | 61.6 (38.0, 81.4) | 95.0 (83.3, 99.4) |
| RIVERS | OKRIKA | 68.6 (42.8, 88.2) | 91.0 (79.0, 97.6) | 84.4 (55.0, 97.7) | 65.6 (40.7, 84.9) | 94.0 (82.3, 99.2) |
| RIVERS | OMUMA | 53.7 (29.0, 75.3) | 81.7 (64.4, 92.9) | 71.9 (43.1, 90.7) | 50.8 (28.3, 71.4) | 84.6 (66.3, 95.3) |
| RIVERS | OPOBO/NKORO | 71.3 (46.5, 89.5) | 92.8 (81.2, 98.4) | 87.2 (57.0, 96.9) | 68.4 (44.8, 86.1) | 95.7 (84.2, 99.7) |
| RIVERS | OYIGBO | 62.9 (43.9, 79.5) | 83.0 (69.6, 92.3) | 71.7 (48.6, 89.8) | 58.6 (40.7, 74.3) | 87.2 (74.7, 95.7) |
| RIVERS | PORT HARCOURT | 69.3 (42.0, 89.4) | 86.9 (71.3, 96.2) | 70.9 (36.1, 90.8) | 65.6 (40.3, 85.3) | 90.6 (74.7, 98.5) |
| RIVERS | TAI | 64.6 (43.2, 82.3) | 90.7 (79.6, 96.9) | 84.1 (61.8, 94.9) | 61.9 (41.7, 79.6) | 93.4 (82.5, 98.5) |
| SOKOTO | BINJI | 18.4 (06.6, 36.2) | 89.6 (75.0, 96.9) | 89.0 (72.7, 97.3) | 17.7 (06.5, 34.7) | 90.3 (75.7, 97.5) |
| SOKOTO | BODINGA | 35.7 (14.9, 61.7) | 81.0 (60.2, 94.2) | 75.6 (46.8, 93.4) | 34.7 (14.7, 59.6) | 82.0 (60.7, 95.1) |
| SOKOTO | DANGE | 28.2 (13.0, 47.9) | 75.3 (59.2, 87.4) | 70.0 (48.7, 85.5) | 27.2 (12.7, 45.5) | 76.3 (59.8, 88.6) |
| SOKOTO | GADA | 55.8 (29.3, 79.8) | 89.7 (75.0, 97.2) | 83.8 (55.2, 96.9) | 54.0 (28.5, 77.5) | 91.4 (76.2, 98.4) |
| SOKOTO | GORONYO | 45.7 (24.2, 67.1) | 90.6 (79.6, 97.0) | 87.4 (70.3, 96.8) | 44.4 (23.9, 65.3) | 91.8 (80.7, 98.0) |
| SOKOTO | GUDU | 28.8 (09.1, 57.2) | 84.5 (67.2, 94.8) | 81.5 (59.2, 94.8) | 27.8 (08.8, 54.3) | 85.5 (67.4, 95.9) |
| SOKOTO | GWADABAWA | 51.8 (27.9, 76.4) | 87.9 (72.7, 96.2) | 81.7 (56.0, 96.0) | 50.2 (26.7, 73.8) | 89.5 (74.9, 97.7) |
| SOKOTO | ILLELA | 48.6 (19.0, 78.9) | 90.2 (73.2, 97.9) | 86.2 (60.1, 97.9) | 47.2 (18.7, 75.4) | 91.5 (74.3, 98.9) |
| SOKOTO | ISA | 29.4 (09.3, 58.5) | 90.6 (75.1, 97.7) | 89.3 (68.6, 98.1) | 28.4 (08.8, 56.9) | 91.6 (75.6, 98.6) |
| SOKOTO | KEBBE | 36.5 (17.0, 62.0) | 84.1 (67.0, 93.8) | 79.5 (56.5, 92.7) | 35.1 (16.4, 59.4) | 85.6 (68.3, 95.0) |
| SOKOTO | KWARE | 30.9 (15.8, 48.5) | 79.9 (65.1, 90.0) | 75.6 (56.7, 88.8) | 29.8 (15.4, 46.4) | 81.1 (66.3, 91.1) |
| SOKOTO | RABAH | 29.4 (11.0, 54.4) | 85.8 (68.5, 95.3) | 83.2 (61.3, 95.1) | 28.2 (10.5, 52.9) | 87.0 (69.3, 96.3) |
| SOKOTO | SABON-BIRNI | 33.7 (10.7, 61.5) | 90.3 (74.4, 97.6) | 88.4 (67.2, 98.2) | 32.6 (10.5, 59.2) | 91.4 (75.0, 98.6) |
| SOKOTO | SHAGARI | 34.9 (14.1, 61.5) | 87.0 (69.4, 96.3) | 84.3 (59.4, 96.3) | 33.6 (14.0, 59.8) | 88.3 (71.1, 97.2) |
| SOKOTO | SILAME | 19.1 (05.5, 40.1) | 86.3 (67.5, 96.2) | 84.8 (63.8, 96.3) | 18.6 (05.2, 39.4) | 86.8 (68.1, 96.8) |
| SOKOTO | SOKOTO | 24.1 (10.0, 43.7) | 72.1 (53.8, 86.8) | 67.7 (46.5, 84.6) | 22.7 (09.3, 41.7) | 73.5 (54.8, 88.0) |
| SOKOTO | SOKOTO SOUTH | 23.5 (10.0, 42.1) | 70.0 (52.4, 84.4) | 65.2 (45.7, 81.7) | 22.2 (09.4, 40.8) | 71.3 (54.0, 85.8) |
| SOKOTO | TAMBAWAL | 27.1 (11.5, 47.6) | 91.2 (79.6, 97.4) | 90.2 (76.3, 97.7) | 26.2 (11.4, 45.8) | 92.1 (80.8, 98.1) |
| SOKOTO | TANGAZA | 38.1 (20.9, 58.0) | 86.0 (72.9, 94.6) | 81.3 (62.5, 93.7) | 37.0 (20.4, 55.4) | 87.1 (73.8, 95.8) |
| SOKOTO | TURETA | 28.5 (06.0, 61.0) | 85.3 (60.0, 97.2) | 82.7 (50.4, 97.3) | 27.5 (05.8, 58.1) | 86.4 (60.5, 97.9) |
| SOKOTO | WAMAKKO | 25.5 (11.6, 42.9) | 74.4 (58.2, 86.4) | 69.7 (50.7, 84.4) | 24.3 (11.1, 41.6) | 75.6 (58.8, 87.5) |
| SOKOTO | WURNO | 46.9 (24.6, 70.5) | 89.2 (75.3, 96.6) | 85.7 (63.9, 96.9) | 45.1 (24.1, 67.6) | 91.0 (76.8, 98.0) |
| SOKOTO | YABO | 38.8 (16.2, 68.6) | 89.7 (71.9, 97.8) | 86.8 (61.2, 97.8) | 37.7 (15.8, 66.6) | 90.7 (72.3, 98.6) |
| TARABA | ARDO KOLA | 63.0 (41.0, 81.2) | 82.5 (68.7, 92.5) | 72.1 (46.5, 89.9) | 57.9 (37.9, 75.0) | 87.7 (73.8, 96.0) |
| TARABA | BALI | 59.8 (33.5, 82.9) | 89.8 (77.9, 96.0) | 85.1 (67.2, 95.7) | 56.9 (32.3, 79.1) | 92.7 (81.0, 98.4) |
| TARABA | DONGA | 62.8 (40.0, 82.8) | 90.7 (79.5, 96.7) | 84.6 (63.2, 96.0) | 60.6 (38.0, 79.9) | 92.9 (82.4, 98.3) |
| TARABA | GASHAKA | 55.5 (22.1, 82.0) | 90.4 (79.6, 96.6) | 87.4 (68.9, 97.2) | 52.7 (21.0, 77.9) | 93.3 (82.1, 98.7) |
| TARABA | GASSOL | 59.0 (37.6, 78.4) | 91.4 (82.9, 96.8) | 87.4 (72.1, 96.5) | 56.7 (36.3, 75.7) | 93.6 (85.2, 98.3) |
| TARABA | IBI | 60.9 (37.3, 80.3) | 90.9 (79.7, 97.0) | 85.5 (62.4, 96.6) | 58.6 (35.9, 77.8) | 93.1 (82.1, 98.6) |
| TARABA | JALINGO | 61.1 (34.3, 84.7) | 81.2 (62.5, 92.9) | 72.2 (38.5, 93.5) | 55.1 (30.4, 77.1) | 87.2 (69.6, 97.1) |
| TARABA | KARIM-LAMIDO | 59.0 (42.2, 75.6) | 91.4 (83.2, 96.6) | 88.4 (74.4, 96.9) | 56.2 (39.4, 72.2) | 94.2 (85.6, 98.4) |
| TARABA | KURMI | 61.4 (41.3, 79.4) | 92.5 (83.5, 97.4) | 88.8 (71.6, 97.4) | 59.2 (39.4, 76.8) | 94.7 (86.0, 99.0) |
| TARABA | LAU | 44.0 (21.1, 68.8) | 88.5 (74.8, 96.0) | 85.5 (63.7, 97.0) | 41.8 (20.4, 64.8) | 90.8 (76.1, 98.2) |
| TARABA | SARDAUNA | 31.1 (11.2, 56.5) | 92.3 (81.6, 98.0) | 91.6 (78.2, 98.4) | 29.7 (10.8, 53.6) | 93.7 (83.6, 98.8) |
| TARABA | TAKUM | 75.2 (50.5, 92.5) | 92.9 (82.4, 98.1) | 85.7 (59.1, 97.7) | 72.8 (48.7, 89.4) | 95.3 (86.0, 99.4) |
| TARABA | USSA | 85.9 (70.9, 94.9) | 94.4 (88.1, 98.0) | 87.5 (62.0, 98.3) | 82.8 (68.2, 92.4) | 97.6 (92.9, 99.7) |
| TARABA | WUKARI | 59.3 (36.2, 79.1) | 89.9 (78.0, 96.3) | 84.1 (64.1, 95.7) | 57.0 (35.3, 75.7) | 92.1 (80.4, 98.0) |
| TARABA | YORRO | 52.0 (32.3, 70.7) | 87.7 (75.9, 95.1) | 83.9 (65.2, 95.4) | 49.1 (30.7, 67.1) | 90.6 (78.7, 97.6) |
| TARABA | ZING | 50.8 (18.0, 80.1) | 86.9 (67.6, 97.1) | 82.7 (55.7, 97.8) | 48.0 (17.4, 76.0) | 89.7 (70.6, 98.8) |
| YOBE | BADE | 36.6 (18.6, 57.9) | 83.2 (66.3, 93.6) | 77.4 (56.0, 92.1) | 35.2 (17.7, 56.1) | 84.6 (67.9, 94.8) |
| YOBE | BURSARI | 44.2 (26.0, 62.6) | 88.9 (79.9, 95.1) | 84.3 (69.8, 93.5) | 42.8 (25.2, 60.1) | 90.3 (80.5, 96.2) |
| YOBE | DAMATURU | 56.0 (24.0, 85.2) | 89.0 (72.1, 97.0) | 84.1 (55.4, 96.8) | 53.4 (22.6, 81.7) | 91.6 (74.7, 98.7) |
| YOBE | FIKA | 57.6 (35.6, 77.8) | 90.8 (78.7, 97.1) | 84.9 (61.2, 96.5) | 56.0 (34.6, 75.5) | 92.4 (80.1, 98.3) |
| YOBE | FUNE | 56.2 (32.4, 77.3) | 91.3 (82.2, 96.8) | 86.4 (69.1, 96.3) | 54.6 (31.4, 74.9) | 92.9 (83.5, 98.4) |
| YOBE | GEIDAM | 44.2 (16.1, 77.5) | 89.7 (73.3, 97.2) | 86.7 (64.5, 97.6) | 42.4 (15.8, 73.8) | 91.5 (75.7, 98.6) |
| YOBE | GUJBA | 52.4 (23.3, 81.3) | 86.7 (70.6, 95.7) | 80.9 (52.9, 95.6) | 49.9 (22.5, 77.4) | 89.2 (72.1, 97.8) |
| YOBE | GULANI | 56.4 (34.7, 75.6) | 86.8 (76.1, 94.3) | 83.8 (63.1, 95.3) | 51.8 (32.3, 69.4) | 91.3 (80.6, 97.5) |
| YOBE | JAKUSKO | 52.4 (28.1, 75.2) | 86.9 (73.1, 95.3) | 81.4 (57.5, 95.0) | 49.7 (26.4, 71.2) | 89.5 (75.4, 97.3) |
| YOBE | KARASUWA | 28.5 (15.4, 43.6) | 83.1 (70.8, 92.3) | 77.4 (62.3, 89.2) | 27.3 (14.4, 42.1) | 84.3 (71.8, 93.5) |
| YOBE | MACHINA | 63.9 (38.0, 84.3) | 93.8 (82.7, 98.4) | 89.8 (67.9, 98.7) | 62.3 (37.5, 82.5) | 95.4 (85.2, 99.4) |
| YOBE | NANGERE | 72.6 (51.1, 88.8) | 86.9 (72.9, 94.8) | 65.7 (35.7, 88.6) | 70.4 (49.5, 86.3) | 89.0 (75.1, 96.7) |
| YOBE | NGURU | 26.6 (06.0, 57.5) | 89.3 (66.7, 98.2) | 88.0 (60.4, 98.6) | 25.6 (05.7, 54.8) | 90.3 (67.7, 98.9) |
| YOBE | POTISKUM | 71.2 (52.1, 86.3) | 88.9 (77.3, 96.0) | 69.9 (41.7, 90.8) | 69.4 (50.9, 84.5) | 90.7 (78.8, 97.2) |
| YOBE | TARMUA | 57.7 (31.9, 80.4) | 88.9 (76.6, 95.8) | 84.0 (59.2, 95.8) | 55.1 (30.2, 77.2) | 91.5 (79.3, 97.8) |
| YOBE | YUNUSARI | 40.3 (19.2, 64.4) | 90.7 (78.8, 96.6) | 88.8 (71.4, 96.9) | 38.6 (18.4, 61.1) | 92.4 (81.3, 98.2) |
| YOBE | YUSUFARI | 29.4 (13.4, 51.0) | 85.8 (71.1, 94.8) | 82.5 (63.9, 94.0) | 28.3 (13.0, 48.4) | 86.8 (72.1, 95.5) |
| ZAMFARA | ANKA | 20.3 (06.8, 41.5) | 90.5 (77.4, 97.3) | 89.7 (75.0, 97.5) | 19.8 (06.5, 40.3) | 91.1 (78.1, 97.8) |
| ZAMFARA | BAKURA | 20.3 (05.4, 44.1) | 90.0 (75.8, 97.8) | 89.3 (72.6, 98.0) | 19.5 (05.3, 41.9) | 90.8 (76.5, 98.2) |
| ZAMFARA | BIRNIN MAGAJI | 24.4 (09.0, 46.4) | 87.2 (69.5, 96.5) | 85.1 (64.0, 96.3) | 23.8 (08.9, 45.1) | 87.8 (70.3, 97.1) |
| ZAMFARA | BUKKUYUM | 19.7 (07.6, 39.6) | 78.9 (63.2, 91.8) | 76.8 (58.4, 91.4) | 19.2 (07.3, 37.8) | 79.5 (63.6, 92.2) |
| ZAMFARA | BUNGUDU | 20.3 (07.6, 40.0) | 89.3 (76.0, 96.8) | 88.0 (71.1, 97.0) | 19.7 (07.4, 38.4) | 89.9 (76.4, 97.6) |
| ZAMFARA | GUMMI | 16.5 (05.6, 33.5) | 81.5 (67.4, 91.3) | 79.9 (63.5, 90.9) | 15.9 (05.5, 32.1) | 82.1 (68.0, 91.8) |
| ZAMFARA | GUSAU | 19.1 (07.8, 35.3) | 90.8 (81.0, 96.7) | 90.3 (79.0, 96.9) | 18.3 (07.7, 34.0) | 91.6 (81.4, 97.2) |
| ZAMFARA | KAURA-NAMODA | 36.3 (17.7, 59.8) | 76.4 (56.9, 90.8) | 66.9 (39.6, 87.7) | 35.1 (17.0, 56.9) | 77.5 (58.3, 91.9) |
| ZAMFARA | MARADUN | 21.6 (08.3, 43.5) | 83.2 (66.6, 94.2) | 80.6 (59.8, 94.1) | 20.9 (07.9, 42.1) | 83.9 (67.6, 95.0) |
| ZAMFARA | MARU | 23.3 (11.0, 39.3) | 82.5 (70.8, 91.2) | 79.8 (66.1, 90.4) | 22.4 (10.8, 37.7) | 83.4 (71.4, 92.1) |
| ZAMFARA | SHINKAFI | 23.1 (06.9, 48.7) | 90.2 (74.9, 97.4) | 89.0 (70.7, 97.6) | 22.4 (06.8, 46.1) | 90.8 (76.1, 98.0) |
| ZAMFARA | TALATA-MAFARA | 15.7 (04.5, 34.6) | 85.5 (69.3, 95.2) | 84.5 (66.8, 95.3) | 15.0 (04.4, 33.3) | 86.1 (69.9, 95.7) |
| ZAMFARA | TSAFE | 26.1 (11.8, 47.3) | 84.5 (69.9, 93.4) | 82.0 (64.3, 92.9) | 25.1 (11.3, 44.6) | 85.5 (70.8, 94.4) |
| ZAMFARA | ZURMI | 30.1 (11.8, 54.8) | 86.9 (71.4, 95.6) | 84.0 (63.2, 95.1) | 29.4 (11.6, 53.5) | 87.7 (71.9, 96.4) |

**Additional references**

[1] Fox J, Monette G. Generalized Collinearity Diagnostics. Journal of the American Statistical Association. 1992;87(417):178-83.

[2] Utazi CE, Thorley J, Alegana VA, Ferrari MJ, Takahashi S, Metcalf CJE, et al. High resolution age-structured mapping of childhood vaccination coverage in low and middle income countries. Vaccine. 2018;36(12):1583-91.

[3] Simpson D, Rue H, Riebler A, Martins TG, Sorbye SH. Penalising Model Component Complexity: A Principled, Practical Approach to Constructing Priors. Statist Sci. 2017;32(1):1-28.

[4] Fuglstad G-A, Simpson D, Lindgren F, Rue H. Constructing Priors that Penalize the Complexity of Gaussian Random Fields. Journal of the American Statistical Association. 2019;114(525):445-52.

[5] National Bureau of Statistics (NBS) and United Nations Children’s Fund (UNICEF). 2017 Multiple Indicator Cluster Survey 2016-17, Survey Findings Report. Abuja, Nigeria: National Bureau of Statistics and United Nations Children’s Fund; 2017.

[6] National Primary Healthcare Development Agency and National Bureau of Statistics. Nigeria, National Immunisation Coverage Survey 2016/17, Final Report. Abuja, Nigeria: National Primary Healthcare Development Agency and National Bureau of Statistics; 2017.
